# Supplementary material for: Insights from a methylome-wide association study of antidepressant exposure
Source: Nat Commun. 2025 Feb 24;16:1908. doi: 10.1038/s41467-024-55356-x (PMC11850842; doi:10.1038/s41467-024-55356-x)
Supplement: Supplementary file 1 — Supplementary Information [file 41467_2024_55356_MOESM1_ESM.pdf]

Supplementary Information

Insights from a Methylome-Wide Association Study of Antidepressant Exposure.

Davyson et al.

Supplementary Figures

Methylome wide association study

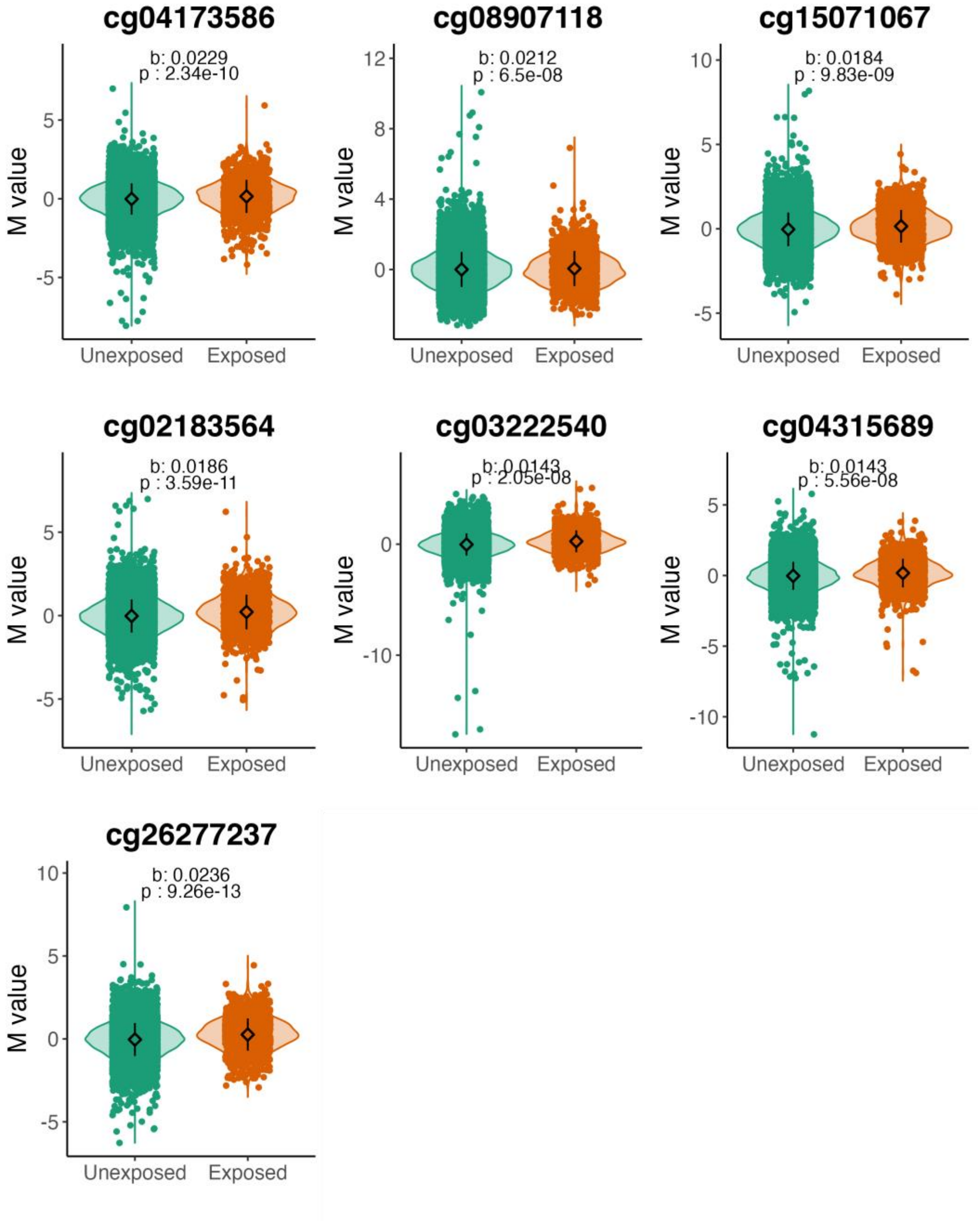

Supplementary Fig. 1: Distribution of M-values for CpGs significantly associated with prescription-derived antidepressant exposure.

Violin plots of the methylation M-values for significant probes ($p < 9.42x10^{-08}$) in the methylome-wide association study (MWAS) of self-report antidepressant exposure. Plots are grouped per probe by antidepressant exposure ($n_{exposed}$ = 1,508, $n_{unexposed}$ = 15,023). Overlaid box plots indicate the mean M-value +-

the standard deviation. b = Beta calculated in the self-report MWAS, p = p value calculated from the self-report MWAS (n = 16,531 participants).

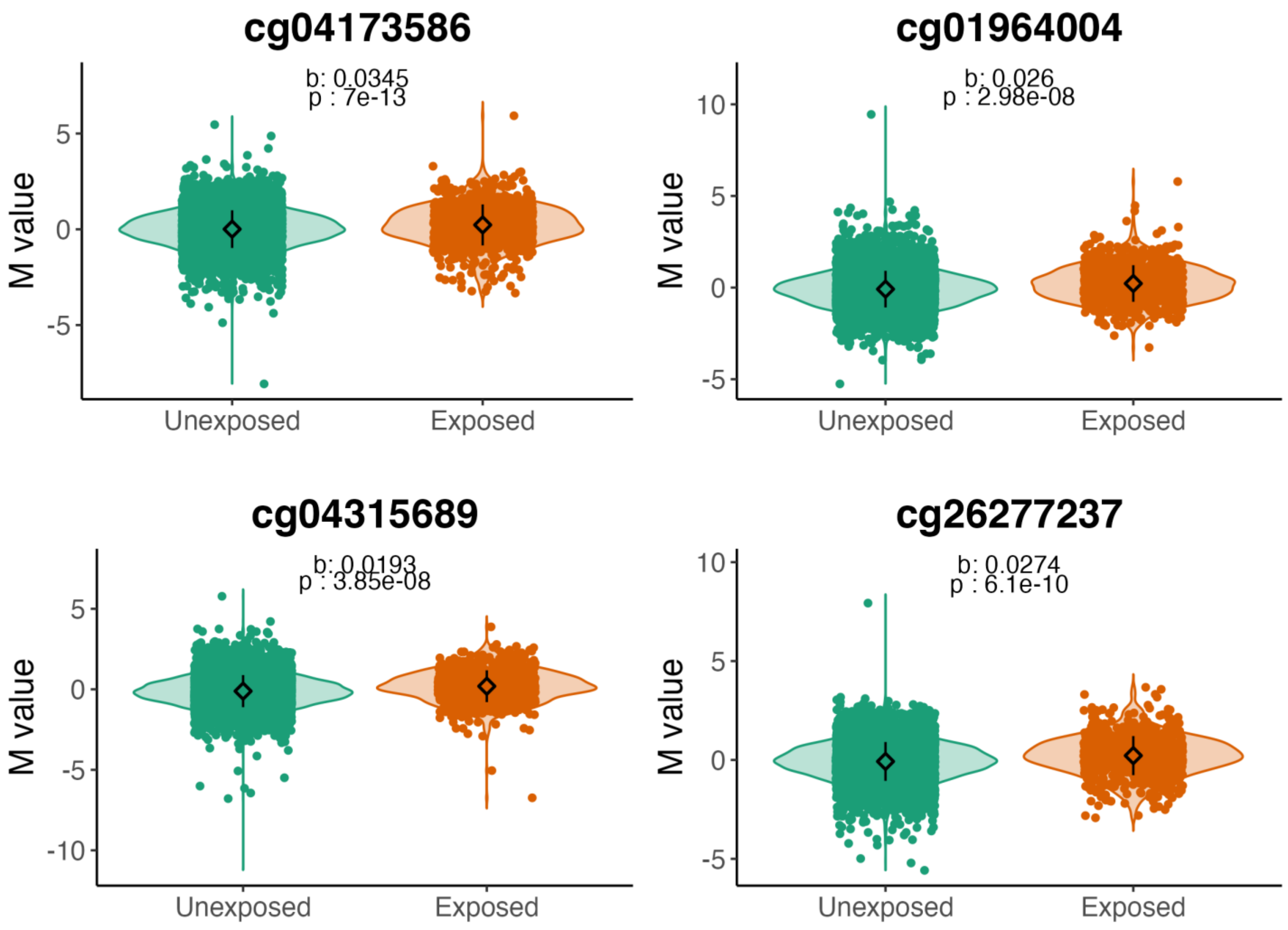

Supplementary Fig. 2: Distribution of M-values for CpGs significantly associated with prescription-derived antidepressant exposure.

Violin plots of the methylation M-values for significant probes ($p < 9.42 \times 10^{-08}$) in the methylome-wide association study (MWAS) of prescription-derived antidepressant exposure. Plots are grouped per probe by antidepressant exposure ($n_{exposed}$ = 861, $n_{unexposed}$=7,090). Overlaid box plots indicate the mean M-value +- the standard deviation. b = Beta calculated in the prescription-derived MWAS, p = p value calculated from the prescription-derived MWAS, n = 7,951 participants).

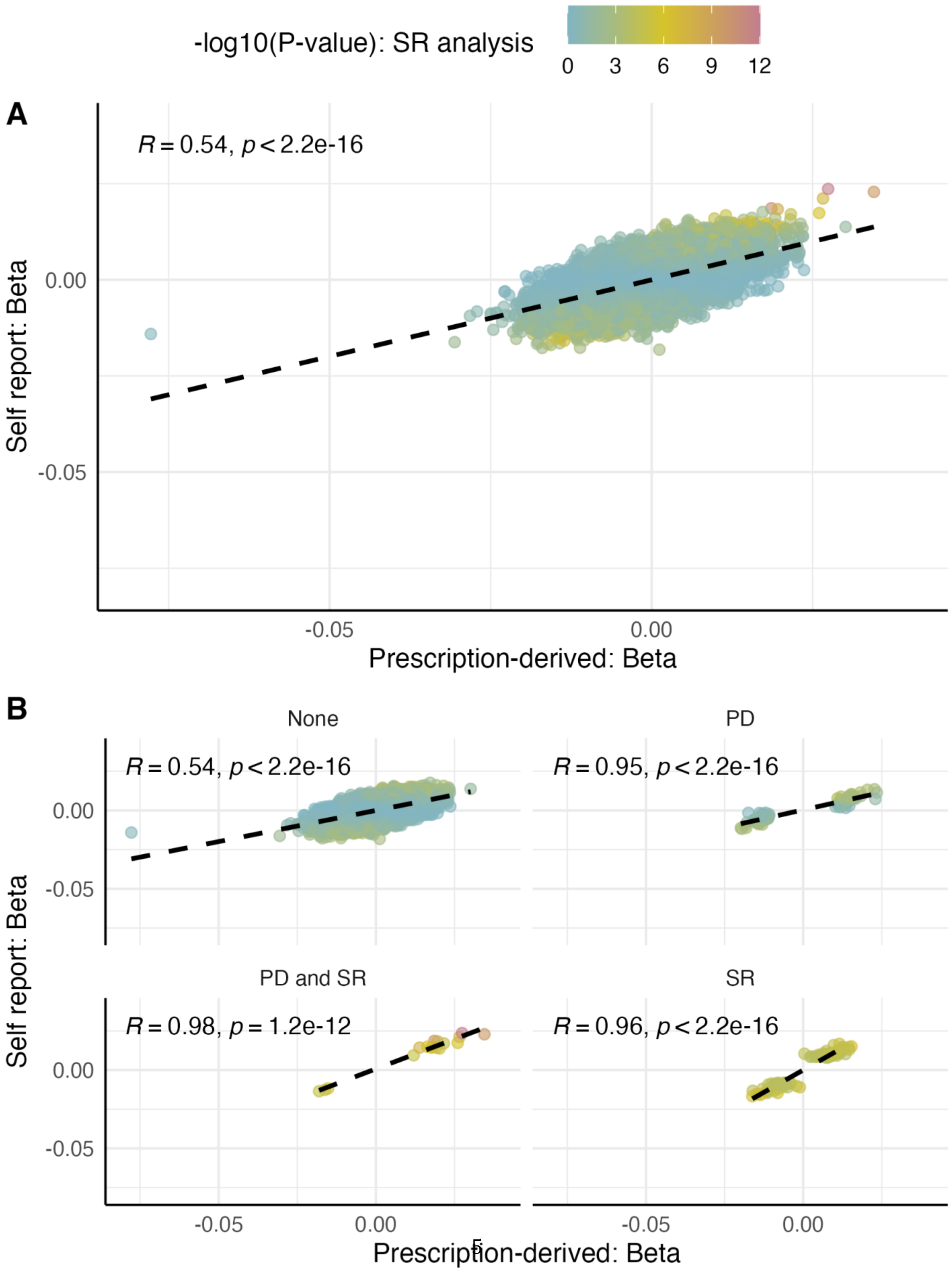

-log10(P-value): SR analysis
0
3
6
9
12
A
R = 0.54, p < 2.2e-16
Self report: Beta
0.00
-0.05
-0.05
0.00
Prescription-derived: Beta
B
None
PD
R = 0.54, p < 2.2e-16
R = 0.95, p < 2.2e-16
PD and SR
SR
R = 0.98, p = 1.2e-12
R = 0.96, p < 2.2e-16

Supplementary Fig. 3: Correlation of CpG effect estimates from the prescription-derived and self-report methylome-wide association studies.

Beta effect estimates for CpGs from the self-report (SR, n = 16,531 participants) and the prescription-derived (PD, n = 7,951 participants) methylome-wide association studies (MWAS). A) All CpG probes effect sizes, colored by the -log(p-value) in the SR MWAS. B) Subplots of the probes stratified into whether probes are present in the top 100 CpGs (ordered by p-value) in both analyses (bottom left), SR MWAS only (top right), PD MWAS only (bottom right), or in neither analysis (Top left). All plots have a regression line, Pearson correlation coefficient and corresponding P value, calculated using the stat_cor function in ggpubr R package[1].

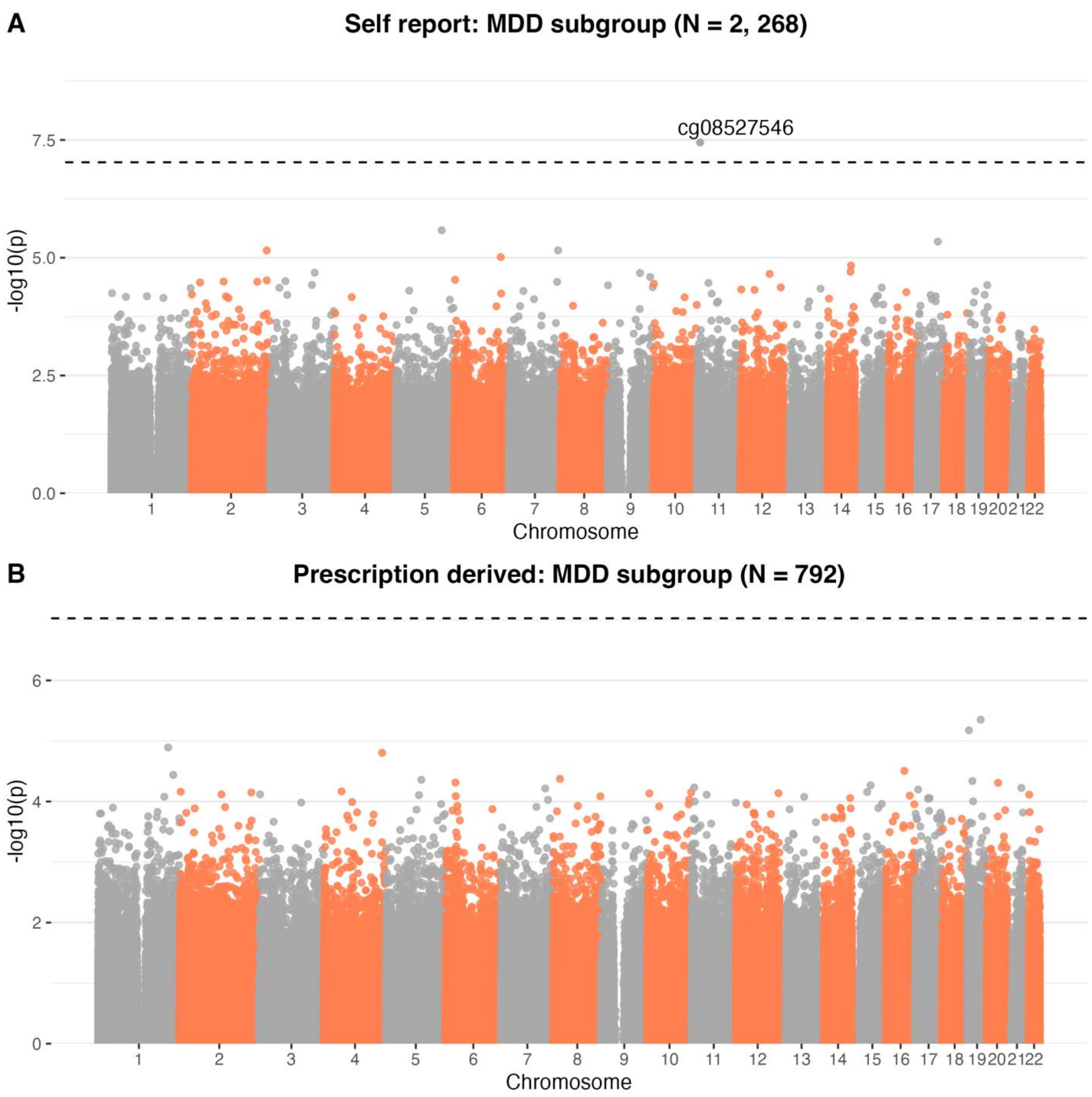

Supplementary Fig. 4: Methylome-wide association studies of self-report and prescription-derived antidepressant exposure in those with lifetime Major Depressive Disorder.

Manhattan plots of the methylome-wide association studies (MWAS) of the A) self-report Major Depressive Disorder (MDD) subgroup (n = 2,268 participants) and B) prescription-derived Major Depressive Disorder (MDD)-subgroup (n = 792 participants). The dotted line represents our methylome-wide threshold for significance, 9.42×10^{-08}, as recommended for case-control MWAS[2]. The MDD-subgroup refers participants with a lifetime MDD status ascertained from the Structured Clinical Interview for DSM-IV Non-Patient Version.

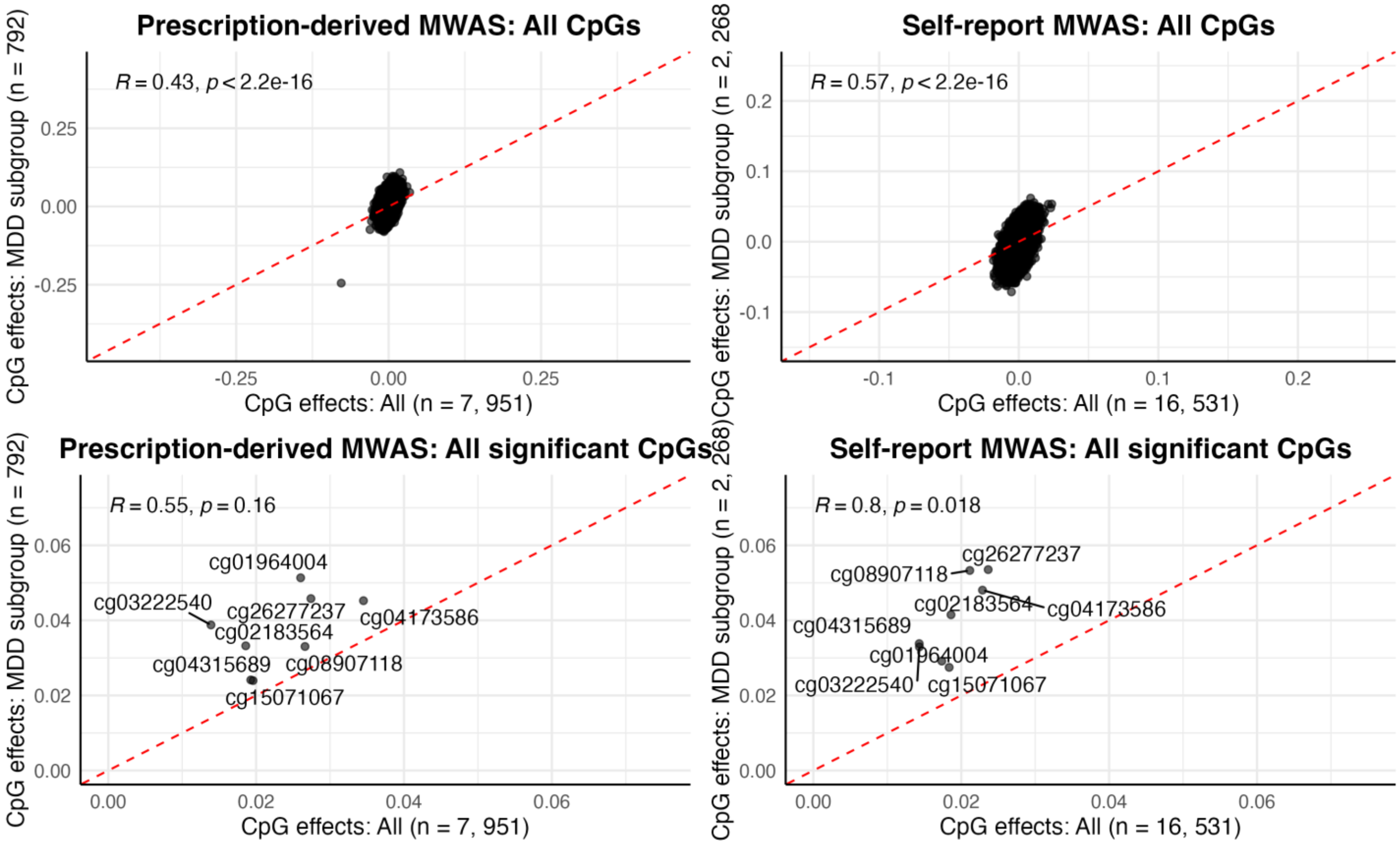

Supplementary Fig. 5: Correlation of CpG effect estimates from the prescription-derived and self-report methylome-wide association studies in all participants and those in the Major Depressive Disorder subgroup.

The correlation of all probe effect sizes for the prescription-derived MWAS (left, n = 7,951 participants) and self-report (right, n = 16,531 participants) MWAS conducted on all individuals (x-axis; All) and on a subset of individuals with lifetime Major Depressive Disorder (MDD) status (y-axis; MDD-subgroup). The bottom panel shows only the CpGs with a significant association in any self-report/prescription derived MWAS performed. All plots have a red-dotted line with equation y=x, and the Pearson correlation coefficient and corresponding P value, calculated using the stat_cor function in ggpubr R package[1].

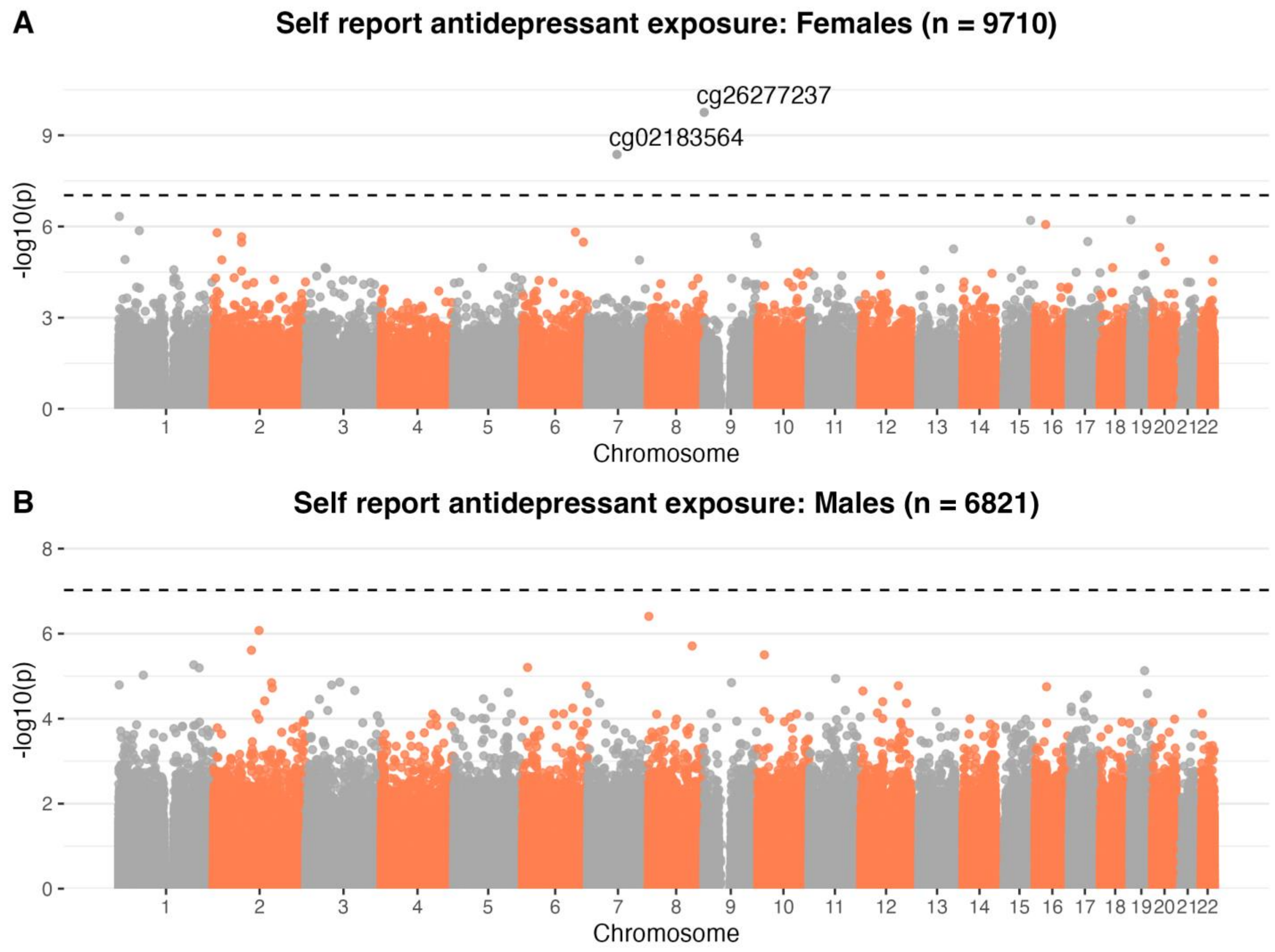

Supplementary Fig. 6: Sex-stratified methylome-wide association studies of self-report antidepressant exposure.

Manhattan plots of the sex-stratified methylome-wide association studies (MWAS) of the self-report antidepressant exposure. Top: females only (n = 9170 participants), Bottom: Males only (n = 6821 participants). The dotted line represents our methylome-wide threshold for significance, 9.42×10^{-08}, as recommended for case-control MWAS[2].

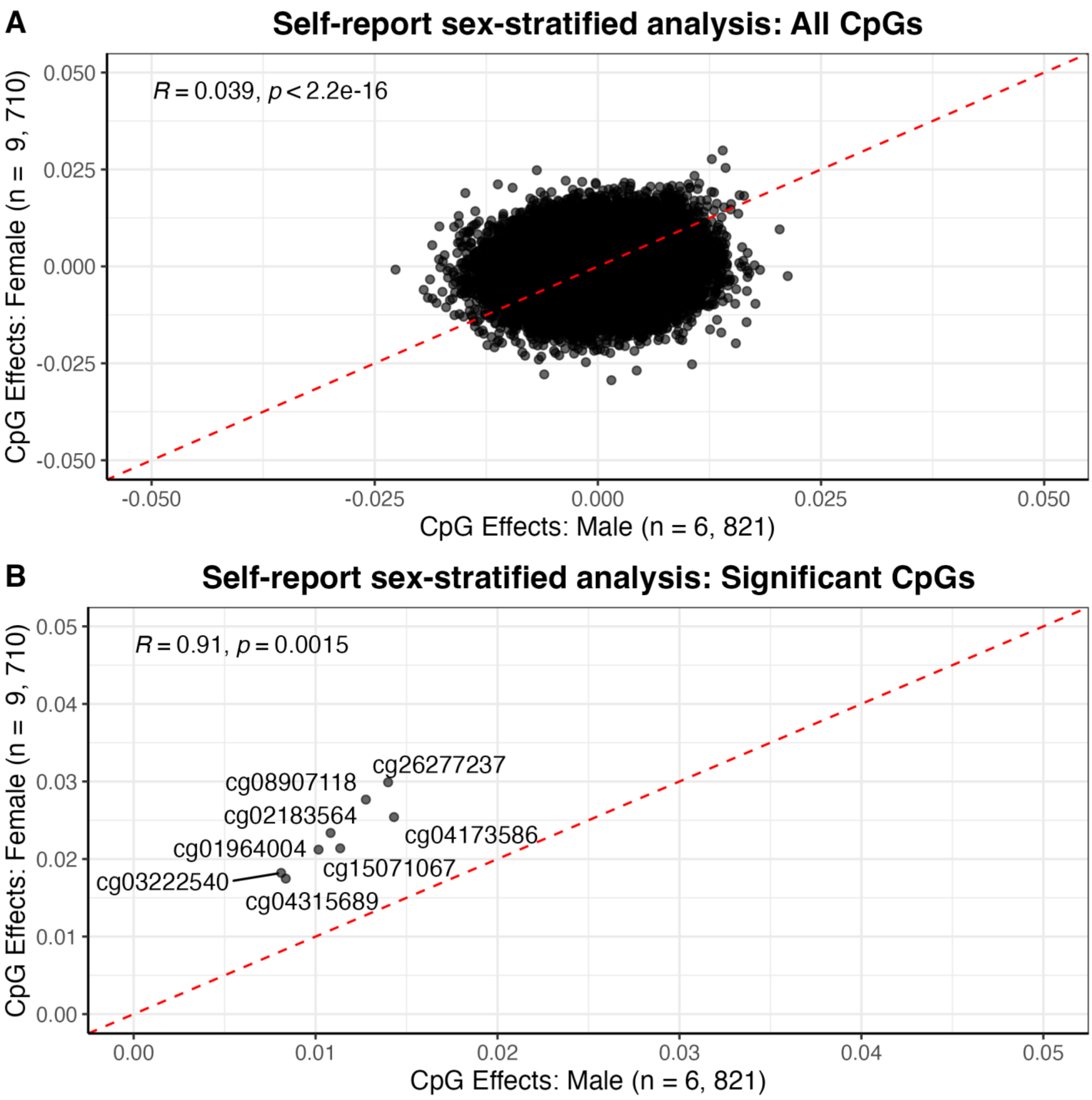

Supplementary Fig. 7: Correlation of effect estimates from the female-only and male-only methylome-wide association studies.

The correlation of all probe effect sizes for sex-stratified self-report antidepressant exposure MWAS analysis (Male: x-axis, Female: y-axis). The top panel shows the effect estimates of all CpGs. The bottom panel shows only the CpGs with a significant association in any non-stratified self-report/prescription derived MWAS performed (MWAS $p < 9.42x10^{-08}$). Each plot shows the Pearson correlation coefficient and corresponding P

value, calculated using the stat_cor function in ggpubr R package[1]. Female MWAS: n = 9,710 participants, Male MWAS: n = 6,821 participants.

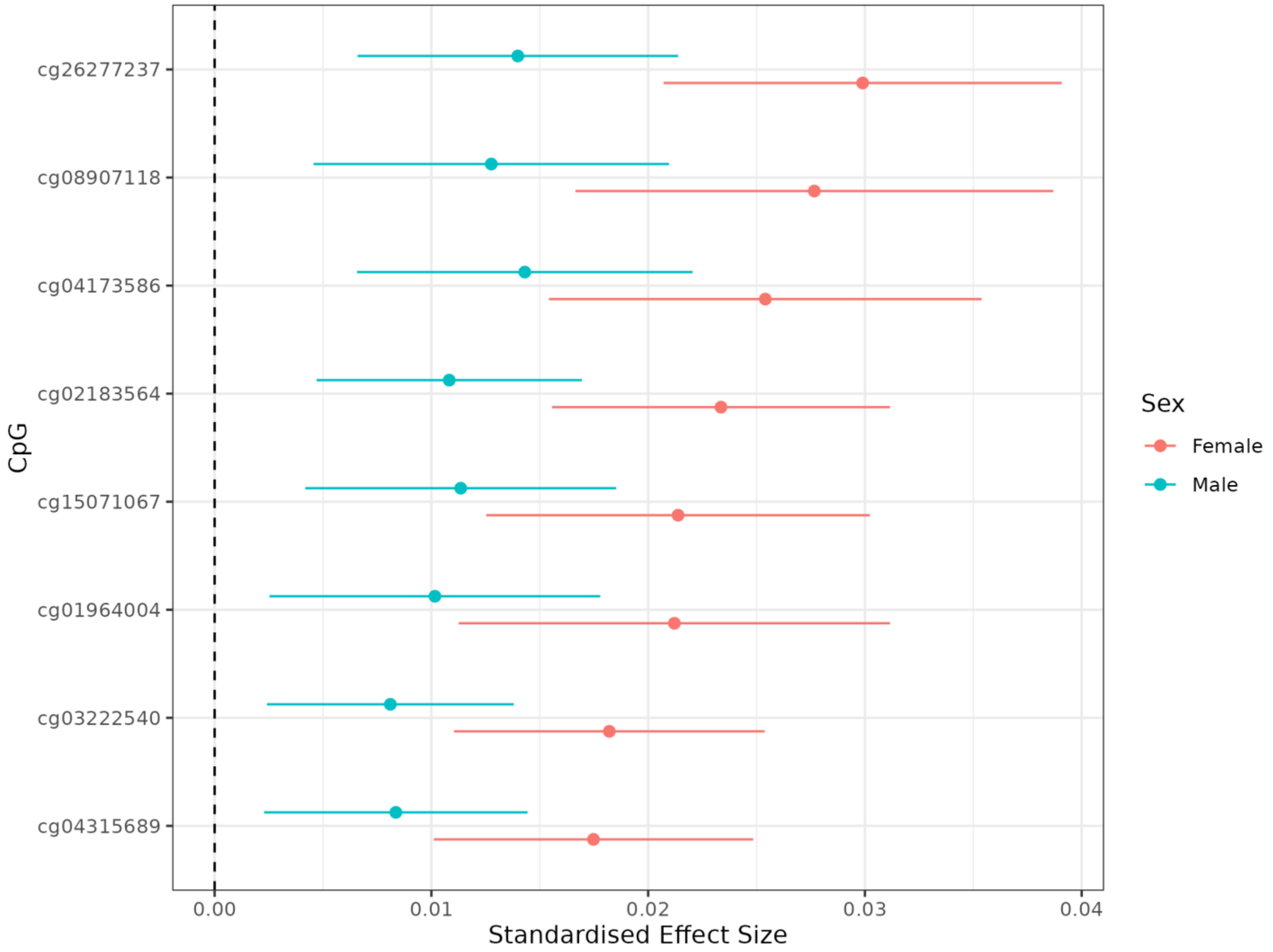

Supplementary Fig. 8: The effect estimates from the sex-stratified methylome-wide association studies for CpGs significantly associated with antidepressant exposure in the non-stratified analysis.

Effect estimates (central dot) and 95% confidence intervals (bars) of CpGs from sex-stratified methylome-wide association studies (MWAS) of self-report antidepressant exposure. The CpGs displayed are the eight which were significantly associated (MWAS $p < 9.42x10^{-08}$) with self-report/prescription-derived antidepressant exposure in non-stratified analyses. Female MWAS: n = 9,710 participants, Male MWAS: n = 6,821 participants.

Differentially methylated regions

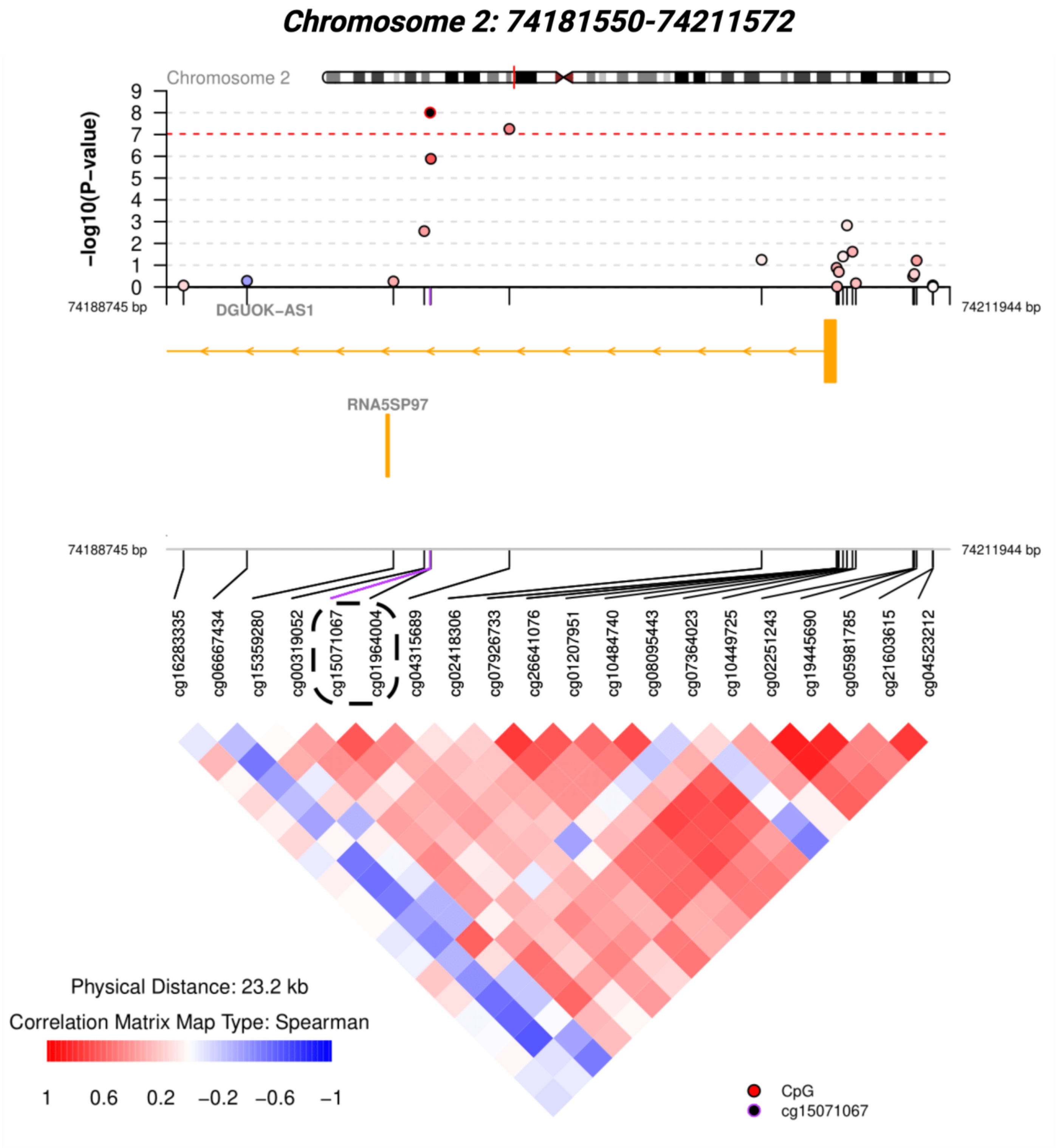

Supplementary Fig. 9: A significant differentially methylated region identified in the methylome-wide association study of self-reported antidepressant exposure.

The significant differentially methylated region (DMR; Beta = 0.096, p_{adj} = 4.98x10-3) from the self-report MWAS (n = 16,531 participants) calculated using the dmrff R package[3]. The top panel presents the CpGs within and near the DMR, coloured by their correlation with cg15071067. The orange bars show gene-tracks, and the bottom heatmap presents the spearman correlation matrix of the CpGs. Plot was created using the coMET R package[4].

Overlap of PD and SR CpGs and their annotated gene lists

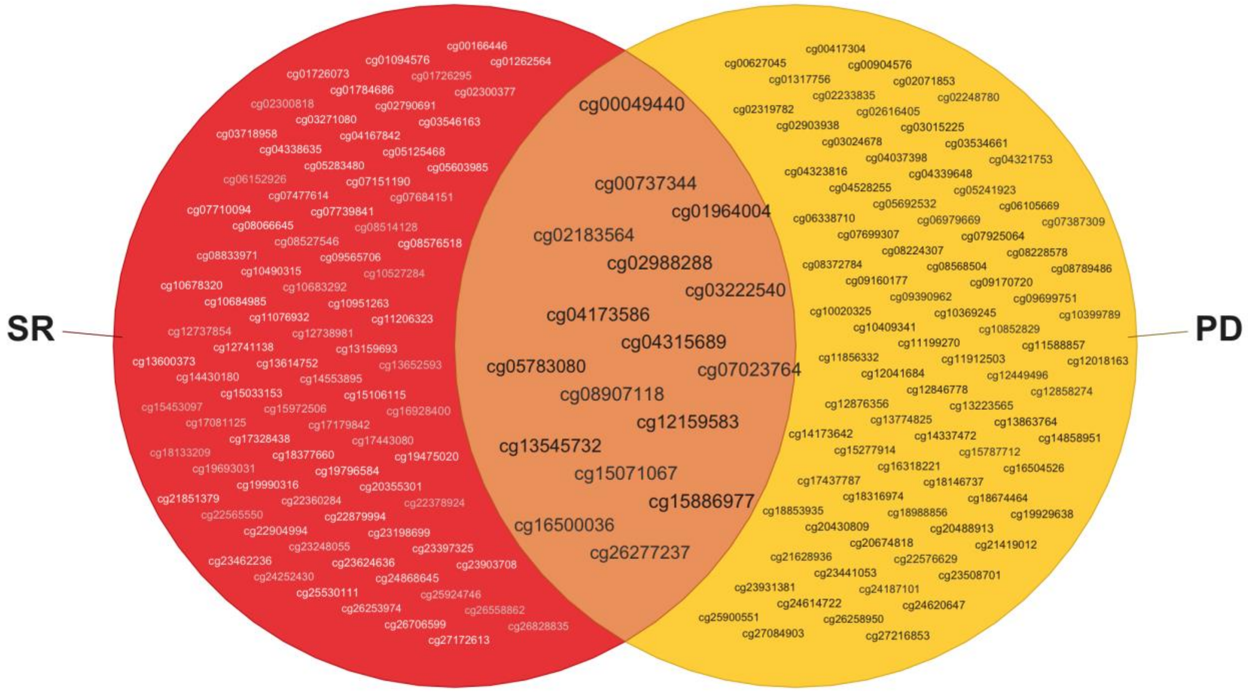

Supplementary Fig. 10: Overlap of the top 100 CpGs from the self-report and prescription-derived methylome-wide association studies.

Top 100 CpGs from the self-report MWAS (red circle, n = 16,531 participants) and the prescription-derived MWAS (yellow circle, n = 7,951 participants). There are 17 overlapping CpGs (orange circle) from each CpG list. Hypergeometric tests, implemented using the phyper() function indicate that this overlap is significantly more than expected by chance ($p = 1.95 \times 10^{-48}$). The background CpG set was all genes which could be annotated to the Illumina EPIC array following quality control filters in Generation Scotland (n = 752,741).

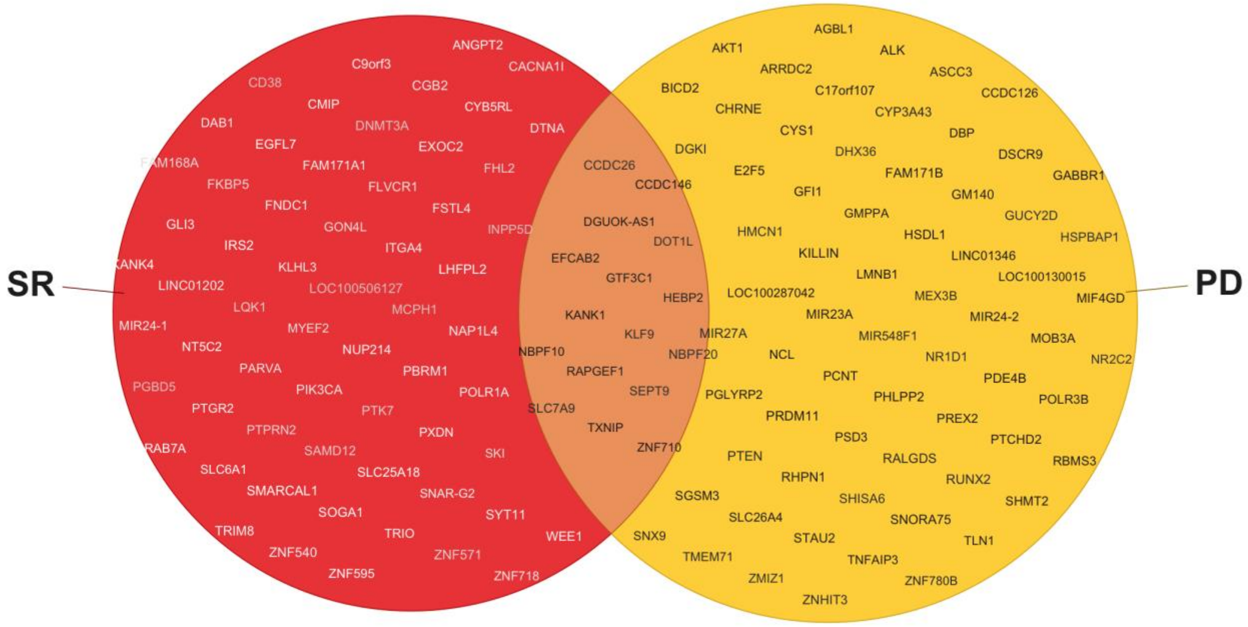

Supplementary Fig. 11: Overlap of the top gene-sets from the self-report and prescription-derived methylome-wide association studies.

The genes which map to the top 100 CpGs from the self-report MWAS (red circle, n = 77) and the prescription-derived MWAS (yellow circle, n = 83). There were 16 overlapping genes (orange circle) from each gene list. Hypergeometric tests, implemented using the phyper() function indicate that this overlap is significantly more than expected by chance ($p = 1.3x10^{-25}$). The background gene set was all genes which could be annotated to the Illumina EPIC array following quality control filters in Generation Scotland. Genes annotated CpGs were derived using the Infinium MethylationEPIC BeadChip database[5] (n = 27,365 genes).

Tissue Specificity

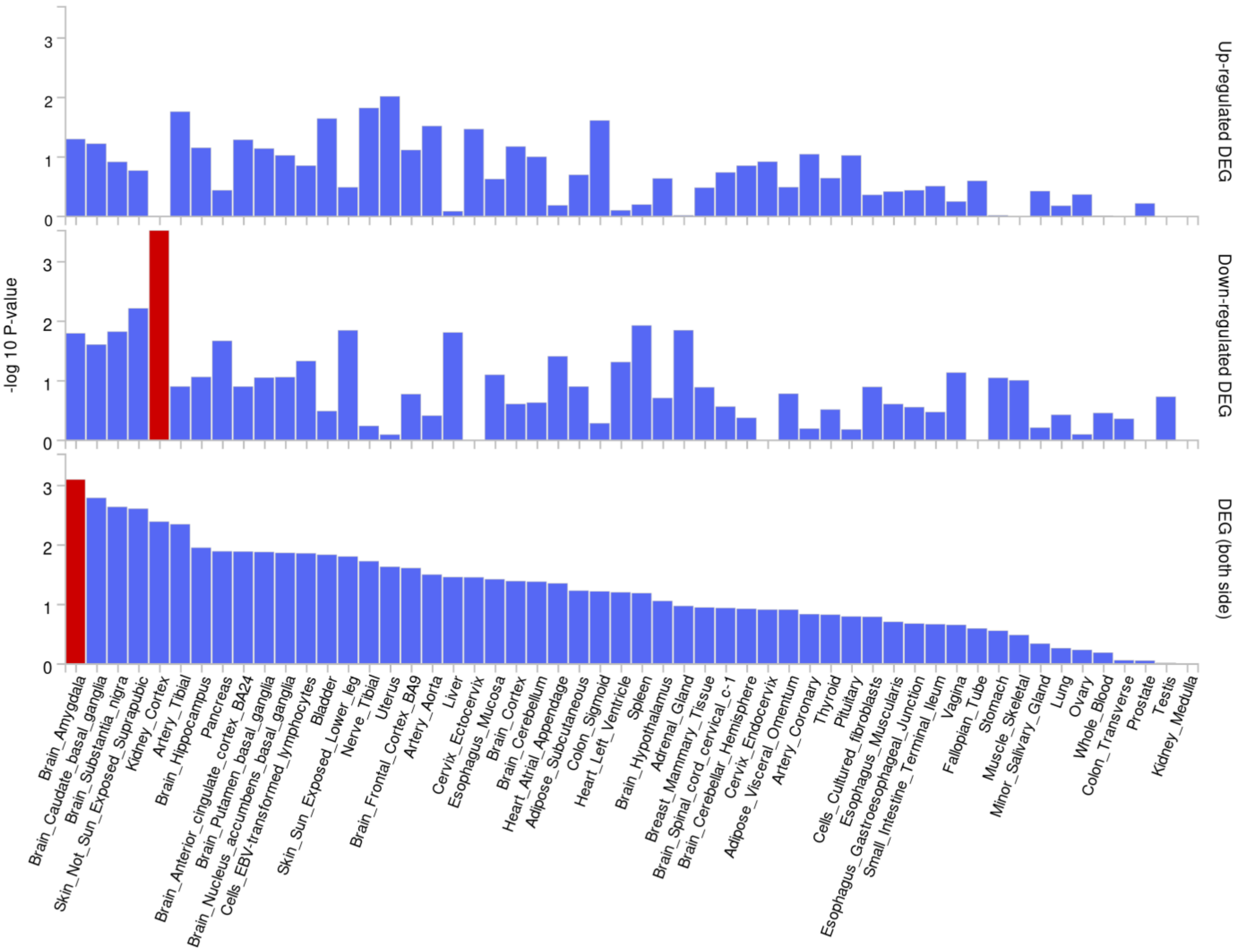

Supplementary Fig. 12: Tissue enrichment results for the top gene-set from the self-report methylome-wide association study.

Enrichment of the self-report gene set (n = 77 genes, derived from the self-report MWAS on 16,531 participants) and 54 specific tissues from GTEx v8, assessed using a hypergeometic test, returned from the GENE2FUNC tool in FUMA[6]. Enrichment results are displayed for genes up-regulated (top), down-regulated (middle) or both (bottom) in each tissue. The background gene set used in all enrichment tests was all genes which could be annotated to the Illumina EPIC array using the Infinium MethylationEPIC BeadChip database[5] following quality control filters in Generation Scotland (n = 27,365 genes).

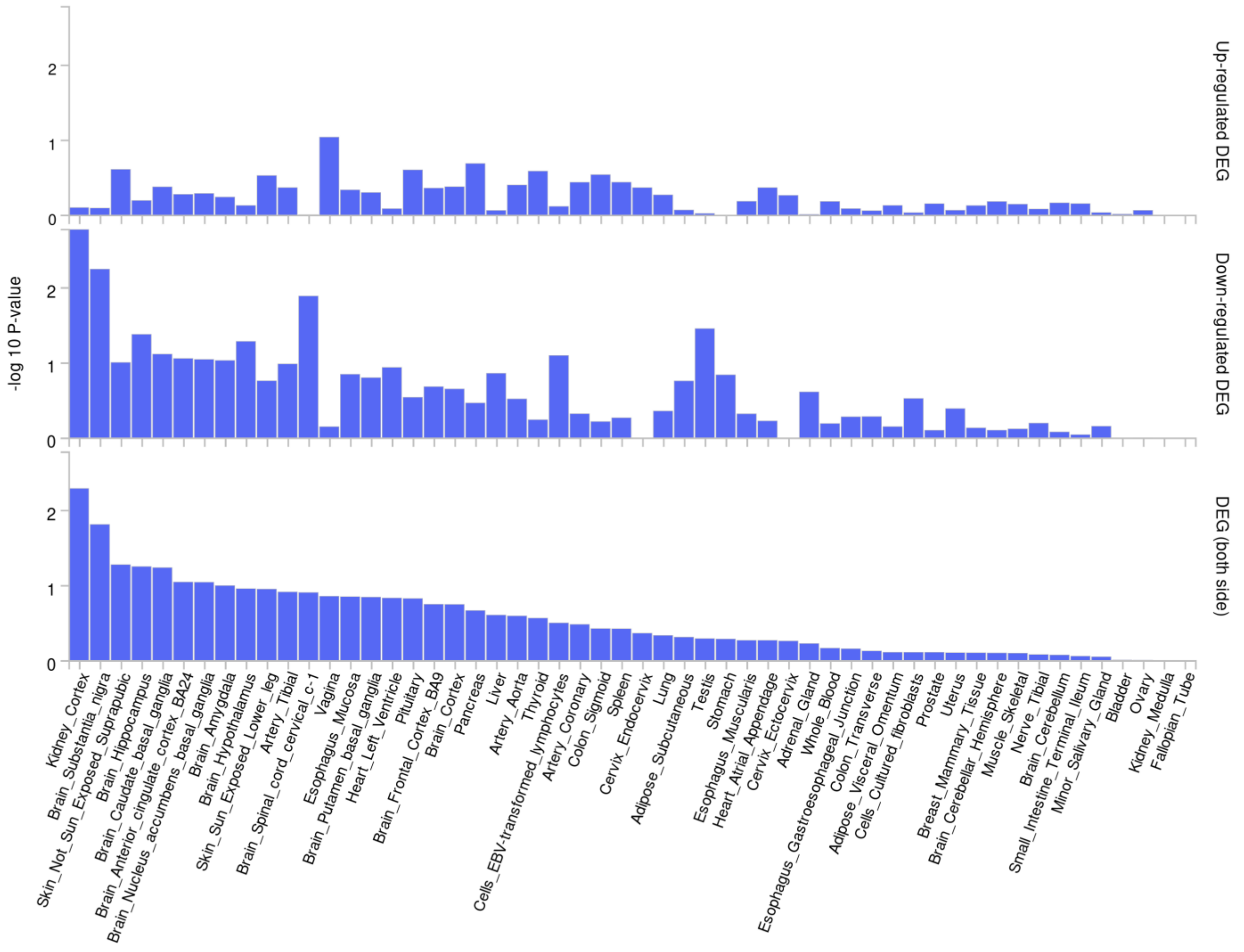

Supplementary Fig. 13: Tissue enrichment results for the top gene-set from the prescription-derived methylome-wide association study.

Enrichment of the prescription-derived gene set (n = 83 genes, derived from the prescription-derived MWAS on 7,951 participants) and 54 specific tissues from GTEx v8 assessed using a hypergeometric test, returned from the GENE2FUNC tool in FUMA[6]. Enrichment results are displayed for genes up-regulated (top), down-regulated (middle) or both (bottom) in each tissue. The background gene set used in all enrichment tests was all genes which could be annotated to the Illumina EPIC array using the Infinium MethylationEPIC BeadChip database[5] following quality control filters in Generation Scotland (n = 27,365 genes).

Gene Ontology analysis

SynGo

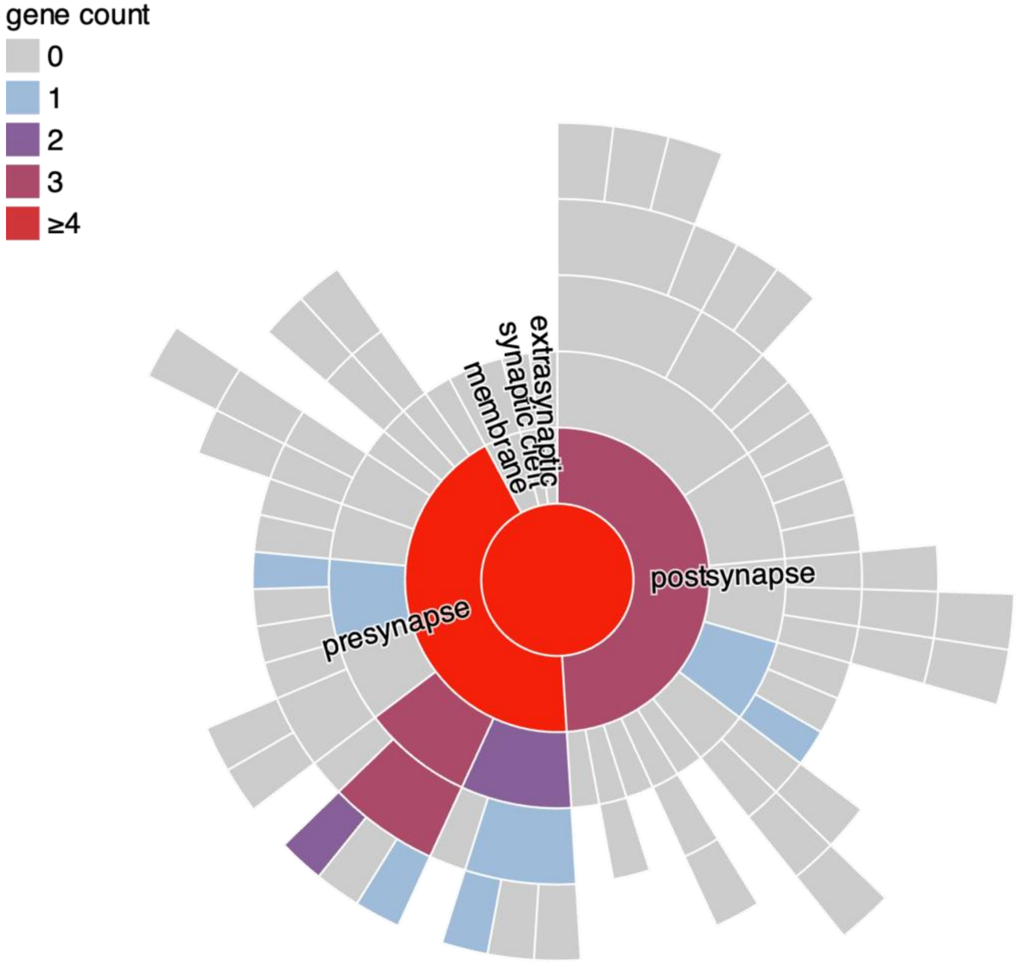

Supplementary Fig. 14: SynGO terms and the number of genes present in the top gene-set from the self-report methylome-wide association study.

All SynGO terms and their overlap with genes in the self-report gene set (n = 77 genes, derived from the self-report MWAS in 16,531 participants), separated by presynaptic (left) or post synaptic (right) location (returned

from SynGO web portal)[7]. Each SynGO ontology is colored by how many genes in that ontology are present within the self-report gene list.

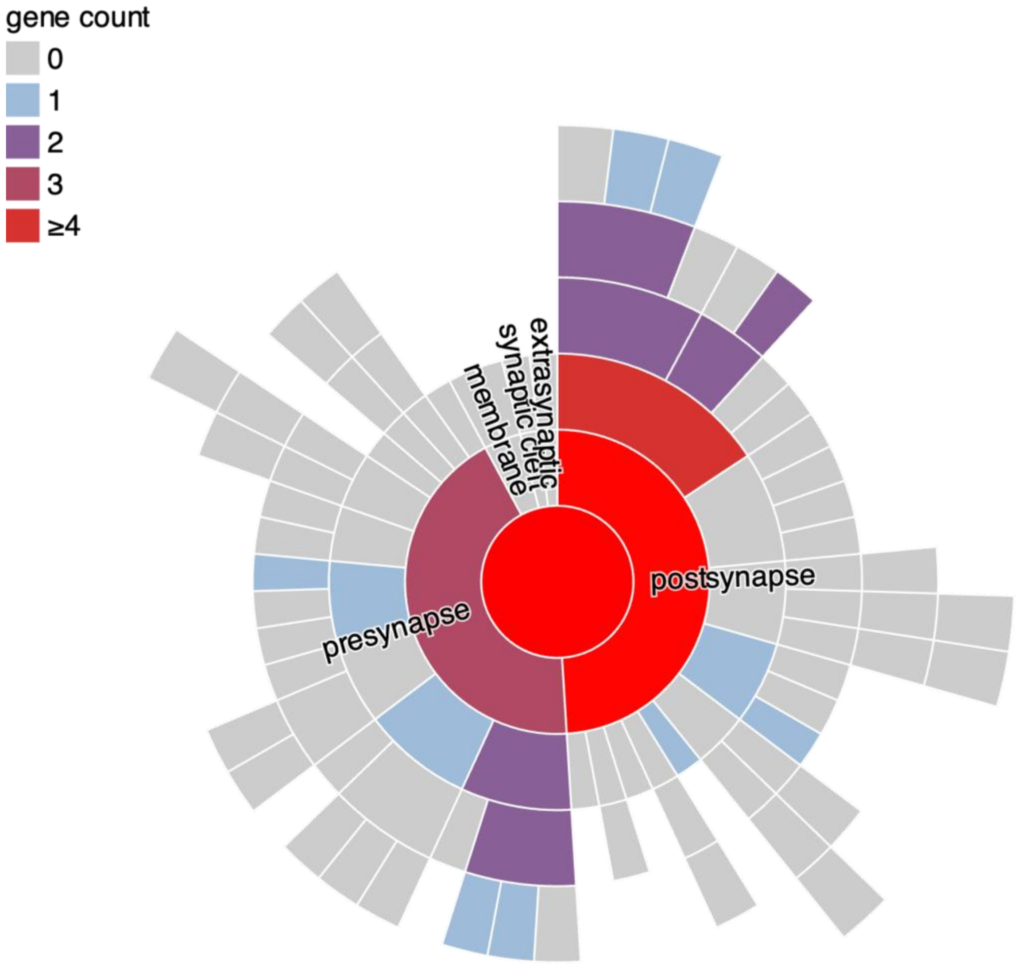

Supplementary Fig. 15: SynGO terms and the number of genes present in the top gene-set from the prescription-derived methylome-wide association study.

All SynGO terms and their overlap with genes in the prescription-derived gene set (n = 83 genes, derived from the prescription-derived MWAS in 7,951 participants), separated by presynaptic (left) or post synaptic (right) location (returned from SynGO web portal)[7]. Each SynGO ontology is colored by how many genes in that ontology are present within the prescription derived gene list.

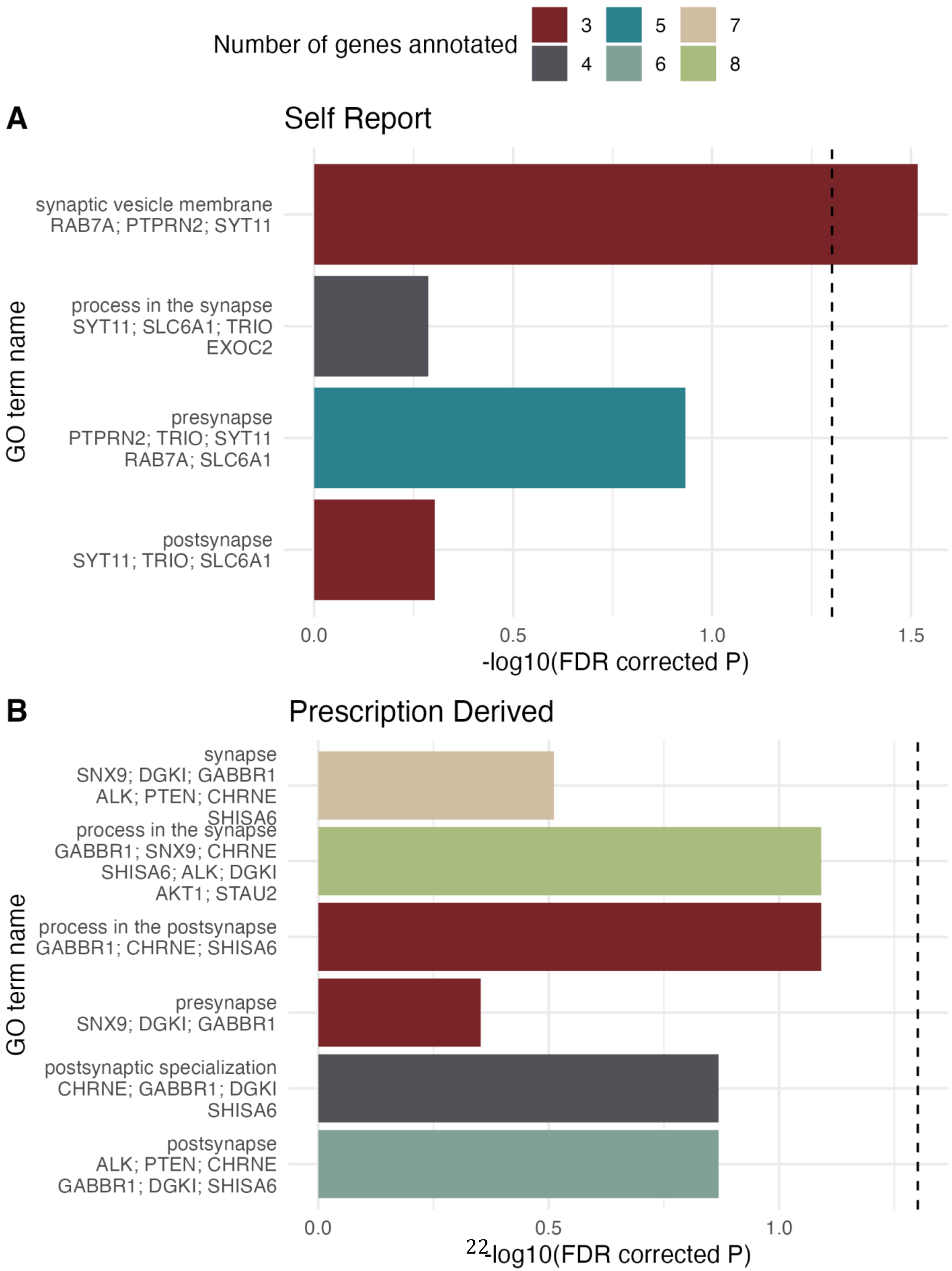

Number of genes annotated
3
4
5
6
7
8
A
Self Report
GO term name
synaptic vesicle membrane
RAB7A; PTPRN2; SYT11
process in the synapse
SYT11; SLC6A1; TRIO
EXOC2
presynapse
PTPRN2; TRIO; SYT11
RAB7A; SLC6A1
postsynapse
SYT11; TRIO; SLC6A1
0.0
0.5
1.0
1.5
-log10(FDR corrected P)
B
Prescription Derived
GO term name
synapse
SNX9; DGKI; GABBR1
ALK; PTEN; CHRNE
SHISA6
process in the synapse
GABBR1; SNX9; CHRNE
SHISA6; ALK; DGKI
AKT1; STAU2
process in the postsynapse
GABBR1; CHRNE; SHISA6
presynapse
SNX9; DGKI; GABBR1
postsynaptic specialization
CHRNE; GABBR1; DGKI
SHISA6
postsynapse
ALK; PTEN; CHRNE
GABBR1; DGKI; SHISA6
0.0
0.5
1.0
-log10(FDR corrected P)

Supplementary Fig. 16: Enrichment of the top gene-sets from the self-report and prescription-derived methylome-wide association studies with SynGO ontologies.

Enrichment of the self-report gene-set (top; 77 genes, 16,531 participants) and prescription-derived gene-set (bottom; 83 genes, 7,951 participants) with the SynGO ontology terms. Enrichment was assessed using hypergeometric tests in the SynGO web portal[7]. For both groups, SynGO terms are displayed on the y-axis, and the -log(P-value$_{FDR\text{-}Corrected}$) of the enrichment test on the x-axis. Each SynGO term is annotated with the overlapping genes from the ontology and the MWAS gene-set, while the bar colors represent the number of these overlapping genes. The dotted line represents the threshold for significance ($P_{FDR} < 0.05$). The only term with significant enrichment was the synaptic vesicle membrane with the self-report gene set ($p_{FDR}=0.030$).

Msigdbr GO: Biological Processes

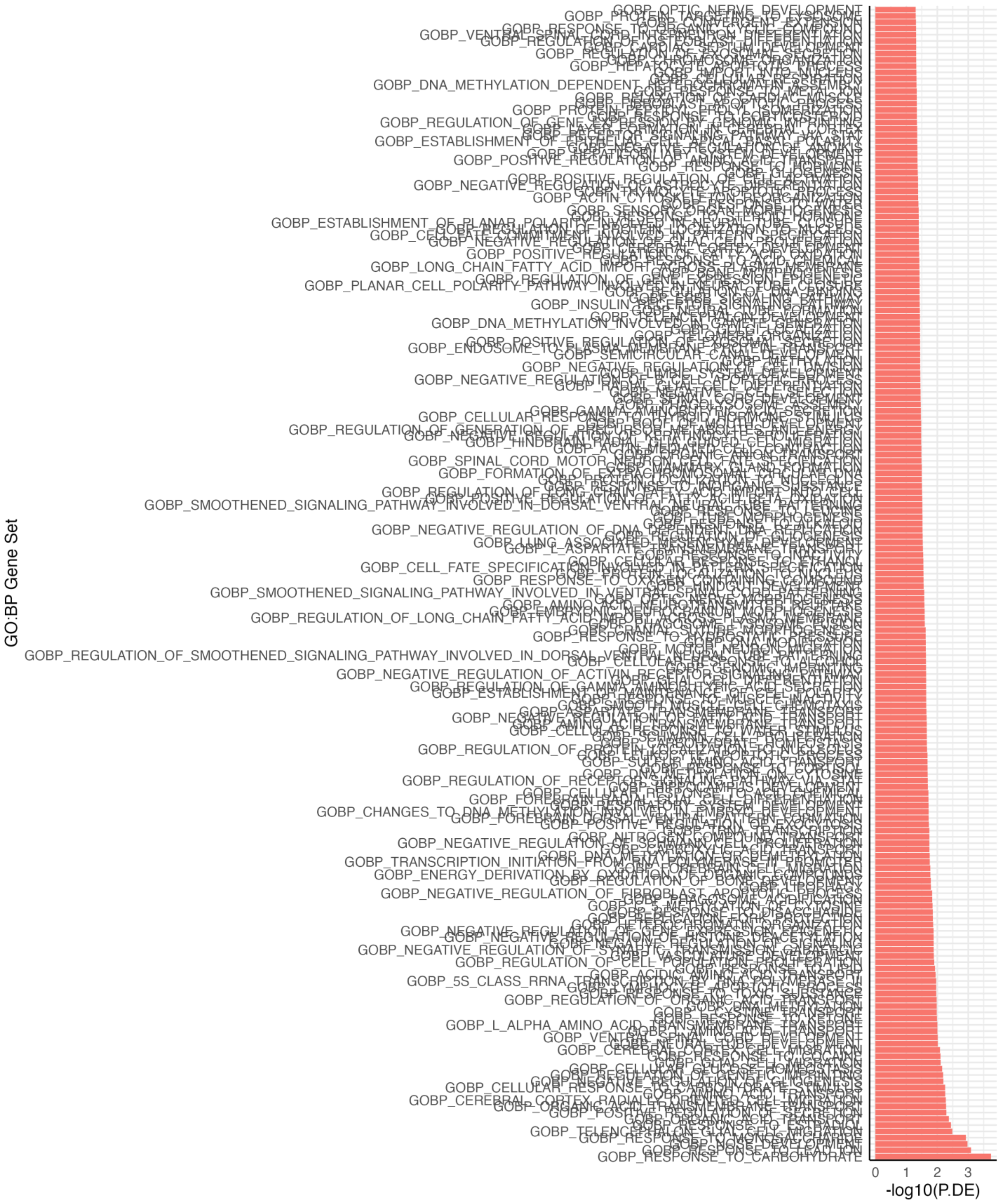

Supplementary Fig. 17: Enrichment of the top gene-set from the self-report methylome-wide association study with GO:Biological Process ontologies.

GO: Biological Processes gene sets which were nominally enriched for the self-report gene set (n = 77 genes, 16,531 participants). Enrichment was assessed using hypergeometric tests, specifically implementing the 'gsameth()' function from 'missMethyl' R package[8] which adjusts for the varying number of CpGs present in each gene. The GO terms are displayed on the y-axis, and the -log(P-value) of the enrichment test on the x-axis. None of the gene-sets were significantly enriched following multiple comparisons corrections. Information about each GO gene set can be found in Supplementary Table 29.

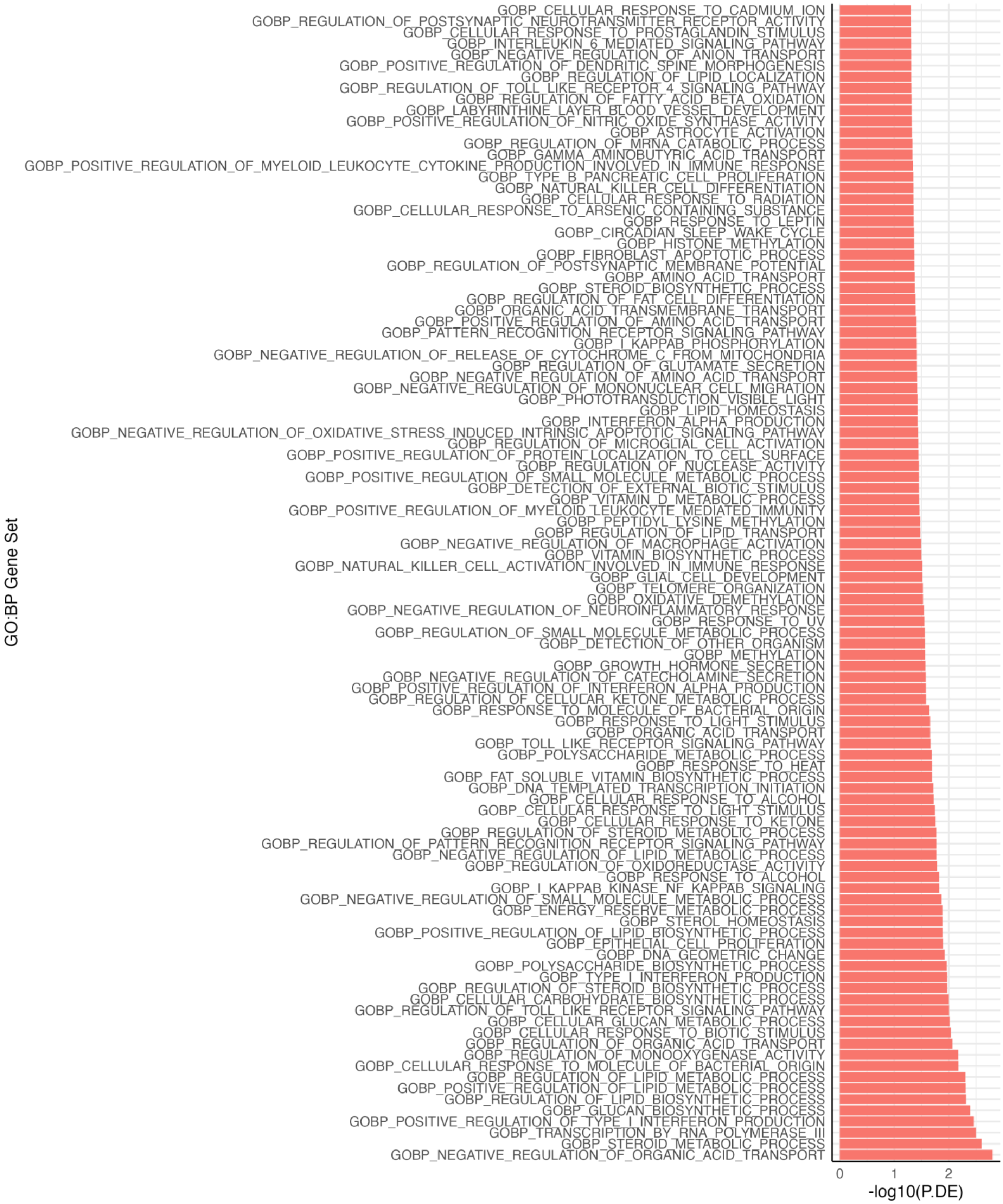

Supplementary Fig. 18: Enrichment of the top gene-set from the prescription-derived methylome-wide association study with GO:Biological Process ontologies.

GO: Biological Processes gene sets which were nominally enriched for the prescription-derived gene set (n = 83 genes, 7,951 participants). Enrichment was assessed using hypergeometric tests, specifically implementing the 'gsameth()' function from 'missMethyl' R package[8] which adjusts for the varying number of CpGs present in each gene. The GO terms are displayed on the y-axis, and the -log(P-value) of the enrichment test on the x-axis. None of the gene-sets were significantly enriched following multiple comparisons corrections. Information about each GO gene set can be found in Supplementary Table 29.

Correlation of methylation and time in treatment periods

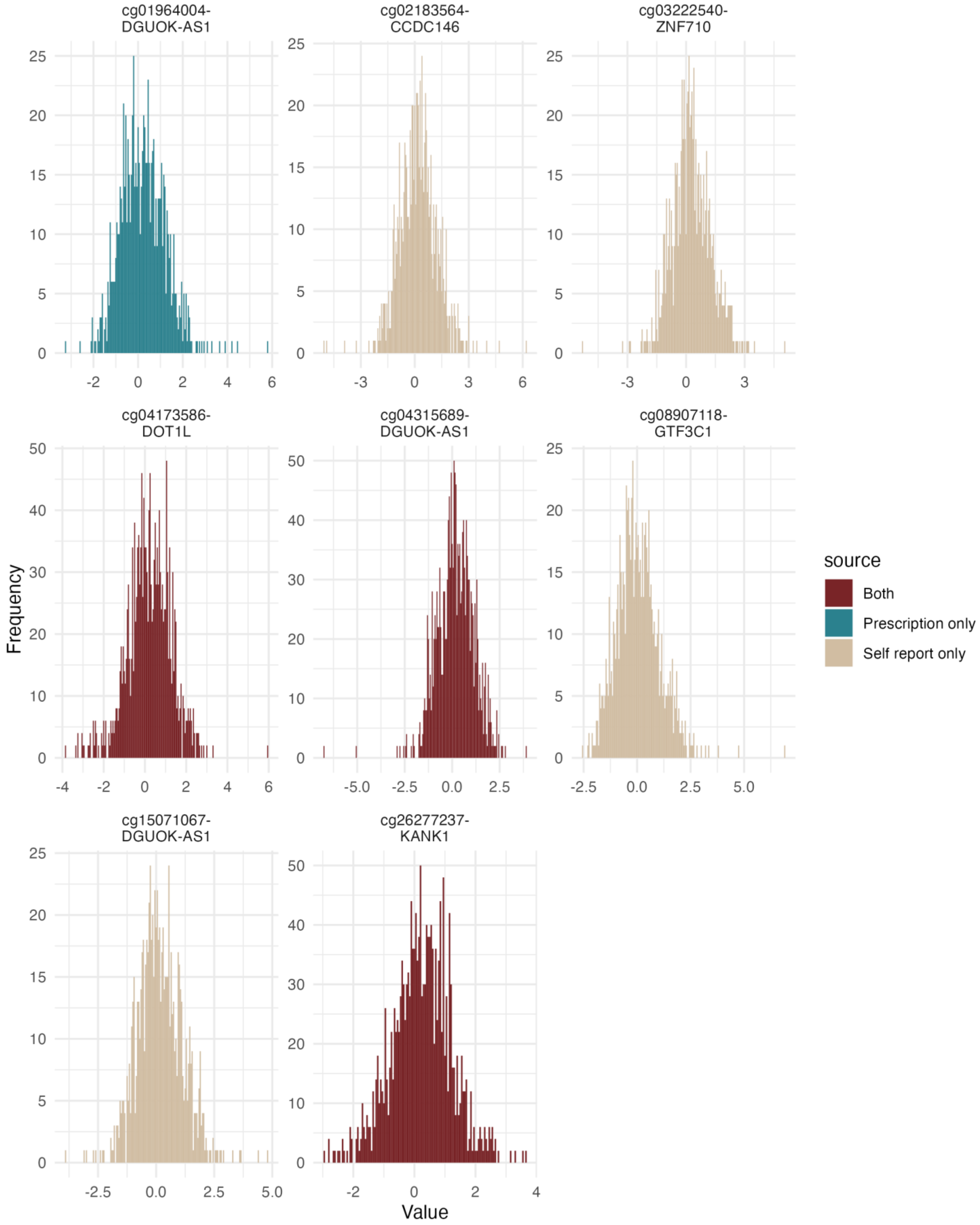
cg01964004-
DGUOK-AS1
cg02183564-
CCDC146
cg03222540-
ZNF710
cg04173586-
DOT1L
cg04315689-
DGUOK-AS1
cg08907118-
GTF3C1
cg15071067-
DGUOK-AS1
cg26277237-
KANK1
Frequency
Value
source
Both
Prescription only
Self report only

Supplementary Fig. 19: Distribution of methylation M-values for probes significantly associated with antidepressant exposure in those within a treatment period.

Histograms of the methylation M-values (x-axis) of probes significantly associated with antidepressant exposure (in either the self-report MWAS and/or prescription-derived MWAS), in those in a treatment period at the time of blood draw (n = 863 participants). Colour of the bars denotes whether the CpG was significant in the self-report MWAS (n = 16,531 participants), prescription-derived MWAS (n = 7,951 participants) or both.

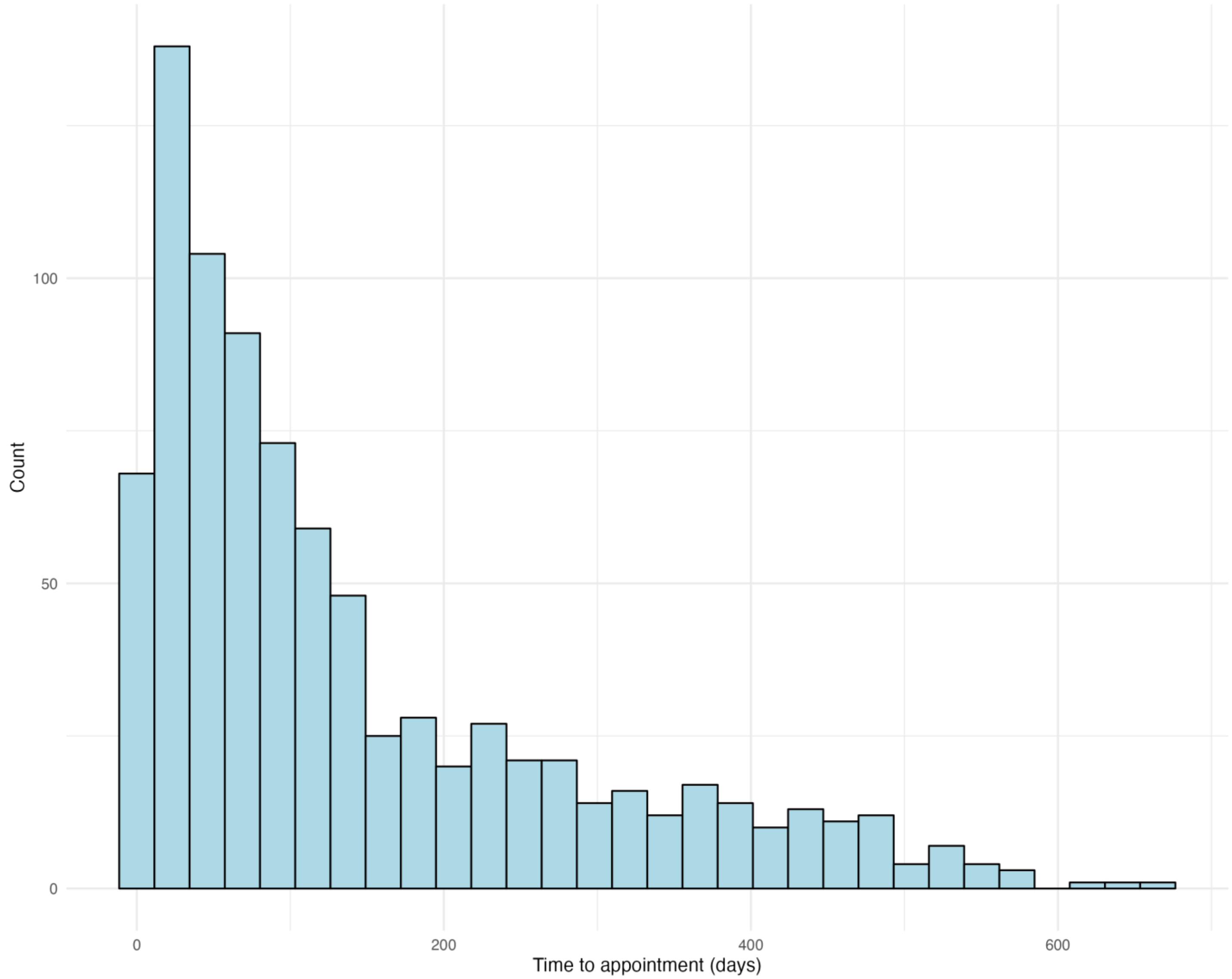

Supplementary Fig. 20: Distribution of the time within a treatment period at the time of DNA methylation measurement.

Histogram of the time between the beginning of a current treatment period and the blood draw appointment (DNAm measurement), for those within a treatment period at the time of the blood draw (N = 863 participants).

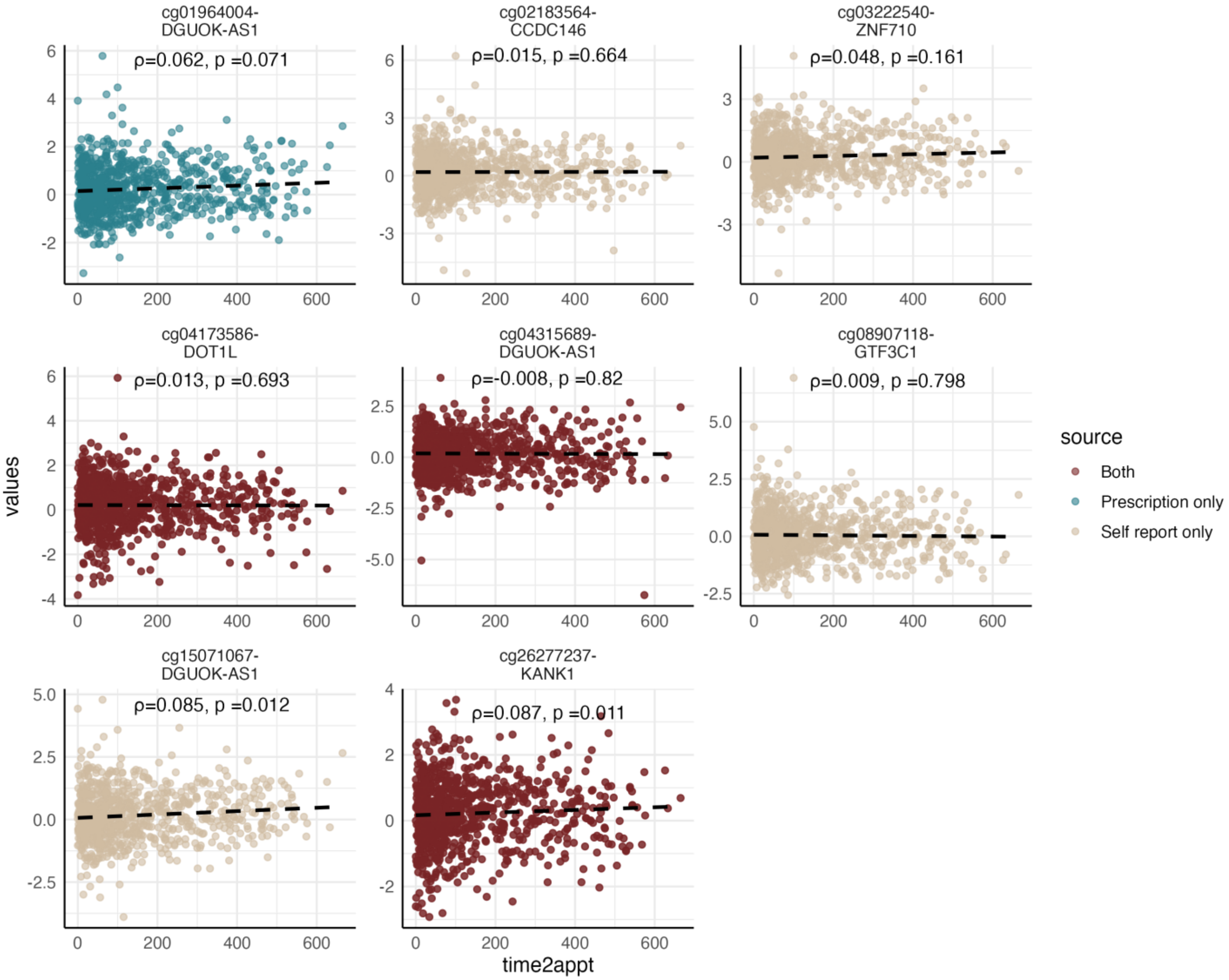

Supplementary Fig. 21: Correlation of methylation levels with the time in a treatment period for CpGs significantly associated with antidepressant exposure.

Methylation at significant probes identified from self-report MWAS (n = 16,531 participants) and prescription-derived AD MWAS (n = 7,951 participants) and the time in treatment prior to the blood draw (n = 863 participants). Regression lines are added to each graph using the geom_smooth(method =lm) function. Two-tailed spearman correlation tests were performed, with the rho and p-values printed on each graph. Colour of the dots denotes whether the CpG was significant in the self-report MWAS, prescription-derived MWAS or both.

Methylation Profile Score

LASSO training model

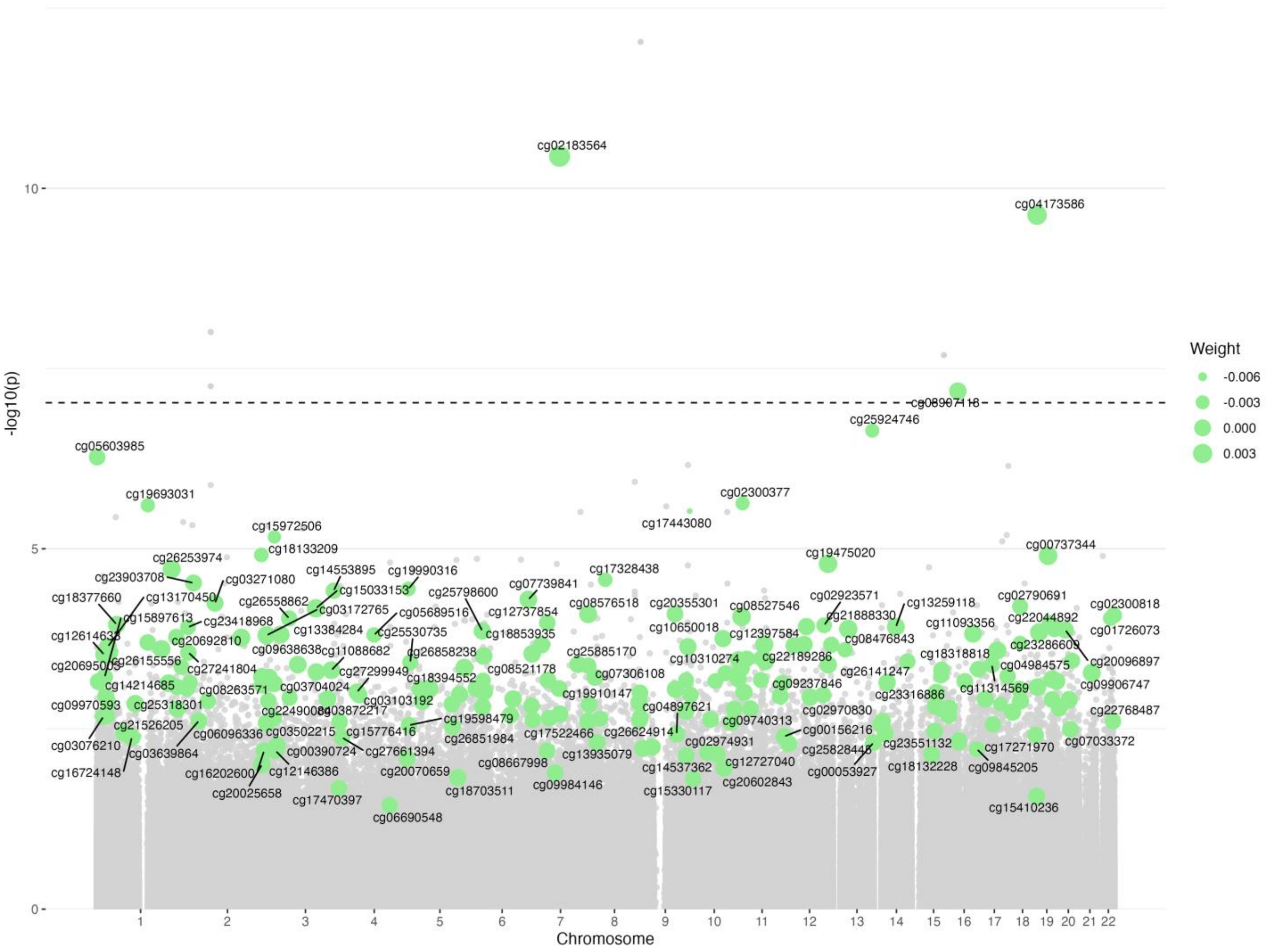

Supplementary Fig. 22: The CpGs used to create a methylation profile score.

Manhattan plot of the self-reported antidepressant use methylome-wide association study (MWAS) (n = 16,531 participants). The gray dashed line denotes the methylome-wide threshold for signifiance, 9.42×10^{-08}, as recommended for case-control MWAS[2]. The 212 CpGs with a non-zero weight from the antidepressant exposure ~ DNAm least absolute shrinkage and selection operator (LASSO) model in Generation Scotland are highlighted in green and labelled. The size of the green dot represents the size of the weight of the CpG in the LASSO model.

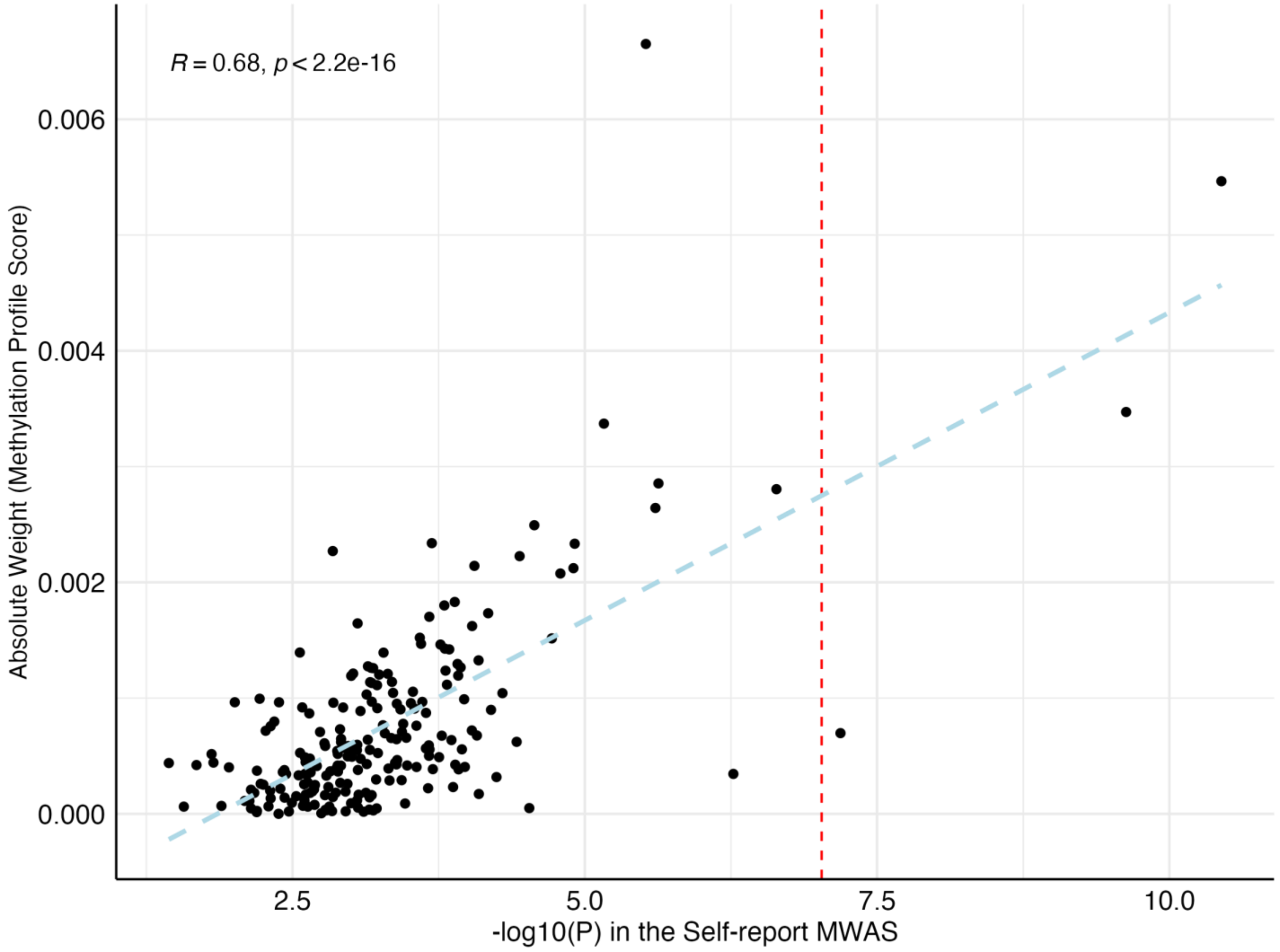

Supplementary Fig. 23: The weight per CpG in the methylation profile score vs the -log(P-value) from the self-report methylome-wide association study.

The -log10(p) from the self-report MWAS (n = 16,531 participants) and the weights assigned to the CpG in the LASSO regression for the 212 CpGs included in the methylation profile score. The red dashed line denotes the methylome-wide threshold for significance, 9.42×10^{-08}, as recommended for case-control MWAS[2]. The light blue dashed line is the regression line between the two variables and the Pearson correlation coefficient and corresponding P value, calculated using the stat_cor function in ggpubr R package[1] is displayed.

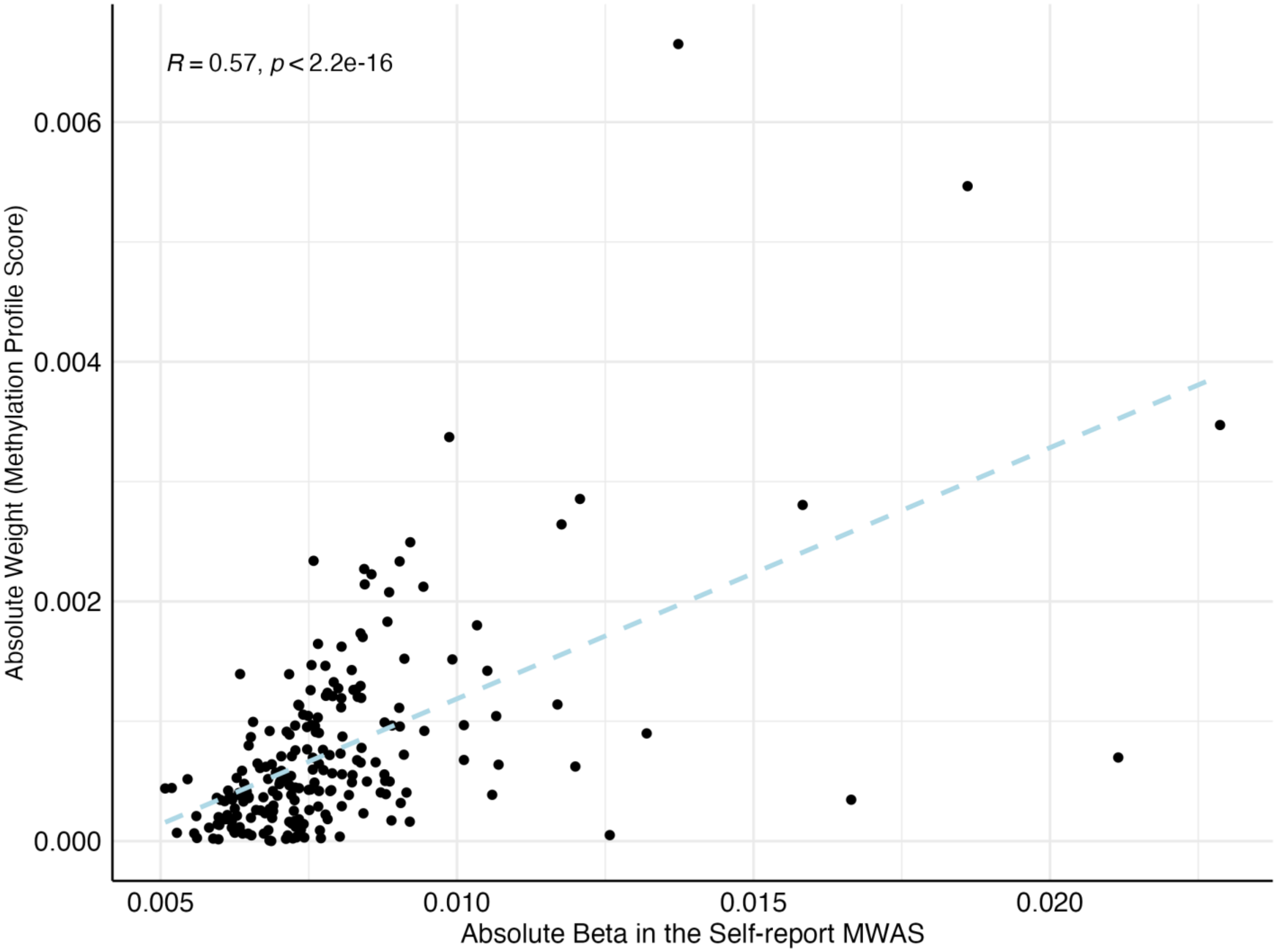

Supplementary Fig. 24: The weight per CpG in the methylation profile score vs the effect estimates from the self-report methylome-wide association study.

The beta effect sizes from the self-report MWAS (n = 16,531 participants) and the weights assigned to the CpG in the least absolute shrinkage and selection operator (LASSO) regression for the 212 CpGs included in the methylation profile score. The light blue dashed line is the regression line between the two variables and the Pearson correlation coefficient and corresponding P value, calculated using the stat_cor function in ggpubr R package[1] is displayed.

MPS calculation in external cohorts

Finn Twin Cohort

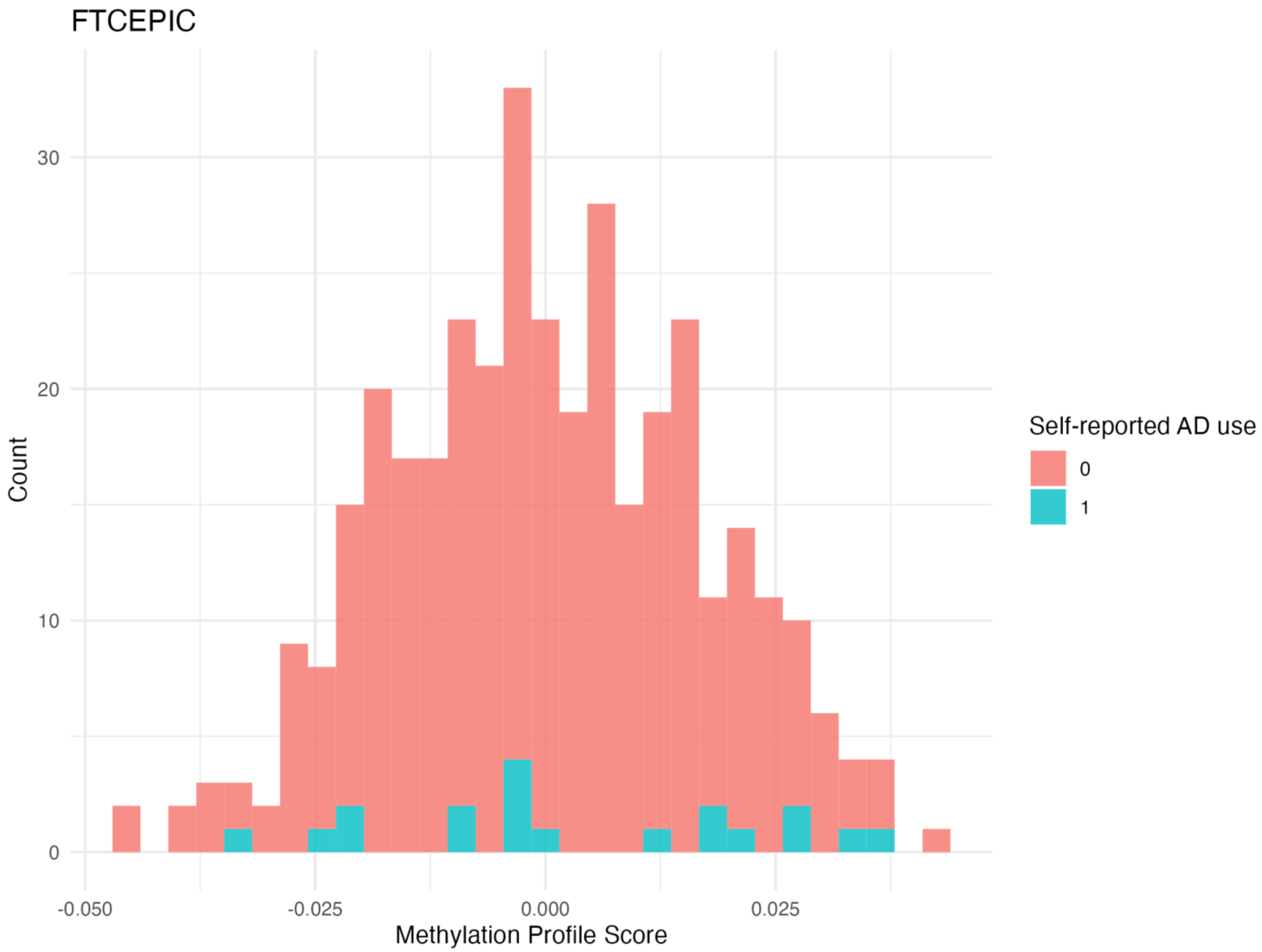

Supplementary Fig. 25: The distribution of the antidepressant methylation profile score in Finn Twin 12 & Finn Twin 16 (EPIC array).

The distribution of antidepressant exposure methylation profile score (MPS) in antidepressant-exposed (1) and antidepressant-unexposed (0) individuals in FinnTwin12 & FinnTwin16 (which used Illumina EPIC array) ($n_{exposed}$ = 19, $n_{unexposed}$ = 344).

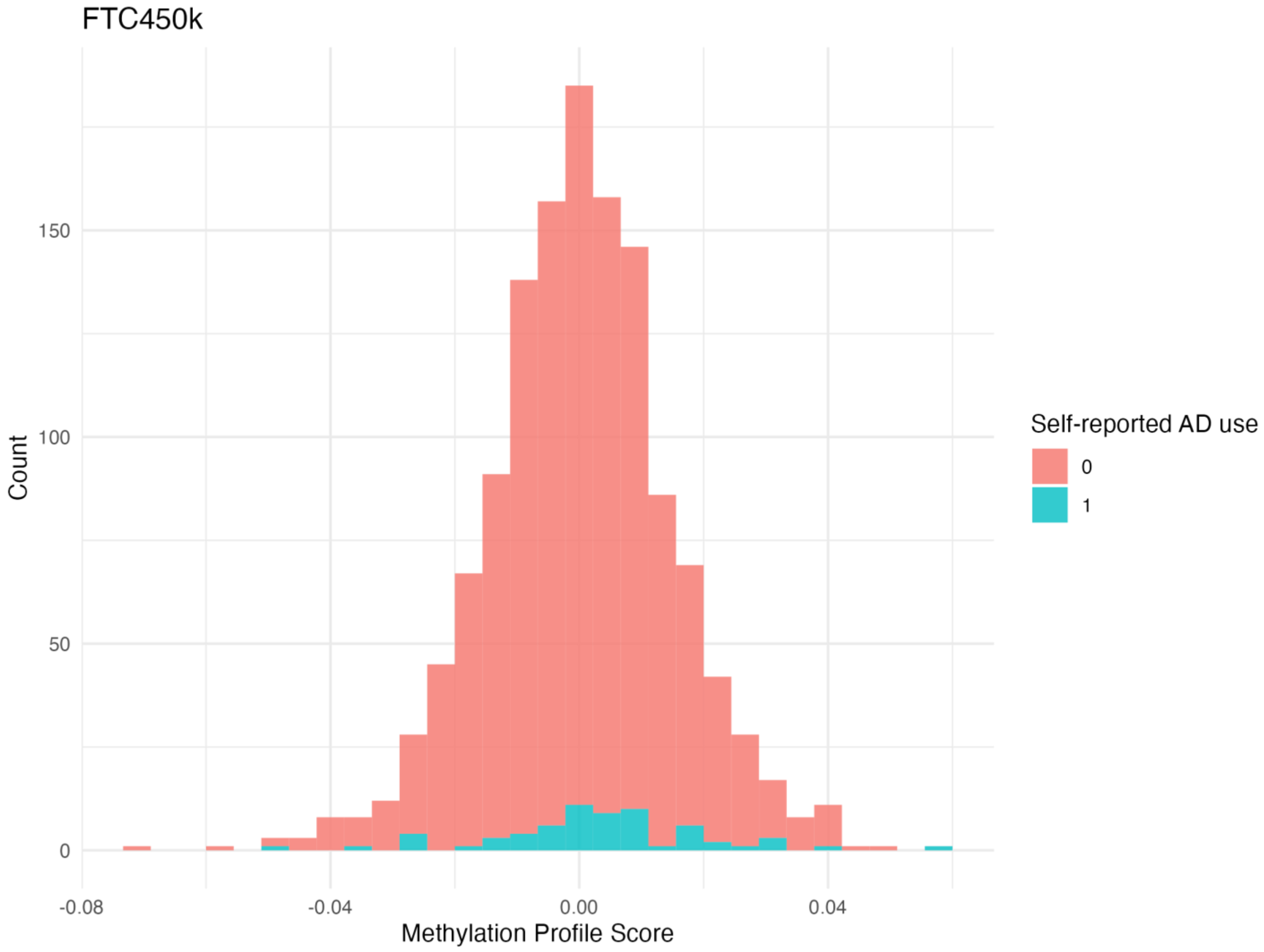

Supplementary Fig. 26: The distribution of the antidepressant methylation profile score in the Older Finnish Twin Cohort (450K array).

The distribution of antidepressant exposure methylation profile score (MPS) in antidepressant-exposed (1) and antidepressant-unexposed (0) in the Older Twin Cohort (which used Illumina 450K array) ($n_{exposed}$ = 65, $n_{unexposed}$ = 1250)

SHIP-TREND

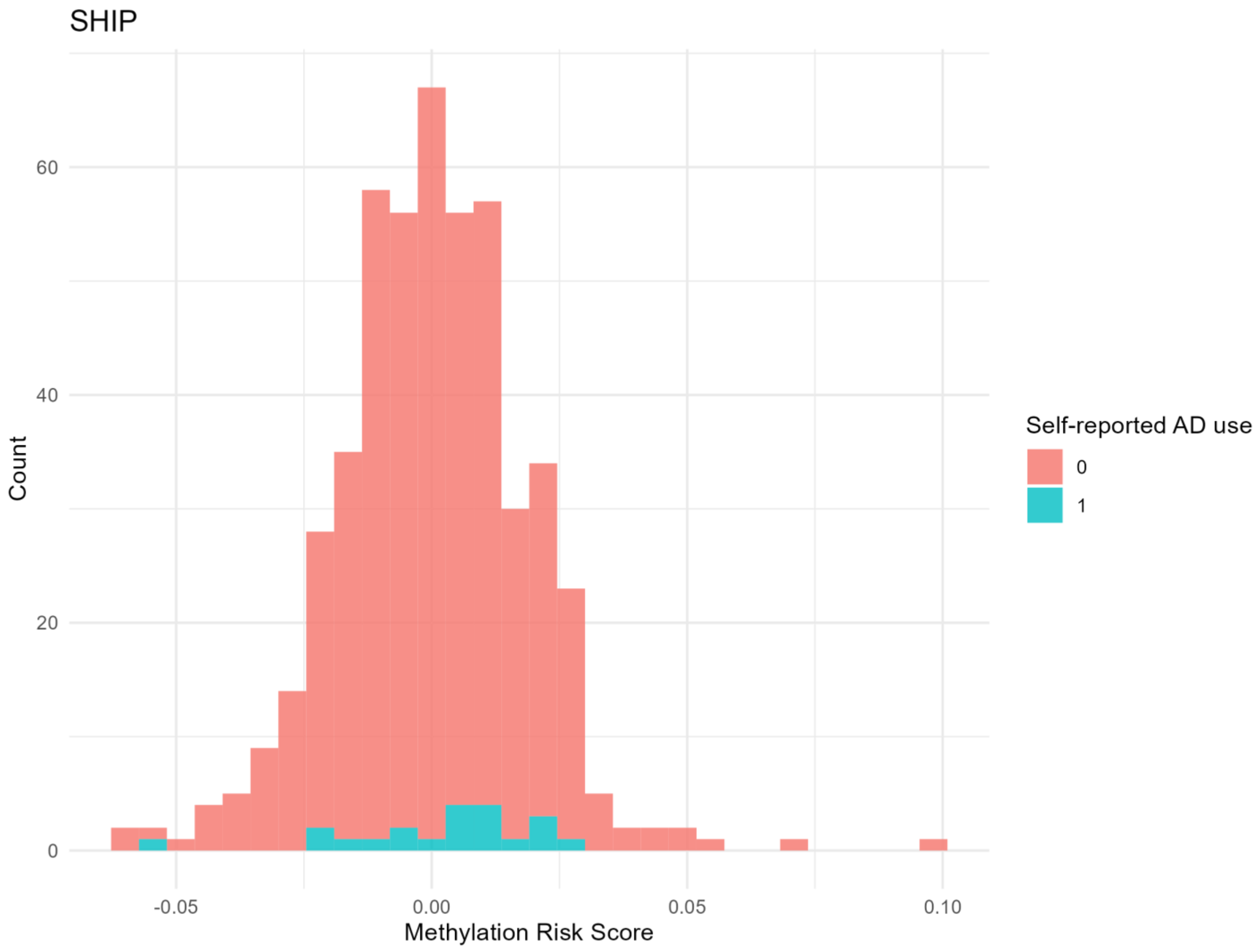

Supplementary Fig. 27: The distribution of the antidepressant methylation profile score in the Study of Health In Pomerania (SHIP)-TREND cohort.

The distribution of antidepressant exposure methylation profile score (MPS) in antidepressant-exposed (1) and antidepressant-unexposed (0) in the SHIP-TREND Cohort ($n_{exposed}$ = 21, $n_{unexposed}$ = 474).

FOR2107

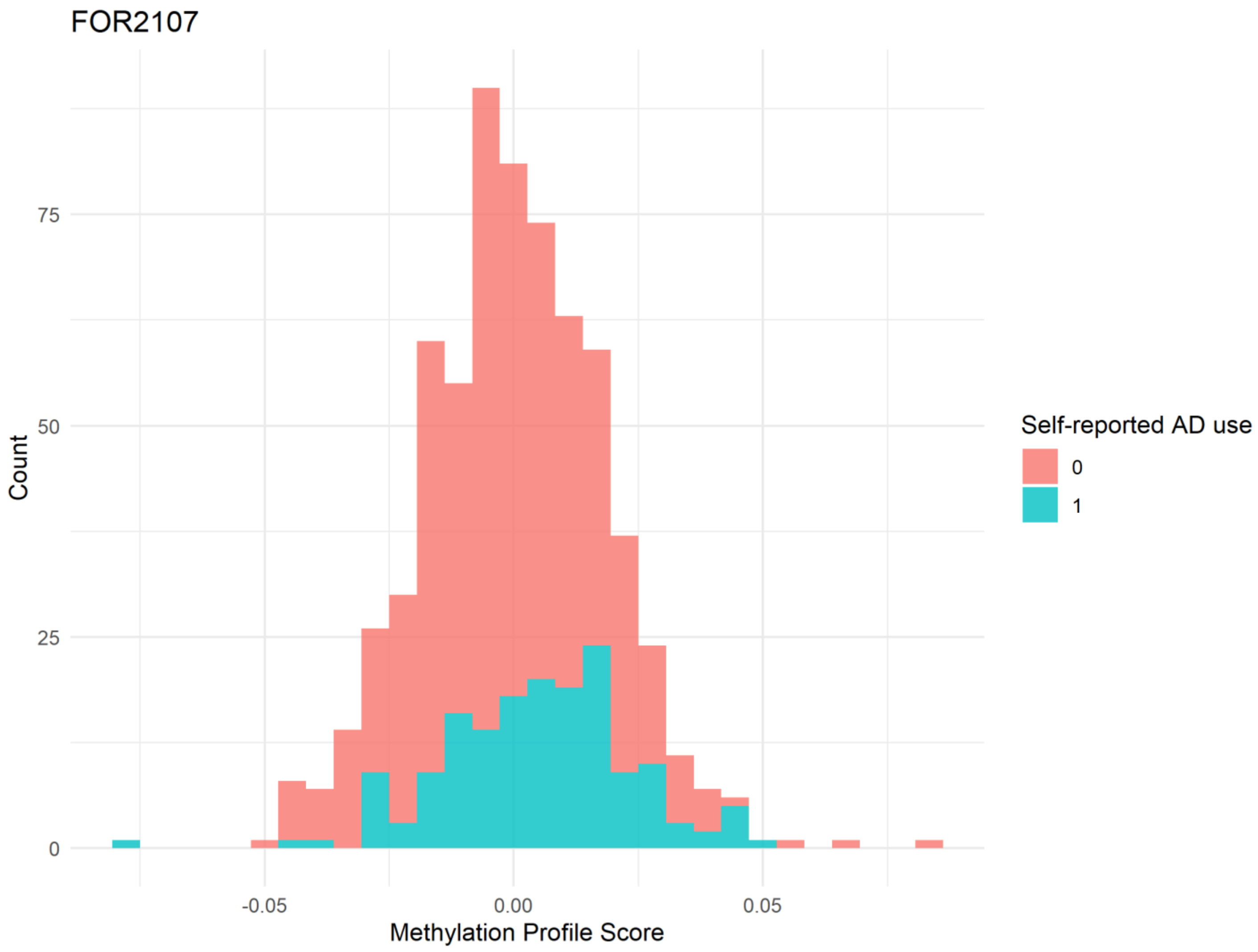

Supplementary Fig. 28: The distribution of the antidepressant methylation profile score in the FOR2107 cohort.

The distribution of antidepressant exposure methylation profile score (MPS) in antidepressant-exposed (1) and antidepressant-unexposed (0) in the FOR2107 cohort ($n_{exposed}$ = 165, $n_{unexposed}$ = 493).

NTR

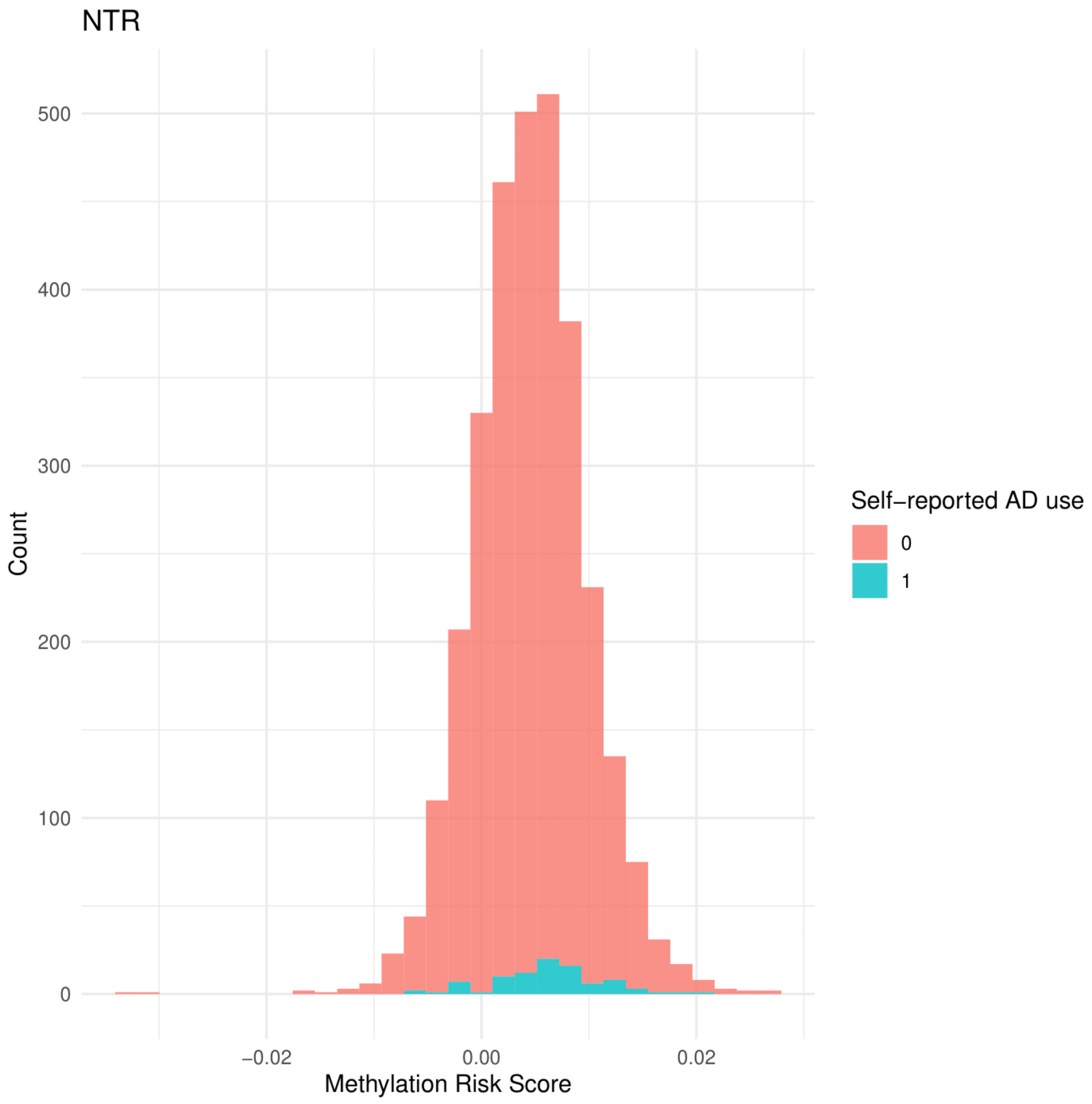

Supplementary Fig. 29: The distribution of the antidepressant methylation profile score in the Netherlands Twin Register (NTR) cohort.

The distribution of antidepressant exposure methylation profile score (MPS) in antidepressant-exposed (1) and antidepressant-unexposed (0) in the NTR cohort ($n_{exposed}$ = 89, $n_{unexposed}$ = 2998).

MARS-UniDep

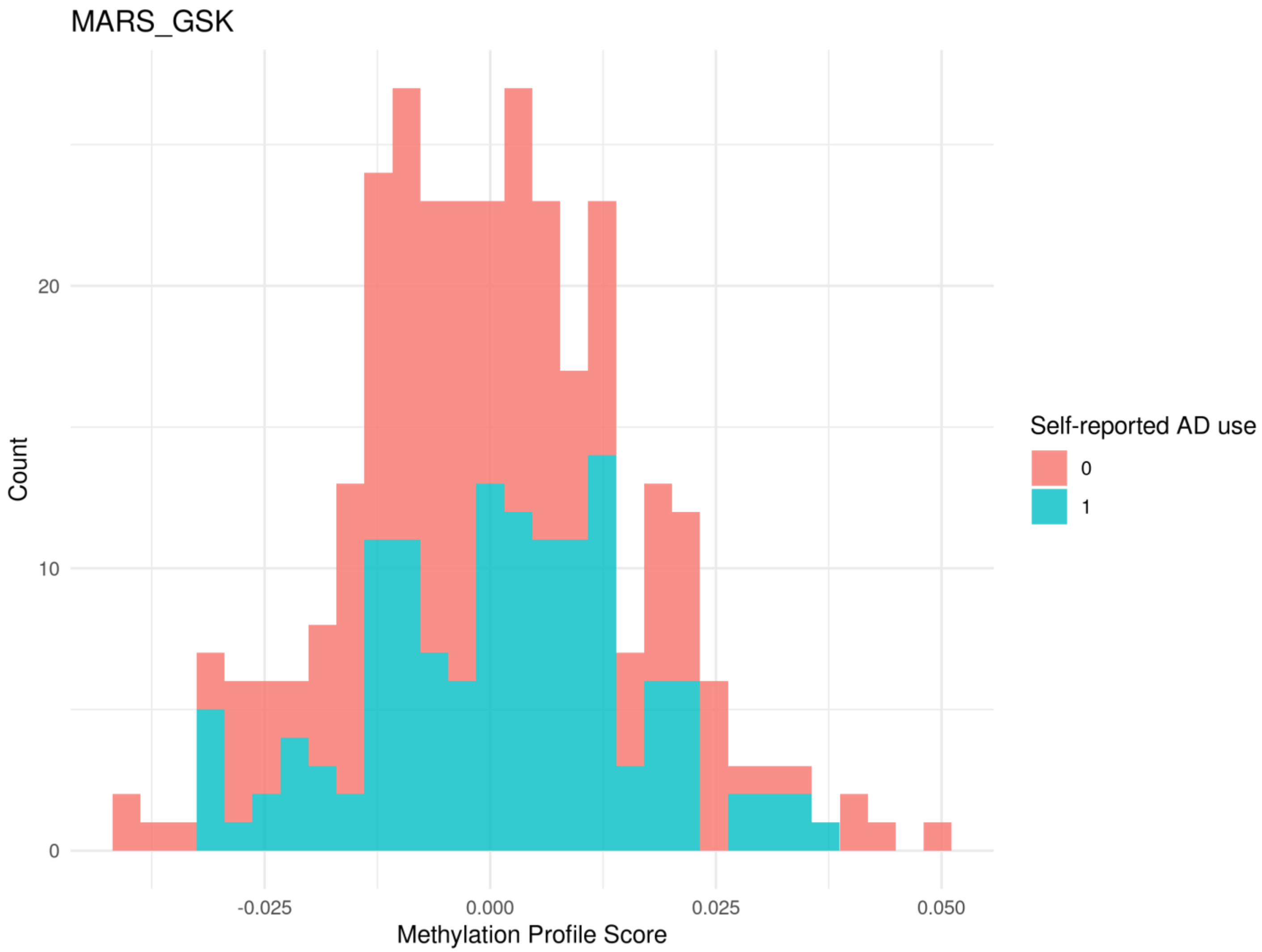

Supplementary Fig. 30: The distribution of the antidepressant methylation profile score in the Munich Antidepressant Response Study (MARS)-UniPolar Depression (UniDep) cohort.

The distribution of antidepressant exposure methylation profile score (MPS) in antidepressant-exposed (1) and antidepressant-unexposed (0) in the MARS-UniDep Cohort ($n_{exposed}$ = 135, $n_{unexposed}$ = 177). NB: The UniDep cohort is sometimes referred to as 'GSK'.

LBC1936

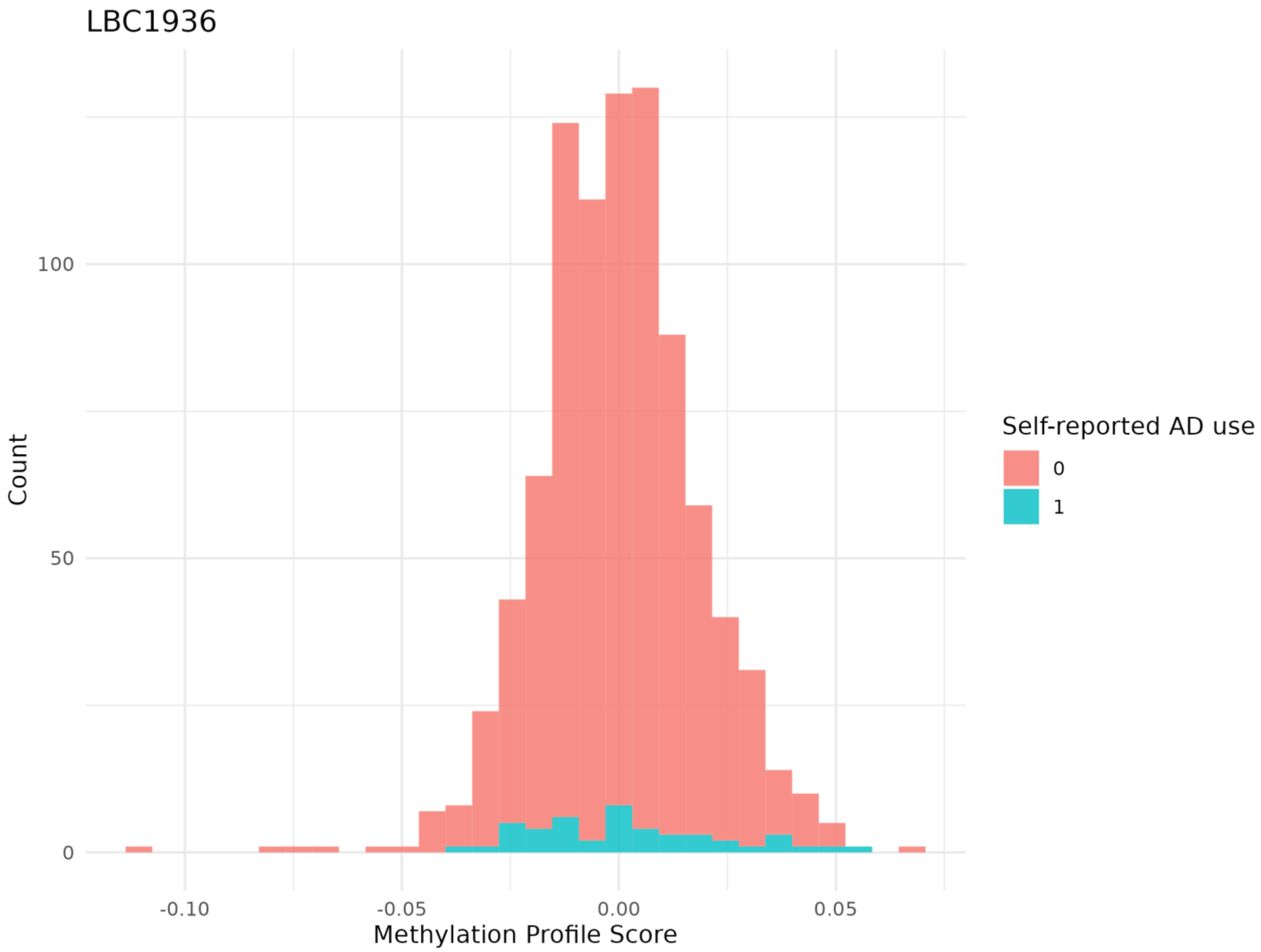

Supplementary Fig. 31: The distribution of the antidepressant methylation profile score in the Lothian Birth Cohorts 1936 (LBC1936) cohort.

The distribution of antidepressant exposure methylation profile score (MPS) in antidepressant-exposed (1) and antidepressant-unexposed (0) in the LBC1936 Cohort ($n_{exposed}$ = 46, $n_{unexposed}$ = 843).

ALSPAC

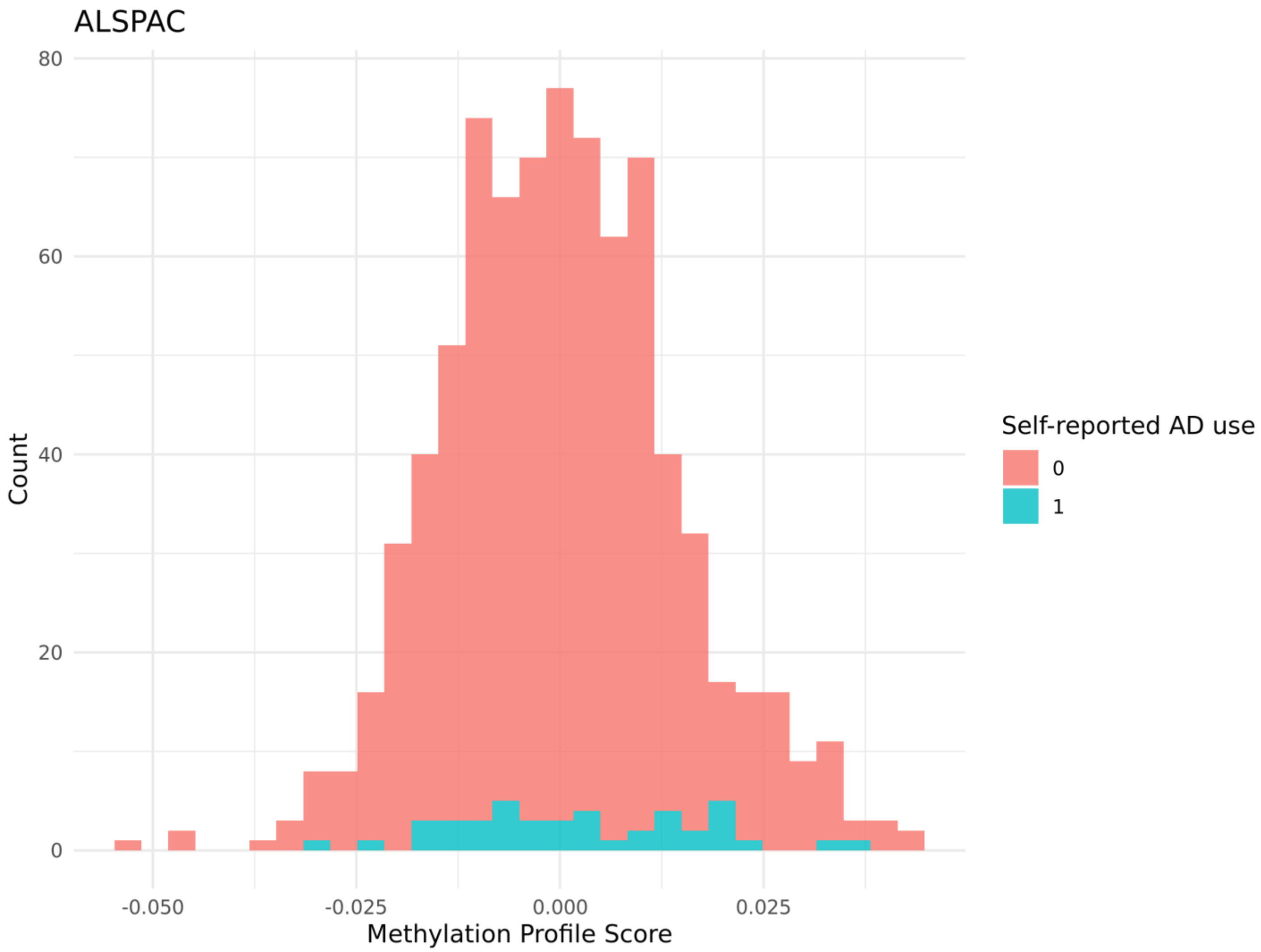

Supplementary Fig. 32: The distribution of the antidepressant methylation profile score in the Avon Longitudinal Study of Parents and Children (ALSPAC) cohort.

The distribution of antidepressant exposure methylation profile score (MPS) in antidepressant-exposed (1) and antidepressant-unexposed (0) in the ALSPAC Cohort ($n_{exposed}$ = 43, $n_{unexposed}$ = 758).

ERISK

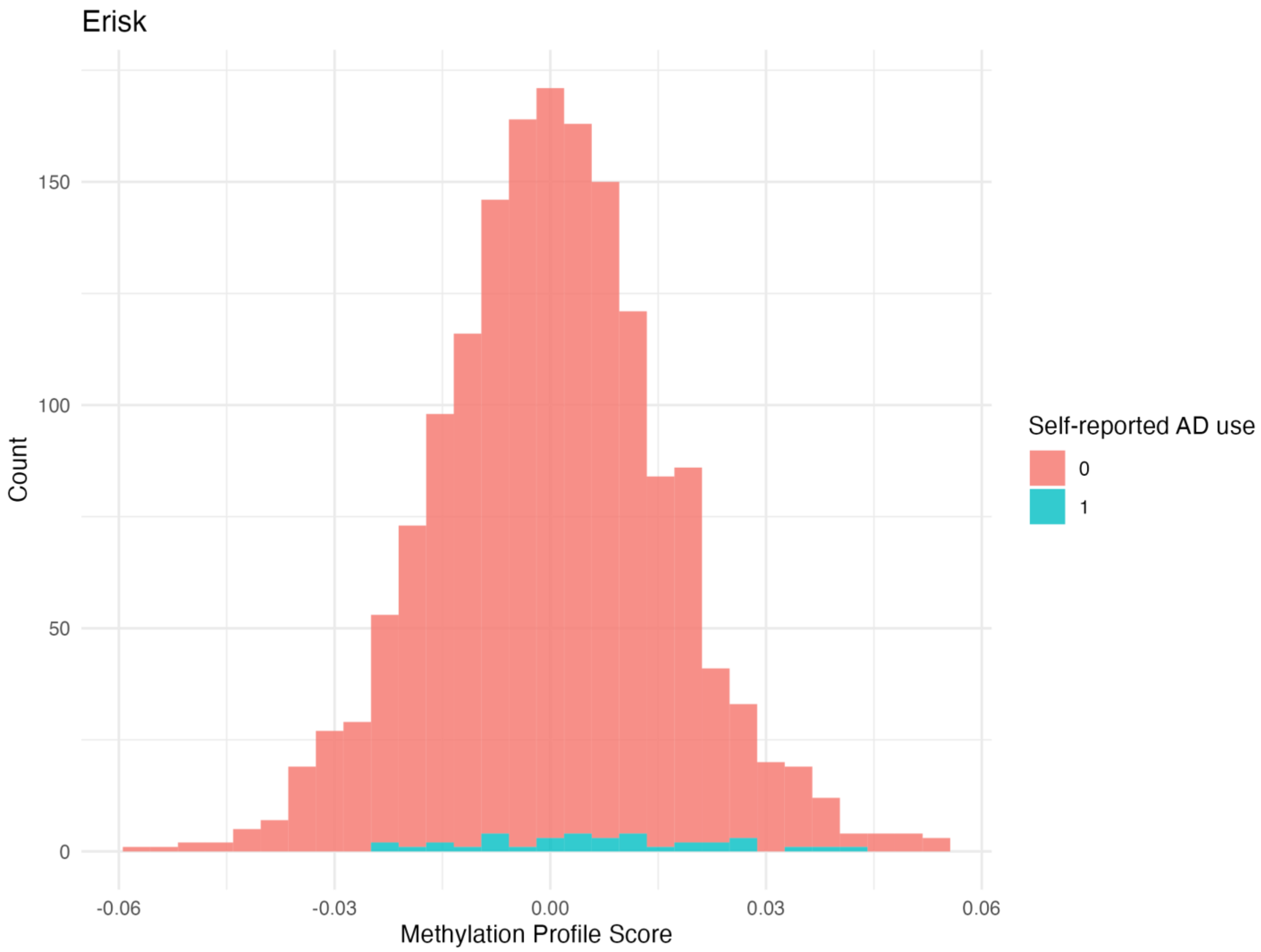

Supplementary Fig. 33: The distribution of the antidepressant methylation profile score in the Environmental risk (E-Risk) Longitudinal Twin Study cohort.

The distribution of antidepressant exposure methylation profile score (MPS) in antidepressant-exposed (1) and antidepressant-unexposed (0) in the E-Risk Cohort ($n_{exposed}$ = 36, $n_{unexposed}$ = 1622).

MPS calculation in prospective Generation Scotland follow up

STRADL

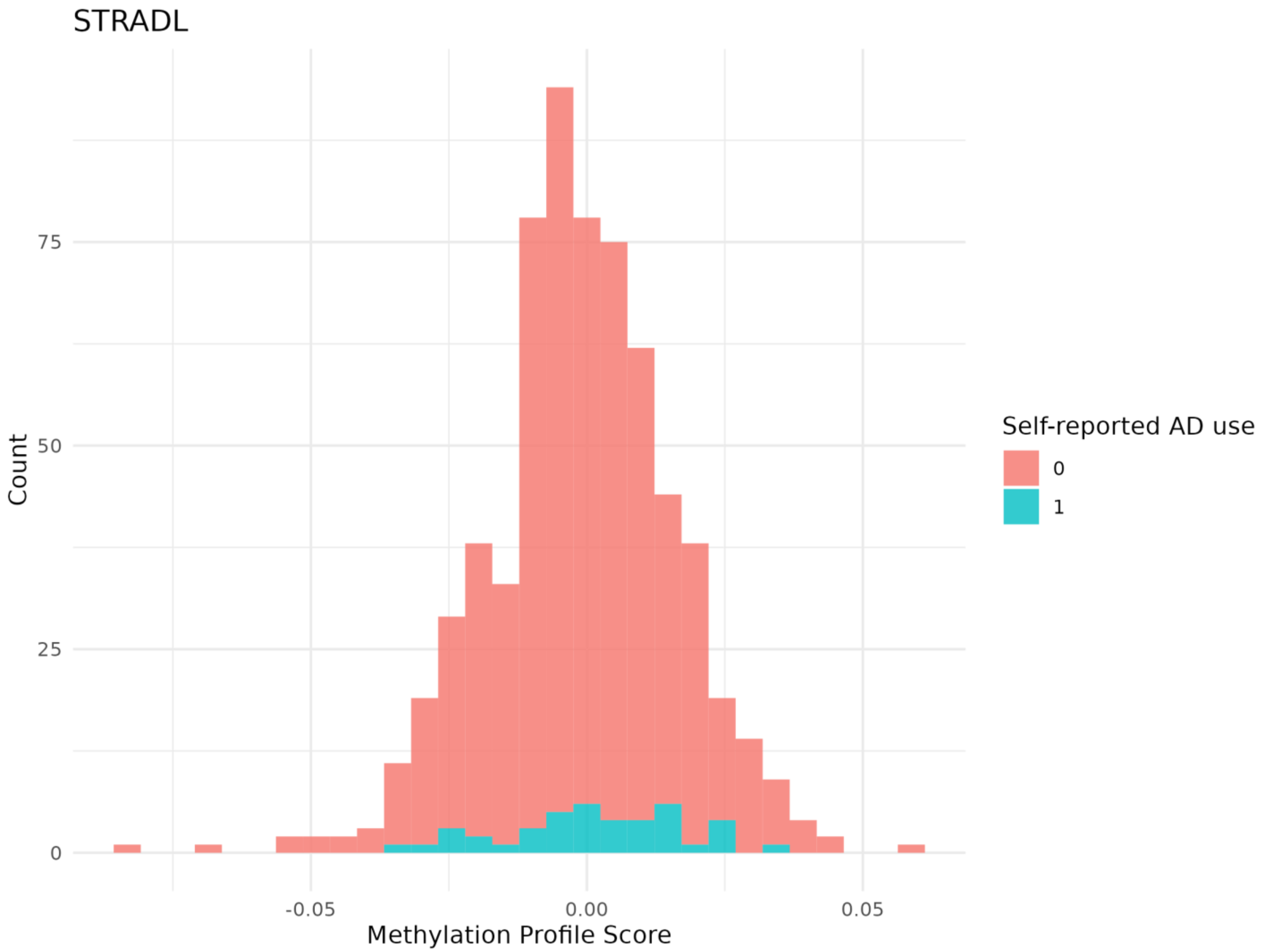

Supplementary Fig. 34: The distribution of the antidepressant methylation profile score in the Stratifying Resilience and Depression Longitudinally (STRADL) cohort.

The distribution of antidepressant exposure methylation profile score (MPS) in antidepressant-exposed (1) and antidepressant-unexposed (0) in the STRADL subcohort ($n_{exposed}$ = 42, $n_{unexposed}$ = 617). Note that this is a prospective subcohort of Generation Scotland (GS) at a 5-year follow-up. All individuals self-reported not taking antidepressants at baseline (GS) timepoint, and those who self-reported to taking antidepressants at the STRADL time point are classed as antidepressant-exposed and those who remain self-reporting no antidepressant exposure are classed as antidepressant-unexposed.

Antidepressant exposure ~ MPS associational models

Singular cohort results

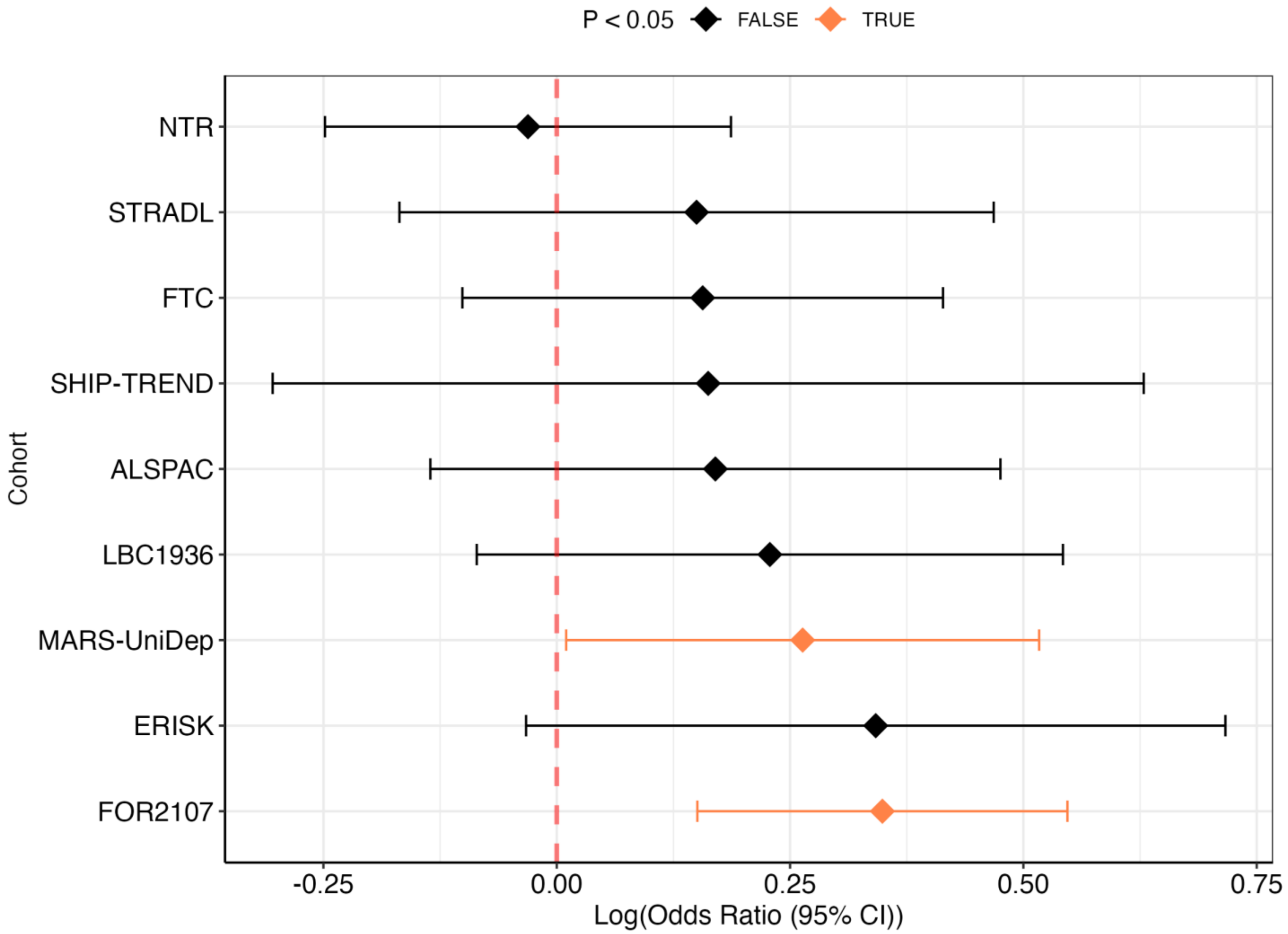

Supplementary Fig. 35: The association of the antidepressant exposure methylation profile score with measured antidepressant exposure in each external cohort.

The log(Odds Ratio) (central point) of antidepressant methylation profile score (MPS) association with antidepressant exposure in external cohorts, with 95% confidence intervals (bars). 95% Confidence intervals were calculated as the Odds Ratio +- 1.96*Standard Error (n_{NTR}~=3,087, n_{STRADL}=658, n_{FTC}=1,678, $n_{SHIP-TREND}$=495, n_{ALSPAC}=801, $n_{LBC1936}$=889, $n_{MARS-UniDep}$=312, n_{ERISK}=1,658, $n_{FOR2107}$=658). The associsation between the MPS and antidepressant exposure was calculated using either a generalised linear model (FOR2107 and ALSPAC), a generalised linear mixed model (SHIP-Trend, LBC1936, MARS-UniDep, STRADL and E-Risk) and generalised estimation equation model (FTC and NTR).The bars are colored based on whether there was a significant association ($p < 0.05$) between the MPS and antidepressant exposure (orange = significant, black = non-significant). MARS-UniDep ($p = 0.042$) and FOR2107 ($p = 0.00056$) showed a significant association. NTR = Netherlands Twin Register, STRADL = Stratifying Depression and Resilience Longitudinally, FTC = Finnish Twin

Cohort, SHIP-TREND = Study of Health In Pomerania -TREND, ALSPAC = Avon Longitudinal Study of Parents and Children, LBC1936 = Lothian Birth Cohorts 1936, MARS-UniDep = Munich Antidepressant Response Study-UniPolar Depression Study, ERISK = Environmental Risk Longitudinal Twin Study, FOR2107 = FOR2107 consortium.

Prescription data

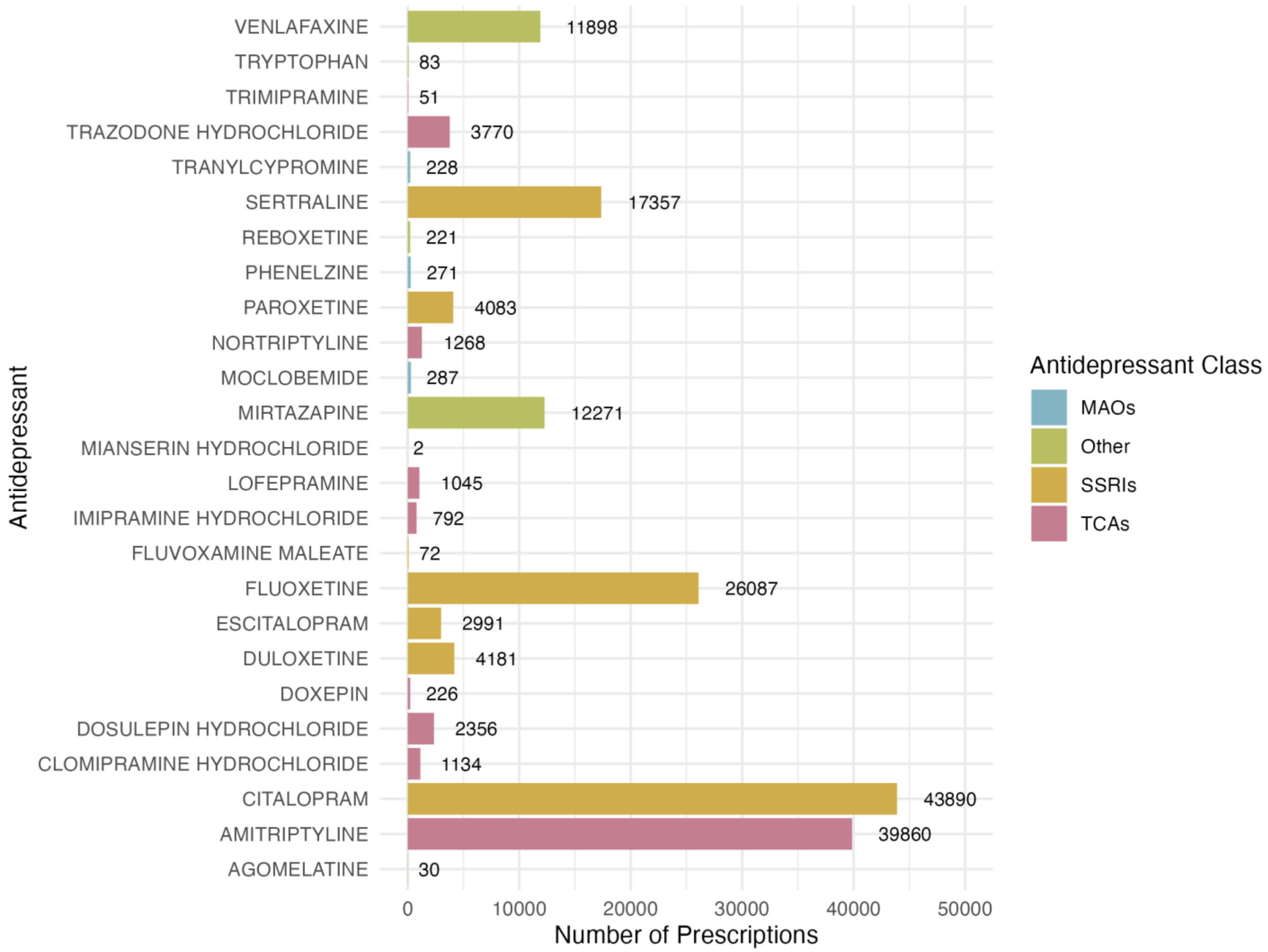

Supplementary Fig. 36: Counts of antidepressant prescriptions in Generation Scotland (2009-2017).

All antidepressant prescriptions for individuals in Generation Scotland between 2009 and 2017 (n = 7,544).

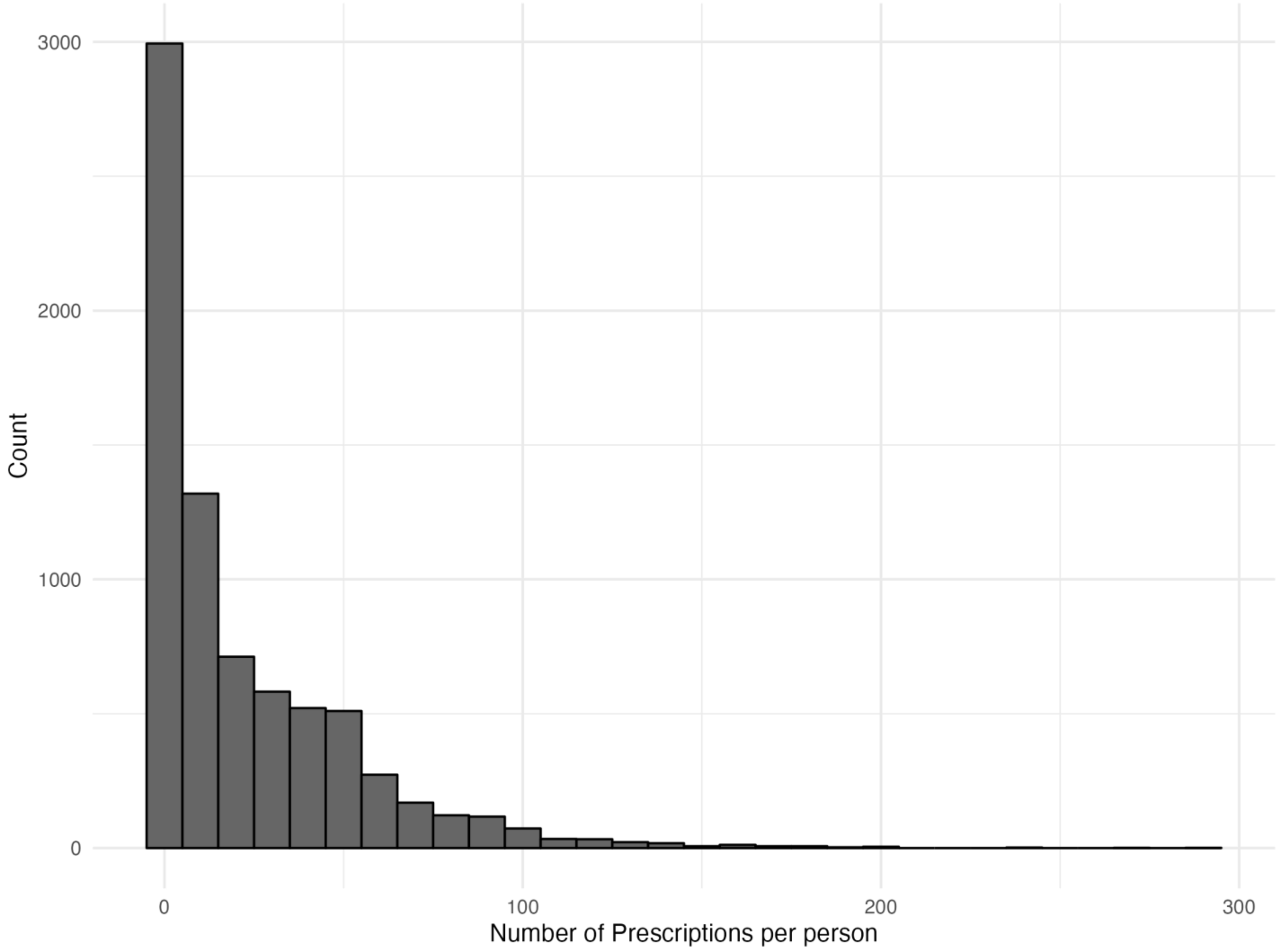

Supplementary Fig. 37: Distribution of the number of antidepressant prescriptions per individual.

Histogram of the number of antidepressant prescriptions per person between 2009-2017 (n = 7,544 participants).

Drug Dosages

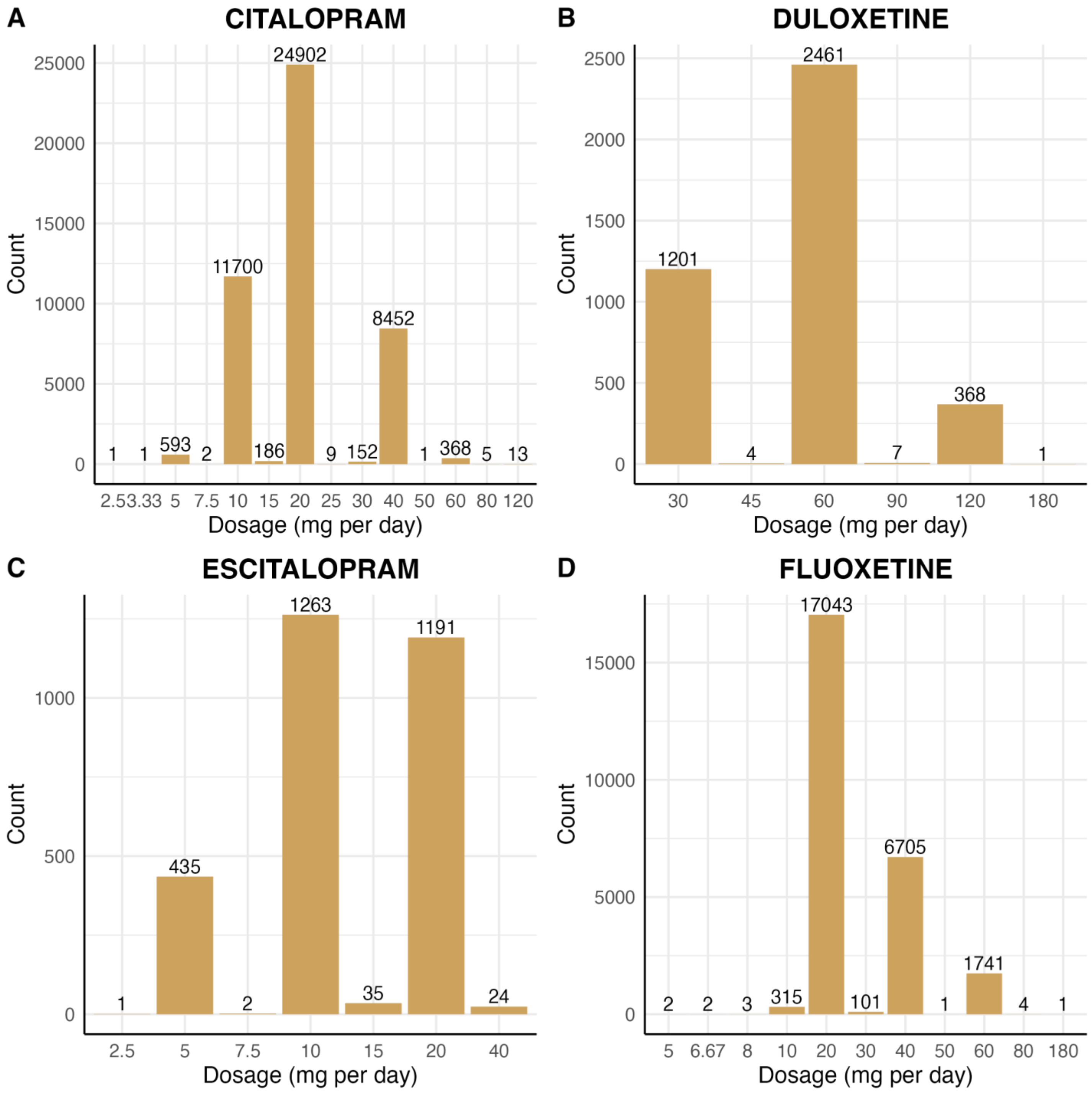

Supplementary Fig. 38: Counts of prescription dosages for Citalopram, Duloxetine, Escitalopram and Fluoxetine.

Prescription dosages for A) Citalopram, B) Duloxetine, C) Escitalopram and D) Fluoxetine after parsing prescription instructions.

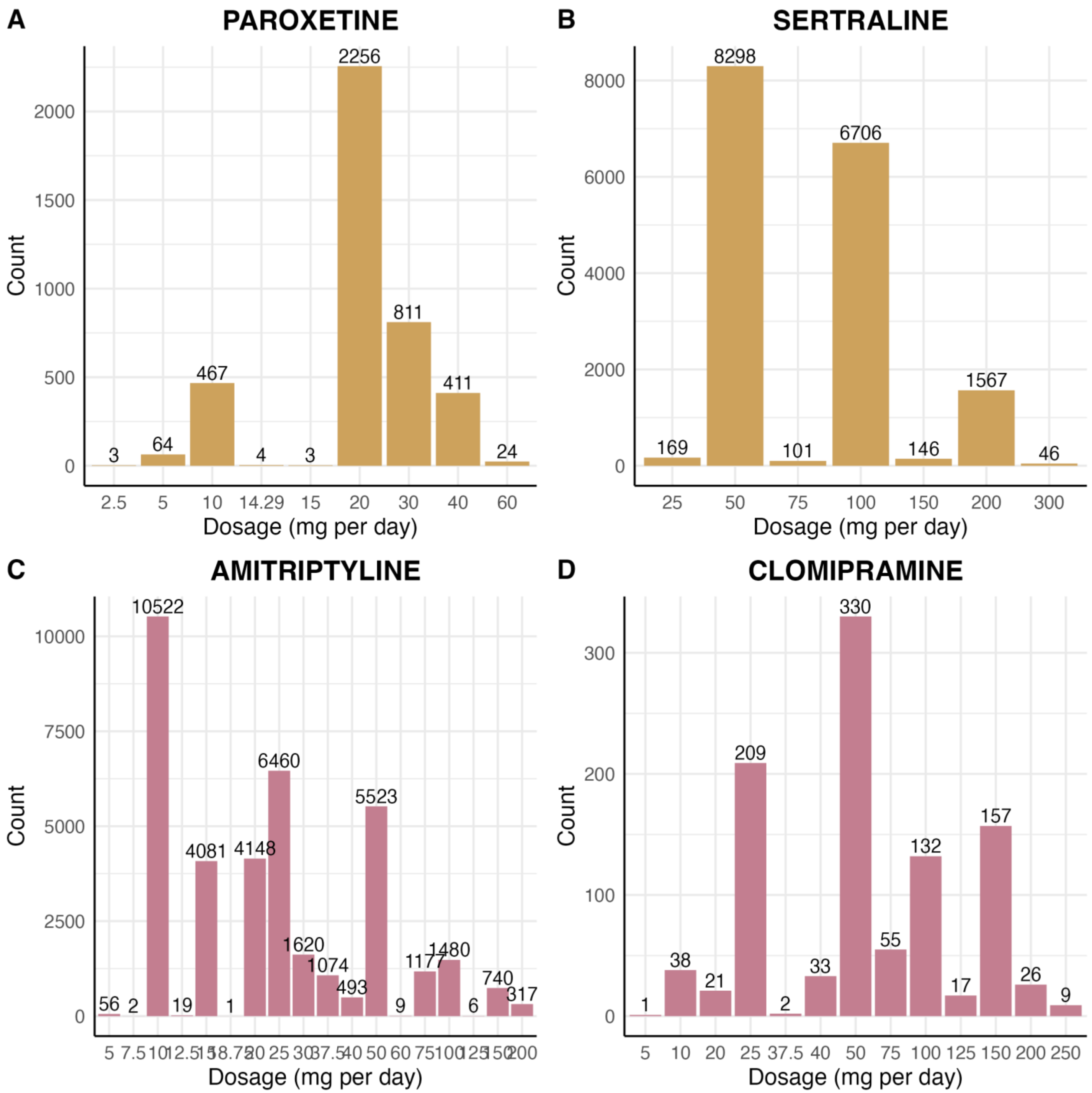

Supplementary Fig. 39: Counts of prescription dosages for Paroxetine, Sertraline, Amitriptyline and Clomipramine.

Prescription dosages for A) Paroxetine, B) Sertraline, C) Amitriptyline and D) Clomipramine after parsing prescription instructions. Color represents the class of antidepressant: Selective serotonin re-uptake inhibitors (SSRIs) are shown in orange, and Tricyclic Antidepressants (TCAs) in pink.

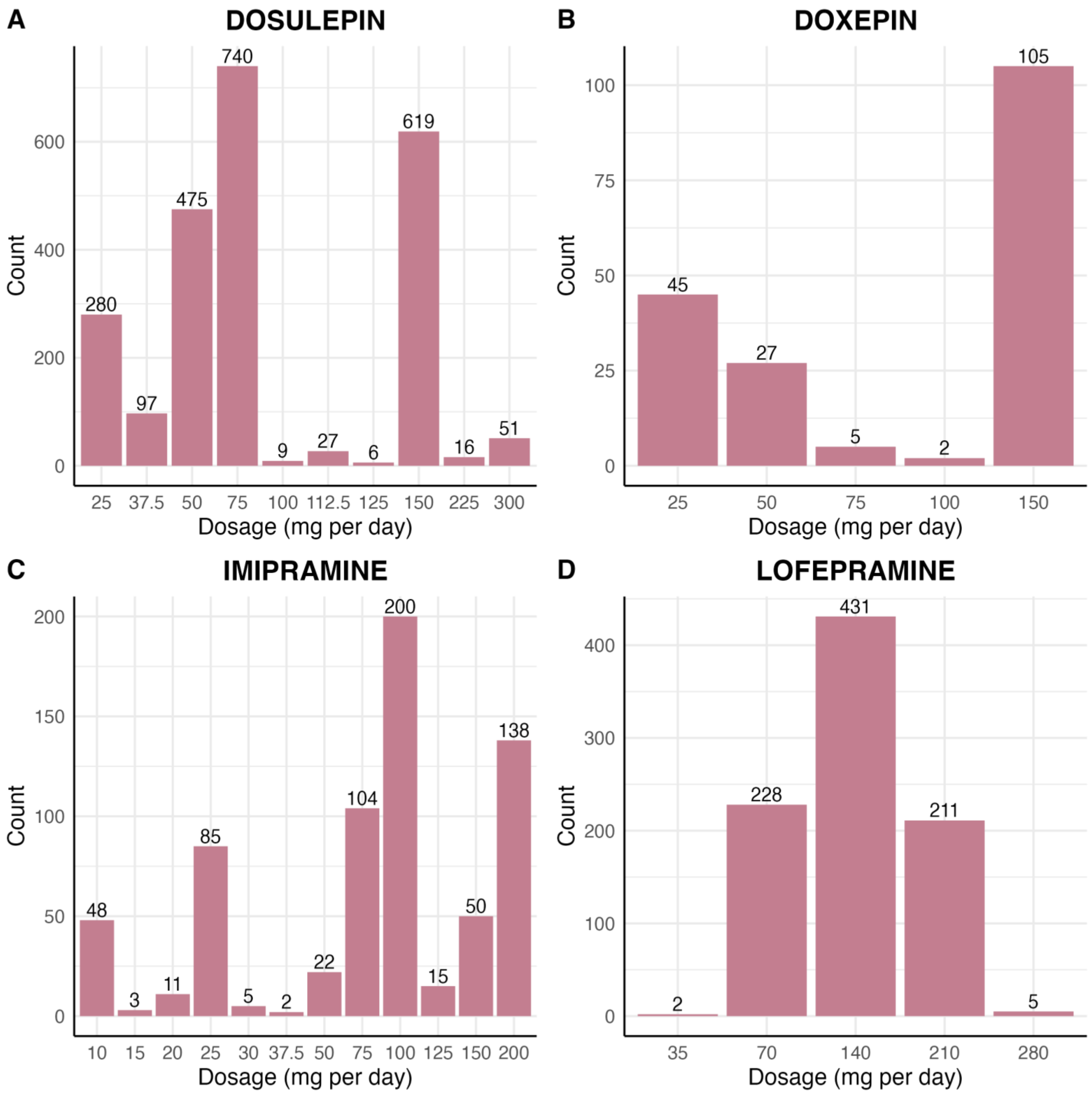

Supplementary Fig. 40: Counts of prescription dosages for Dosulepin, Doxepin, Imipramine and Lofepramine

Prescription dosages for A) Dosulepin, B) Doxepin, C) Imipramine, D) Lofepramine after parsing prescription instructions.

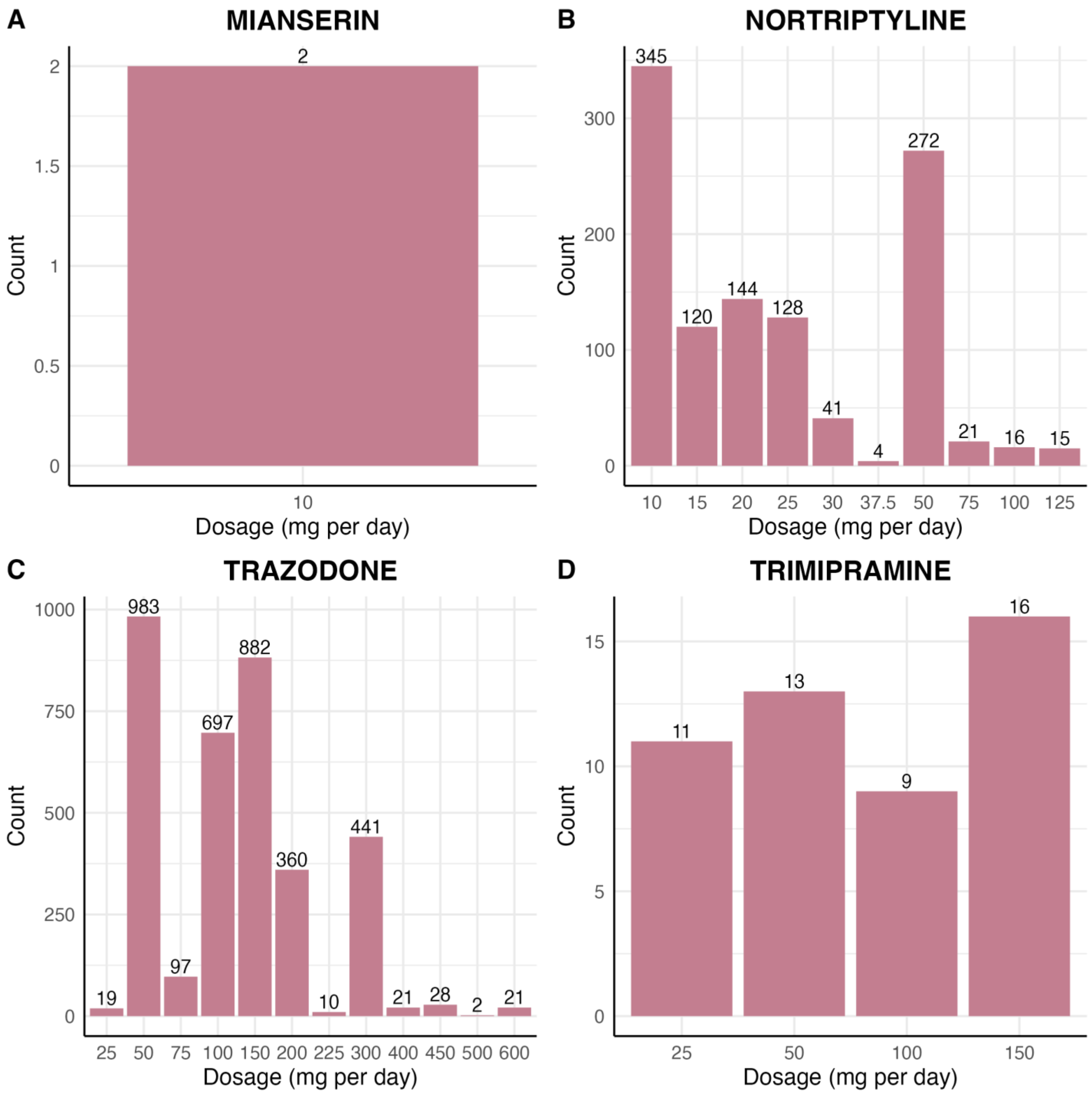

Supplementary Fig. 41: Counts of prescription dosages for Mianserin, Nortriptyline, Trazodone and Trimipramine.

Prescription dosages for A) Mianserin, B) Nortriptyline, C) Trazodone and D) Trimipramine after parsing prescription instructions.

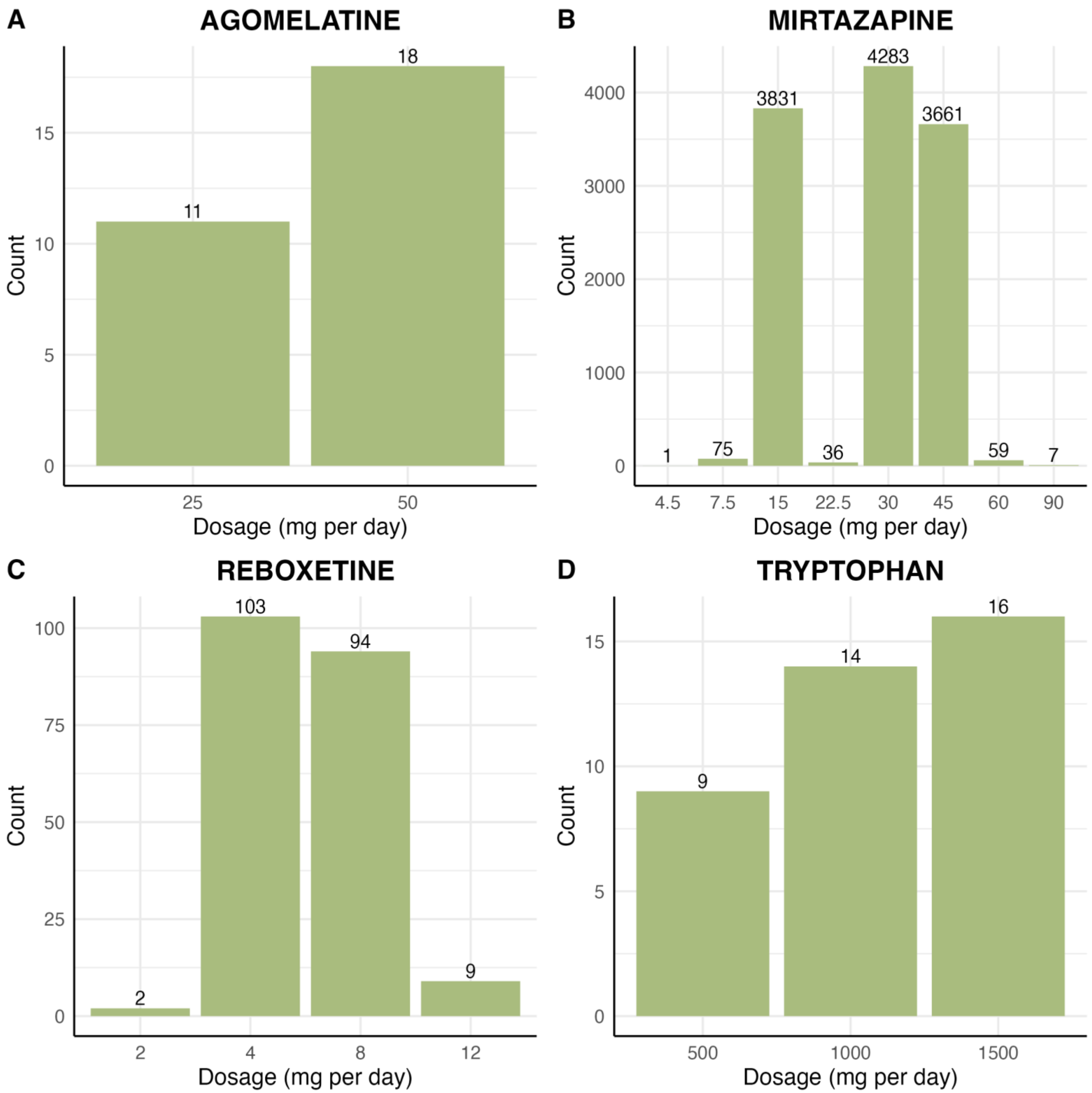

Supplementary Fig. 42: Counts of prescription dosages for Agomelatine, Mirtazapine, Reboxetine and Tryptophan.

Prescription dosages for A) Agomelatine, B) Mirtazapine, C) Reboxetine and D) Tryptophan after parsing prescription instructions.

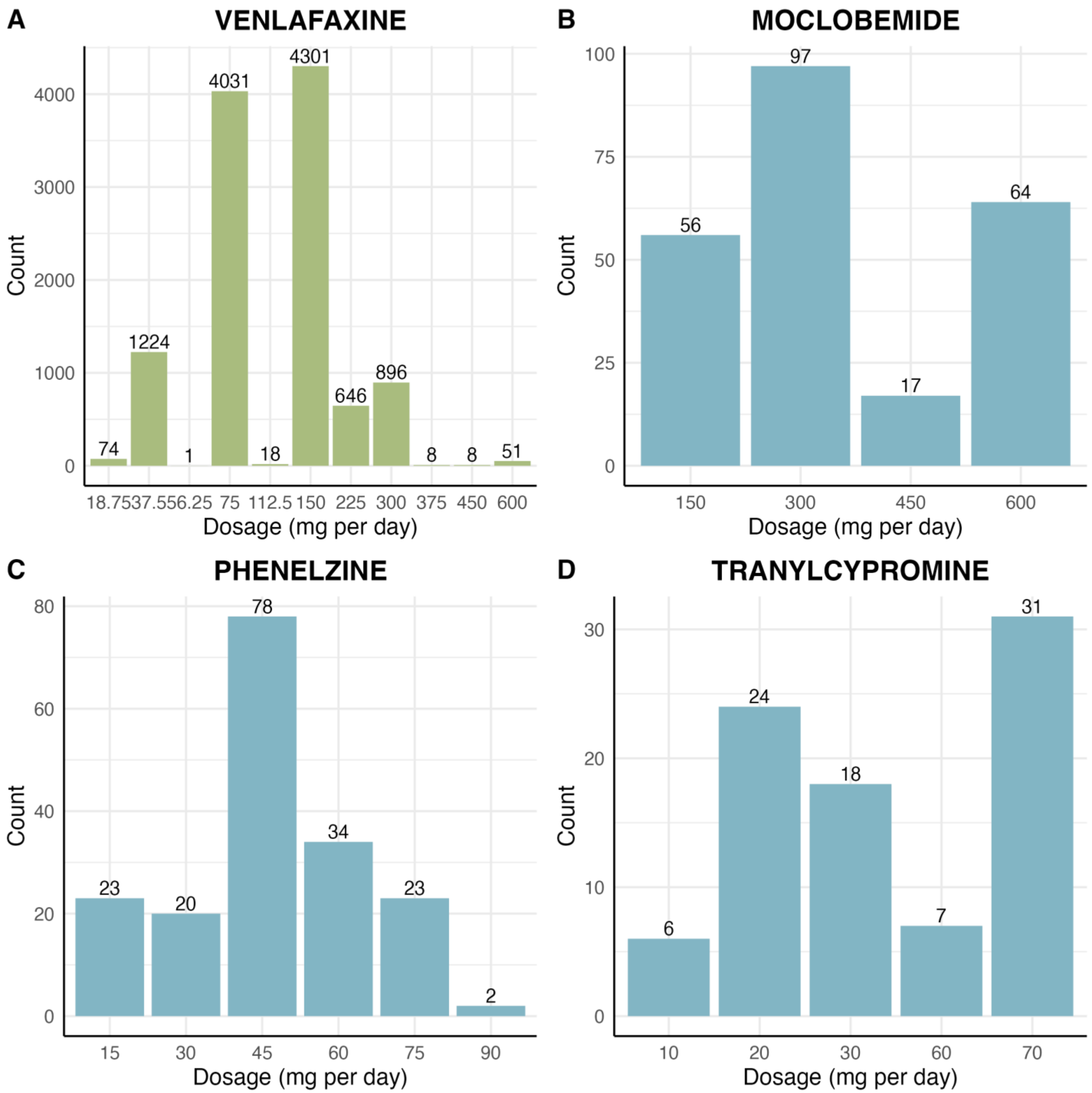

Supplementary Fig. 43: Counts of prescription dosages for Venlafaxine, Moclobemide, Phenelzine and Tranylcypromine.

Prescription dosages for A) Venlafaxine, B) Moclobemide, C) Phenelzine and D) Tranylcypromine after parsing prescription instructions. Color represents the class of antidepressant: Those classed as Other are shown in green, and Monoamine Oxidase Inhibitors (MOAs) in blue.

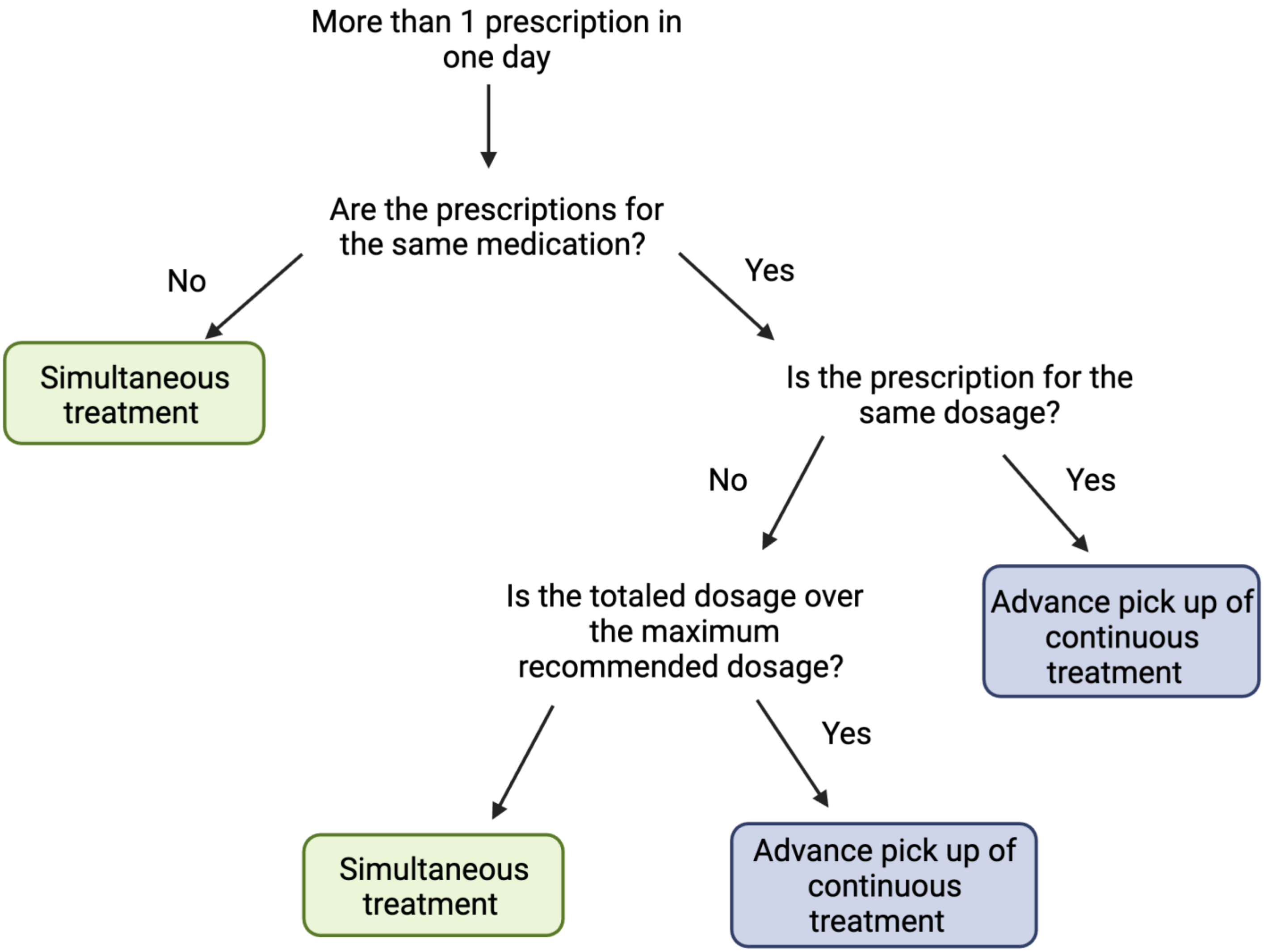

Supplementary Fig. 44: Methodology to parse whether multiple prescriptions in the same day are due to simultaneous treatment or advance pick up.

Decision tree of marking multiple prescriptions dispensed on the same day for the same individual. These events are marked as simultaneous treatment or continuous treatment collected in advance, which are treated as independent and merged prescription events respectively. Created in BioRender. Davyson, E. (2024) https://BioRender.com/q24t546

Multiple prescriptions in one day

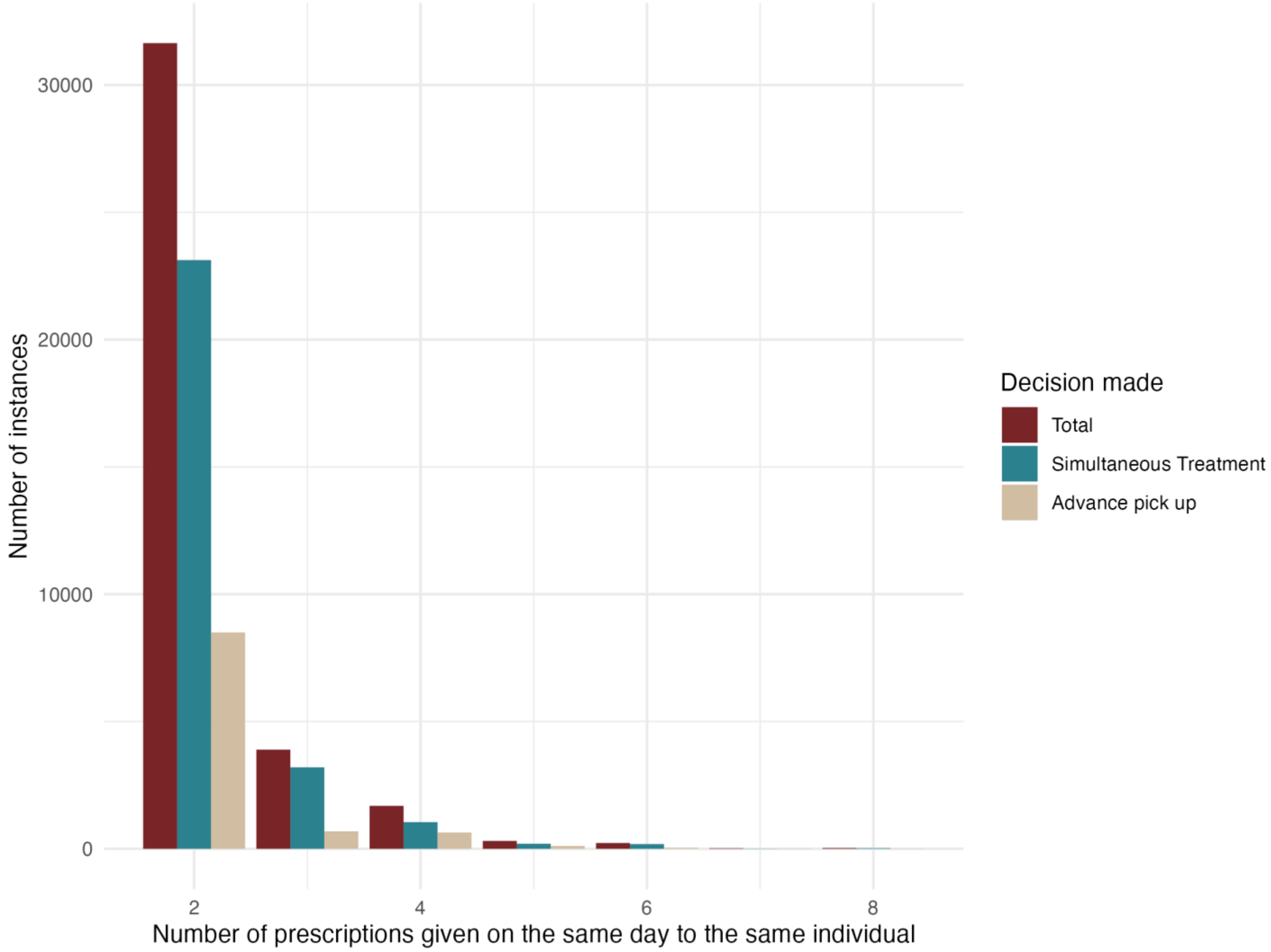

Supplementary Fig. 45: Outcomes of parsing multiple prescriptions in the same day as simultaneous treatment or advance pick up.

Bar plot of the number of instances in which multiple prescriptions were dispensed to the same individual on the same day (> 1) (n = 2,604 participants), and the proportion of these events which were assigned to simultaneous treatment and advance pick up from our decision tree process.

Prescription-derived antidepressant exposure phenotype

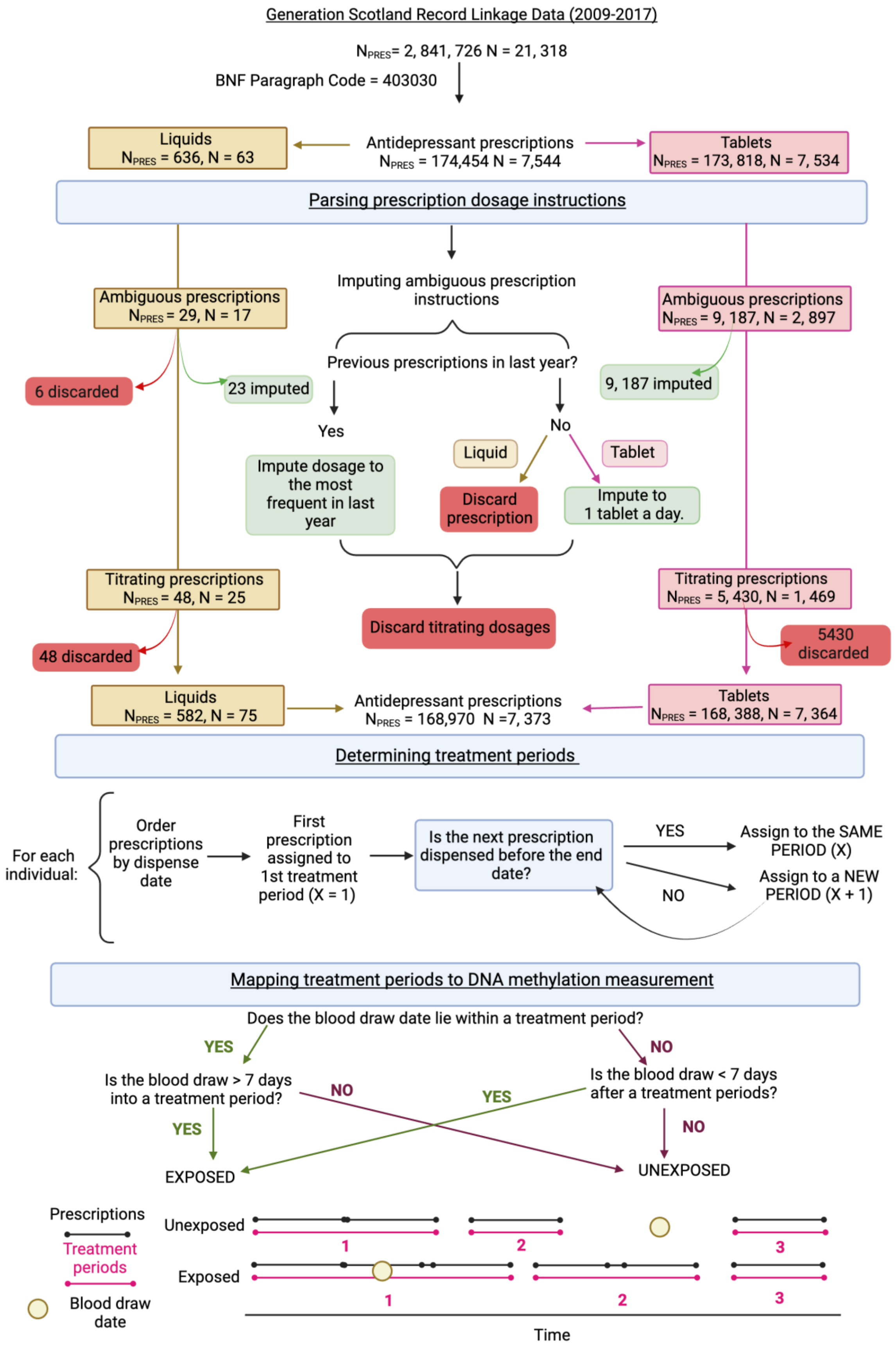

Supplementary Fig. 46: Methodology for determining antidepressant exposure at DNA methylation blood draw from prescription records.

Flow chart demonstrating the processing of prescription data, the derivation of antidepressant treatment periods and the mapping of treatment periods to the DNA methylation measurement. Prescription data was filtered to antidepressant prescriptions using the British National Formulary (0403030). Free-text prescription dosage instructions input by the dispenser were parsed in R using regex expressions. For ambiguous dosage instructions for tablet formulations, we imputed dosage instructions to the most frequent instruction in the past year for that individual OR to "1 tablet a day". For ambiguous prescription instructions for liquid formulations, we imputed instructions to the most frequent instruction in the past year or discarded the prescription event if this was not available. All prescriptions with titrating dosage instructions were discarded. Treatment periods were derived per individual by assessing whether subsequent antidepressant prescriptions were dispensed before or shortly after (+10% of estimated prescription duration) the previous prescription was expected to run out. We mapped treatment periods to the date of blood draw and DNA methylation measurement to assess antidepressant exposure. If a participant was > 7 days into a treatment period at blood draw or < 7 days since ending a treatment period, we classed them as antidepressant exposed. Conversely if a participant was not within a treatment period or < 7 days into a treatment period, they were classed as unexposed. Created in BioRender. Davyson, E. (2024) https://BioRender.com/v38j592

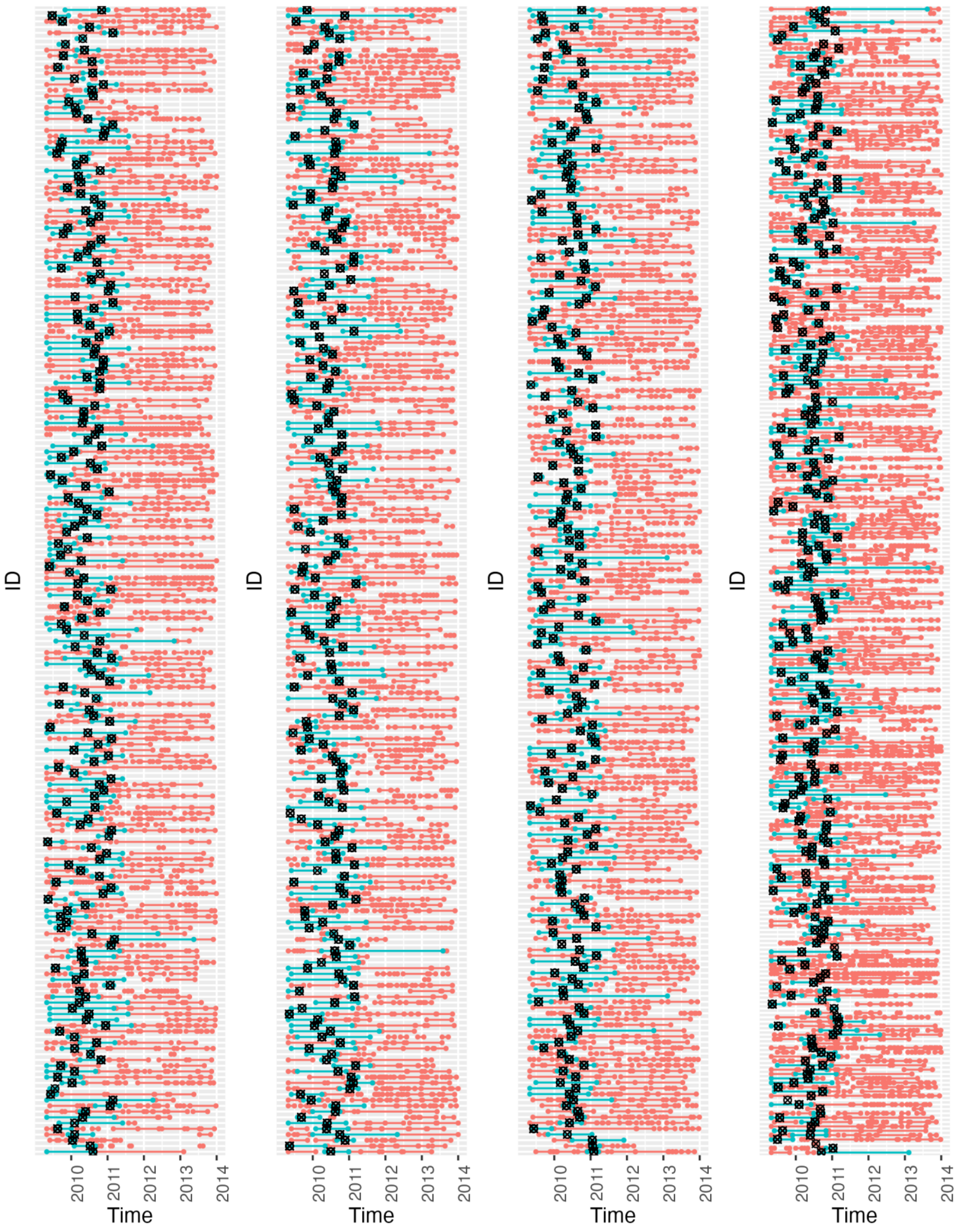
ID
Time
2010
2011
2012
2013
2014

Supplementary Fig. 47: All antidepressant treatment periods for those exposed at the time of DNA methylation measurement.

Antidepressant treatment periods for all antidepressant-exposed individuals (n = 861) in the prescription-derived phenotype over time (2009-2014). Each antidepressant treatment period is denoted by an individual bar. Antidepressant exposure is defined as those > 7 days into or < 7 days out of an antidepressant treatment period at the time of the blood draw. The time point of the blood-draw appointment (where DNA methylation measurement took place) is denoted by the black symbol. Each treatment periods is coloured depending on whether it encompasses the blood draw appointment (blue) or not (red).

Self-report antidepressant exposure

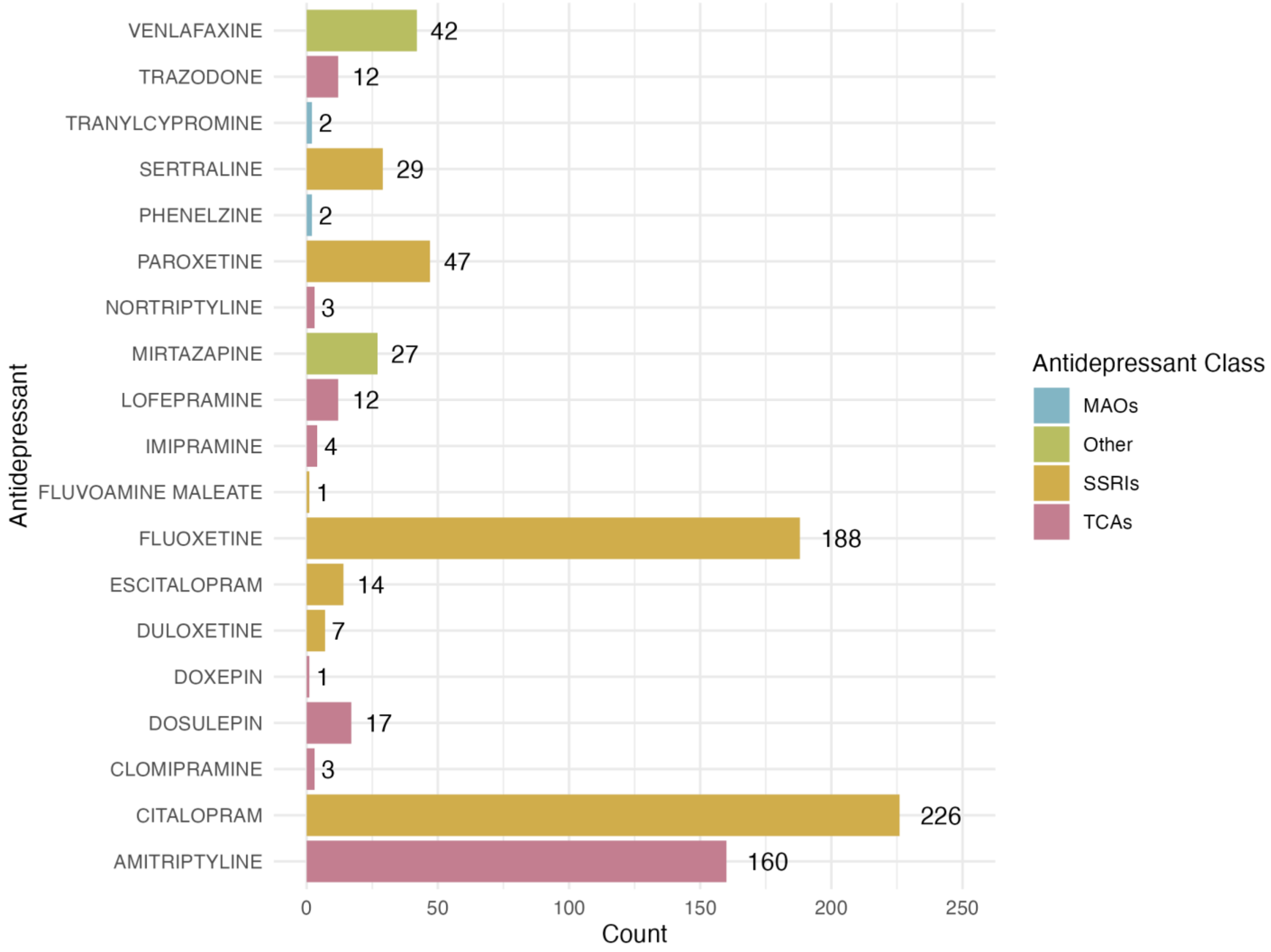

Supplementary Fig. 48: Counts of antidepressants which are self-reported in the version 2 of self-report questionnaire.

Self-reported antidepressant medications named by participants using free-text medication reporting (version 2 self-report questionnaire) ($n_{unexposed}$ = 9127, $n_{exposed}$ = 797). Note the self-reported phenotype is comprised of those who report an antidepressant in version 2 (displayed here), or report to taking antidepressants via a Yes/No checkbox in version 5.

Self-report and prescription-derived antidepressant exposure phenotypes

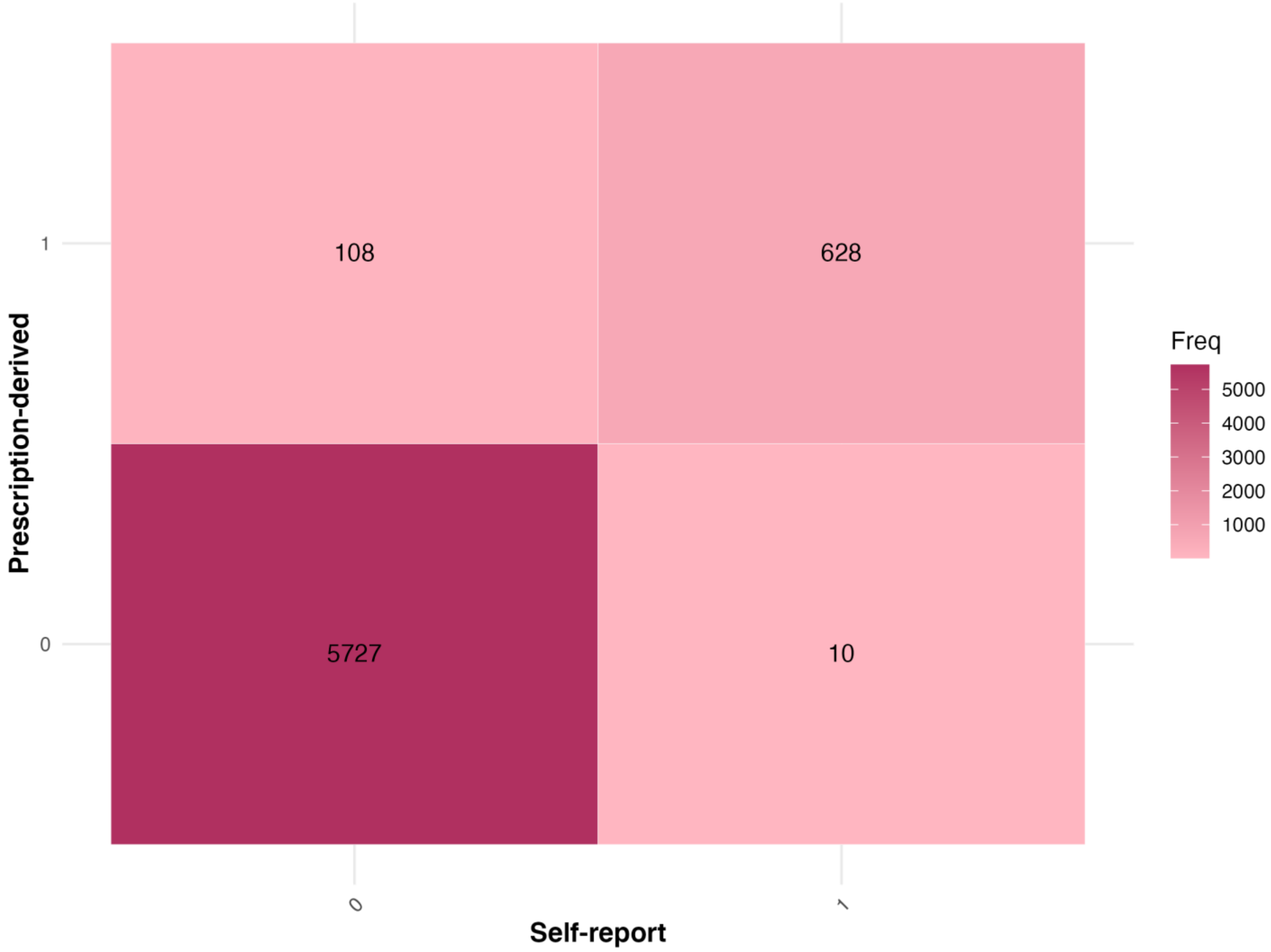

Supplementary Fig. 49: Overlap of the self-report and prescription-derived antidepressant exposure measures.

Comparison of self-report and prescription-derived measures of antidepressant exposure in those present in both samples (N = 6,473). Coding: 0 = Antidepressant-unexposed, 1 = Antidepressant-exposed. Boxes are colored by the number of individuals.

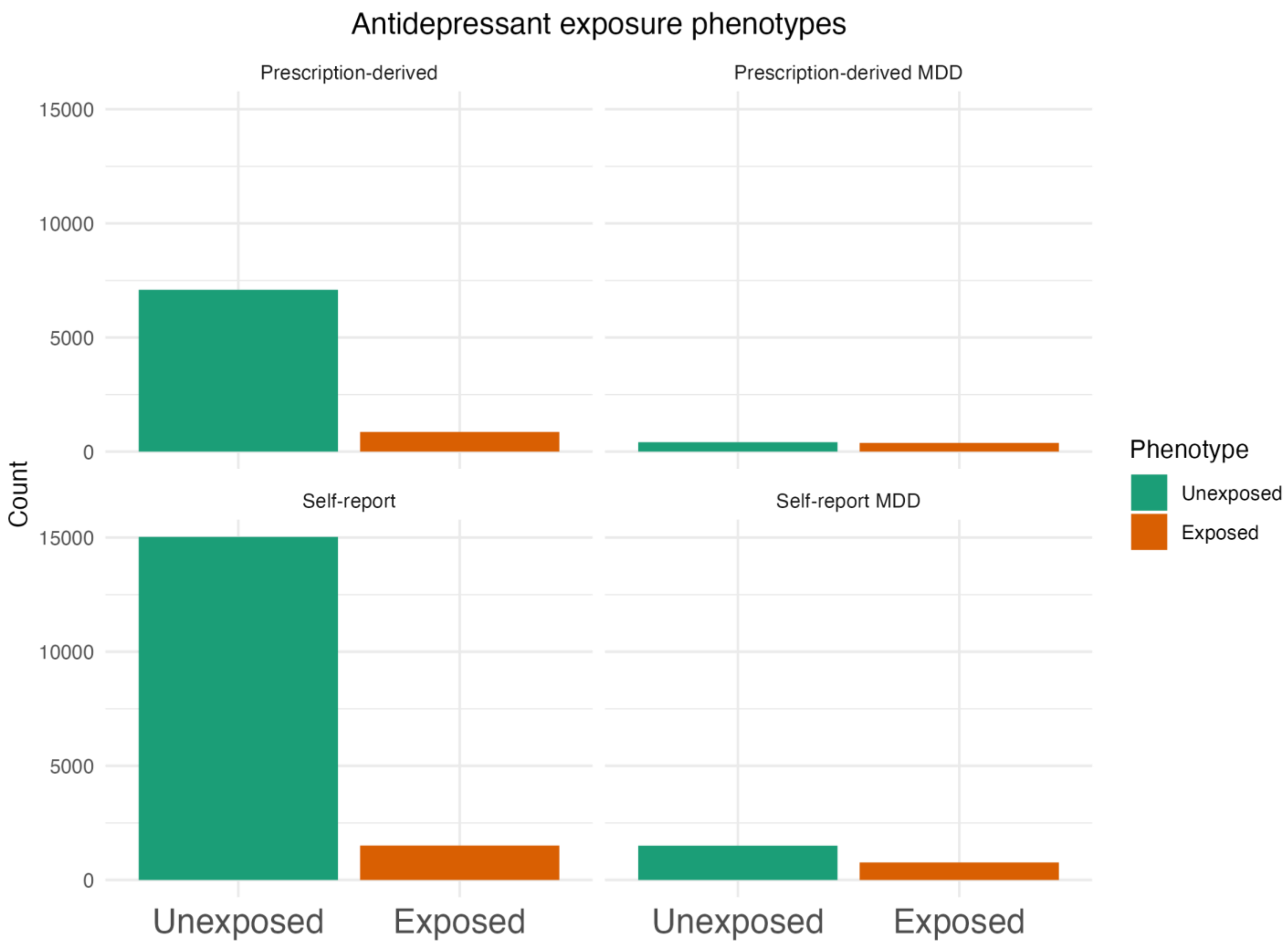

Supplementary Fig. 50: The antidepressant exposure phenotypes used in the methylome-wide association studies.

Antidepressant exposure phenotypes used in the MWAS analysis. Left Panel: Sample used in the discovery MWAS analysis (Self-report: $n_{exposed}$=1508, $n_{unexposed}$=15,023, Prescription-derived: $n_{exposed}$= 861 , $n_{unexposed}$=7,090). Right Panel, Samples filtered to those with a Major Depression Disorder (MDD) lifetime status only ascertained by the Structured Clinical Interview for DSM-IV Non-Patient Version (SCID) (Self-report: $n_{exposed}$=766, $n_{unexposed}$=1,502, Prescription-derived: n~exposed = 380, $n_{unexposed}$=412). This MDD-subgroup was used to investigate the potential confounding effect of MDD status on antidepressant exposure.

Supplementary Tables

Supplementary Table 1: Significant CpG ~ prescription-derived antidepressant exposure associations. The CpGs found to be significantly associated with prescription-derived antidepressant exposure using a Mixed-linear-model Omics-based Analysis (MOA) model (N = 7,951). Significance was assessed using the p-value threshold 9.42x10-8, as recommended for case-control MWAS analyses. Other traits reported significantly associated with the CpG in the EWAS catalog in studies (n > 1000), accessed on 17/03/2024.

Probe	Chr	bp	b	se	p	UCSC_RefGene_Name	Relation_to_Island	UCSC_RefGene_Group	Other traits associated with the CpG
cg01964004	2	74,196,572	0.026	0.0047	3.0e-08	DGUOK-AS1	OpenSea	Body	Incident Type 2 Diabetes (10.1101/2023.01.10.23284387) : Prevalent Chronic Pain (Self-report) (10.1101/2023.01.10.23284387) : Prevalent Ischemic Heart Disease (Self-report) (10.1101/2023.01.10.23284387) : Prevalent Type 2 Diabetes (Self-report) (10.1101/2023.01.10.23284387)
cg04173586	19	2,167,496	0.035	0.0048	7.0e-13	DOT1L	S_Shore	Body	Alcohol consumption (31789449) : COVID-19 Severity (Hospitalisation) (10.1101/2023.01.10.23284387) : Sex (26500701) : age (33450751) : alzheimers disease braak stage (33257653) : estimated glomerular filtration rate (eGFR) (33931109) : schizophrenia (33646943)
cg04315689	2	74,198,896	0.019	0.0035	3.9e-08	DGUOK-AS1	OpenSea	Body	Incident Type 2 Diabetes (10.1101/2023.01.10.23284387) : Prevalent Chronic Kidney Disease (Estimated) (10.1101/2023.01.10.23284387) : Prevalent Chronic Pain (Self-report) (10.1101/2023.01.10.23284387) : Prevalent Type 2 Diabetes (Self-report) (10.1101/2023.01.10.23284387)
cg26277237	9	631,910	0.027	0.0044	6.1e-10	KANK1	OpenSea	5'UTR	Prevalent Chronic Kidney Disease (Estimated) (10.1101/2023.01.10.23284387) : Prevalent Type 2 Diabetes (Self-report) (10.1101/2023.01.10.23284387) : estimated glomerular filtration rate (eGFR) (33931109)

Supplementary Table 2: Significant CpG ~ self-report antidepressant exposure associations in those with lifetime Major Depressive Disorder. The CpG found to be significantly associated with antidepressant exposure (self-report) in those with MDD (MDD-only analysis) using a Mixed-linear-model Omics-based Analysis (MOA) model (N = 2,268). Significance was assessed using the p-value threshold 9.42x10-8, as recommended for case-control MWAS analyses. There were no other traits reported significantly associated with the CpG in the EWAS catalog in studies (n > 1000), accessed on 17/03/2024.

Probe	Chr	bp	b	se	p	UCSC_RefGene_Name	UCSC_RefGene_Group
cg08527546	11	9,595,200	0.0501102	0.00909281	0.0000000356876	WEE1	TSS1500

Supplementary Table 3: Significant CpG ~ self-reported antidepressant exposure associations in Females only. CpGs found to be significantly associated with self-reported antidepressant exposure in Females only using a Mixed-linear-model Omics-based Analysis (MOA) model (N = 9,710). Significance was assessed using the p-value threshold 9.42x10-8, as recommended for case-control MWAS analyses.

Probe	Chr	bp	b	se	p	UCSC_RefGene_Name	UCSC_RefGene_Group
cg26277237	9	631,910	0.030	0.0047	1.8e-10	KANK1	5'UTR
cg02183564	7	76,874,892	0.023	0.0040	4.3e-09	CCDC146	Body

Supplementary Table 4: Self-report genes-SynGO annotations. Genes in the self-report gene-list which were annotated to a SynGO term.

Gene	HGNC Symbol	GO term ID	GO term name	SynGO annotation ID
EXOC2	HGNC:24968	GO:0090128	regulation of synapse maturation (GO:0090128)	164
EXOC2	HGNC:24968	GO:0099641	anterograde axonal protein transport (GO:0099641)	3655
PTPRN2	HGNC:9677	GO:0030285	integral component of synaptic vesicle membrane (GO:0030285)	1229
PTPRN2	HGNC:9677	GO:0098793	presynapse (GO:0098793)	3946
RAB7A	HGNC:9788	GO:0098993	anchored component of synaptic vesicle membrane (GO:0098993)	204
SLC6A1	HGNC:11042	GO:0098810	neurotransmitter reuptake (GO:0098810)	762
SLC6A1	HGNC:11042	GO:0099056	integral component of presynaptic membrane (GO:0099056)	764
SLC6A1	HGNC:11042	GO:0099055	integral component of postsynaptic membrane (GO:0099055)	767
SLC6A1	HGNC:11042	GO:0099056	integral component of presynaptic membrane (GO:0099056)	1903
SLC6A1	HGNC:11042	GO:0098810	neurotransmitter reuptake (GO:0098810)	1912
SYT11	HGNC:19239	GO:0098794	postsynapse (GO:0098794)	2562
SYT11	HGNC:19239	GO:0030285	integral component of synaptic vesicle membrane (GO:0030285)	2565
SYT11	HGNC:19239	GO:0099059	integral component of presynaptic active zone membrane (GO:0099059)	2566
SYT11	HGNC:19239	GO:1900242	regulation of synaptic vesicle endocytosis (GO:1900242)	3785
TRIO	HGNC:12303	GO:0099170	postsynaptic modulation of chemical synaptic transmission (GO:0099170)	2844
TRIO	HGNC:12303	GO:0098794	postsynapse (GO:0098794)	2849
TRIO	HGNC:12303	GO:0048786	presynaptic active zone (GO:0048786)	2922
TRIO	HGNC:12303	GO:0050804	modulation of chemical synaptic transmission (GO:0050804)	3948

Supplementary Table 5: Prescription-derived genes-SynGO annotations. Genes in the prescription-derived gene-list which were annotated to a SynGO term.

Gene	HGNC Symbol	GO term ID	GO term name	SynGO annotation ID
AKT1	HGNC:391	GO:0099175	regulation of postsynapse organization (GO:0099175)	3073
ALK	HGNC:427	GO:0099540	trans-synaptic signaling by neuropeptide (GO:0099540)	3247
ALK	HGNC:427	GO:0098794	postsynapse (GO:0098794)	3792
CHRNE	HGNC:1966	GO:1904315	transmitter-gated ion channel activity involved in regulation of postsynaptic membrane potential (GO:1904315)	1978
CHRNE	HGNC:1966	GO:1904315	transmitter-gated ion channel activity involved in regulation of postsynaptic membrane potential (GO:1904315)	1980
CHRNE	HGNC:1966	GO:0099060	integral component of postsynaptic specialization membrane (GO:0099060)	2015
CHRNE	HGNC:1966	GO:0099060	integral component of postsynaptic specialization membrane (GO:0099060)	2016
DGKI	HGNC:2855	GO:0099147	extrinsic component of postsynaptic density membrane (GO:0099147)	2194
DGKI	HGNC:2855	GO:0098891	extrinsic component of presynaptic active zone membrane (GO:0098891)	2373
DGKI	HGNC:2855	GO:0008021	synaptic vesicle (GO:0008021)	2374
DGKI	HGNC:2855	GO:0099171	presynaptic modulation of chemical synaptic transmission (GO:0099171)	2401
GABBR1	HGNC:4070	GO:0099056	integral component of presynaptic membrane (GO:0099056)	1251
GABBR1	HGNC:4070	GO:0150047	G protein-coupled neurotransmitter receptor activity involved in regulation of presynaptic membrane potential (GO:0150047)	1339
GABBR1	HGNC:4070	GO:0099579	G protein-coupled neurotransmitter receptor activity involved in regulation of postsynaptic membrane potential (GO:0099579)	1587
GABBR1	HGNC:4070	GO:0099056	integral component of presynaptic membrane (GO:0099056)	1588
GABBR1	HGNC:4070	GO:0099055	integral component of postsynaptic membrane (GO:0099055)	1678
GABBR1	HGNC:4070	GO:0099060	integral component of postsynaptic specialization membrane (GO:0099060)	2174
GABBR1	HGNC:4070	GO:0099055	integral component of postsynaptic membrane (GO:0099055)	2175
GABBR1	HGNC:4070	GO:0099059	integral component of presynaptic active zone membrane (GO:0099059)	2176
GABBR1	HGNC:4070	GO:0099056	integral component of presynaptic membrane (GO:0099056)	2771
PTEN	HGNC:9588	GO:0099524	postsynaptic cytosol (GO:0099524)	497
SHISA6	HGNC:34491	GO:0099061	integral component of postsynaptic density membrane (GO:0099061)	2342
SHISA6	HGNC:34491	GO:0098970	postsynaptic neurotransmitter receptor diffusion trapping (GO:0098970)	2343
SHISA6	HGNC:34491	GO:0098962	regulation of postsynaptic neurotransmitter receptor activity (GO:0098962)	2350
SNX9	HGNC:14973	GO:0098793	presynapse (GO:0098793)	4110
SNX9	HGNC:14973	GO:1900242	regulation of synaptic vesicle endocytosis (GO:1900242)	4111
STAU2	HGNC:11371	GO:0098964	anterograde dendritic transport of messenger ribonucleoprotein complex (GO:0098964)	3029

Supplementary Table 6: Correlation between DNA methylation and time in treatment. Two-tailed Spearman correlation statistics on the time in treatment and methylation at significant probes identified in the self-report and prescription-derived MWAS (n = 863). Source refers to which MWAS analysis the probe was significantly associated with antidepressant exposure. Probes with a significant correlation between the time in treatment and methylation are highlighted in yellow.

Probe	S_Statistic	Spearman_Rho	P_Value	source
cg01964004	100,530,494	0.061536763	0.07078576	Prescription only
cg02183564	105,536,131	0.014808590	0.66398116	Self report only
cg03222540	102,008,007	0.047744017	0.16111371	Self report only
cg04173586	105,678,970	0.013475179	0.69261954	Both
cg04315689	107,954,839	-0.007770317	0.81969280	Both
cg08907118	106,186,221	0.008739933	0.79765391	Self report only
cg15071067	98,001,369	0.085146423	0.01234051	Self report only
cg26277237	97,821,811	0.086822616	0.01071991	Both

Supplementary Table 7: Antidepressant exposure and Antidepressant exposure MPS associations. Associations for individual external cohorts using either a generalised linear model (FOR2107 and ALSPAC), a generalised linear mixed model (SHIP-Trend, LBC1936, MARS-UniDep, STRADL and E-Risk) and generalised estimation equation (FTC and NTR) models. 95% Confidence Intervals are calculated as the effect estimate +/- 1.96*standard error. The log-likelihood of the full model (incl MPS) and the null model (excl MPS) were used to calculate nagelkerke's R2.

Cohort	Estimate	SE	Z	P	OR	highCIOR	lowCIOR	loglik_null	loglik_MRS	nagelkerke
ALSPAC	0.170	0.16	1.090	2.8e-01	1.19	1.61	0.873	-165.50	-164.90	0.004360
ERISK	0.340	0.19	1.790	7.4e-02	1.41	2.05	0.968	-159.20	-157.60	0.011200
FOR2107	0.350	0.10	3.450	5.6e-04	1.42	1.73	1.160	-347.90	-341.70	0.028500
FTC	0.160	0.13	1.190	2.3e-01	1.17	1.51	0.904	-316.80	-312.40	0.016500
LBC1936	0.230	0.16	1.430	1.5e-01	1.26	1.72	0.918	-174.70	-170.30	0.030300
MARS	0.260	0.13	2.040	4.2e-02	1.30	1.68	1.010	-197.60	-195.50	0.018800
NTR	-0.031	0.11	-0.278	7.8e-01	0.97	1.21	0.780	-391.80	-391.80	0.000111
SHIP	0.160	0.24	0.681	5.0e-01	1.18	1.88	0.738	-76.35	-76.12	0.003530
STRADL	0.150	0.16	0.922	3.6e-01	1.16	1.60	0.845	-151.10	-150.70	0.003530

Supplementary Table 8: Antidepressant exposure ~ Methylation profile score meta-analysis. Effect estimates (TE) and p-values of antidepressant exposure ~ Antidepressant exposure MPS association for individual external cohorts using either a generalised linear model (FOR2107 and ALSPAC), a generalised linear mixed model (SHIP-Trend, LBC1936, MARS-UniDep, STRADL and E-Risk) and generalised estimation equation (FTC and NTR) models. 95% Confidence Intervals are calculated as the effect estimate +/- 1.96*standard error. The weight of each study in the random-effects meta-analysis (w.random) of antidepressant exposure ~ antidepressant exposure MPS is also shown.

Cohort	TE	seTE	zval	pval	lower CI	upper CI	w.common	w.random	w.fixed
ALSPAC	0.170	0.16	1.090	2.8e-01	-0.1350	0.4754	41.18	41.18	41.18
ERISK	0.340	0.19	1.790	7.4e-02	-0.0329	0.7165	27.37	27.37	27.37
FOR2107	0.350	0.10	3.450	5.6e-04	0.1510	0.5472	97.73	97.73	97.73
FTC	0.160	0.13	1.190	2.3e-01	-0.1010	0.4139	57.94	57.94	57.94
LBC1936	0.230	0.16	1.430	1.5e-01	-0.0857	0.5424	38.95	38.95	38.95
MARS	0.260	0.13	2.040	4.2e-02	0.0101	0.5169	59.83	59.83	59.83
NTR	-0.031	0.11	-0.278	7.8e-01	-0.2480	0.1867	81.20	81.20	81.20
SHIP	0.160	0.24	0.681	5.0e-01	-0.3040	0.6289	17.64	17.64	17.64
STRADL	0.150	0.16	0.922	3.6e-01	-0.1690	0.4682	37.90	37.90	37.90

Supplementary Table 9: Antidepressants used to measure prescription-derived antidepressant exposure. British National Formulary (BNF) Chapter '040303' medications which were used to index antidepressant prescriptions in Generation Scotland. The matching anatomical therapeutic classification (ATC) code is also given.

BNF Medications	BNF code	ATC code
Tricyclic and related antidepressant drugs		
Amitriptyline hydrochloride (0403010B0)	0403010B0	N06AA09
Amoxapine (0403010C0)	0403010C0	N06AA17
Clomipramine hydrochloride (0403010F0)	0403010F0	N06AA04
Dosulepin hydrochloride (0403010J0)	0403010J0	N06AA16
Doxepin (0403010L0)	0403010L0	N06AA12
Imipramine hydrochloride (0403010N0)	0403010N0	N06AA02
Lofepramine hydrochloride (0403010R0)	0403010R0	N06AA07
Maprotiline hydrochloride (0403010S0)	0403010S0	N06AA21
Mianserin hydrochloride (0403010T0)	0403010T0	N06AX03
Nortriptyline (0403010V0)	0403010V0	N06AA10
Trazodone hydrochloride (0403010X0)	0403010X0	N06AX05
Trimipramine maleate (0403010Y0)	0403010Y0	N06AA06
Monoamine oxidase inhibitors		
Isocarboxazid (0403020H0)	0403020H0	N06AF01
Moclobemide (0403020K0)	0403020K0	N06AG02
Phenelzine sulfate (0403020M0)	0403020M0	N06AF03
Tranylcypromine sulfate (0403020Q0)	0403020Q0	NO6AF04
Selective serotonin re-uptake inhibitors		
Citalopram hydrobromide (0403030D0)	0403030D0	N06AB04
Citalopram hydrochloride (0403030Z0)	0403030Z0	N06AB04
Duloxetine Hydrochloride (old) (0403030Y0)	0403030Y0	N06AX21
Escitalopram (0403030X0)	0403030X0	N06AB10
Fluoxetine hydrochloride (0403030E0)	0403030E0	N06AB03
Fluvoxamine maleate (0403030L0)	0403030L0	N06AB08
Paroxetine hydrochloride (0403030P0)	0403030P0	N06AB05
Sertraline hydrochloride (0403030Q0)	0403030Q0	N06AB06
Other antidepressant drugs		
Agomelatine (0403040Z0)	0403040Z0	N06AX22
Duloxetine hydrochloride (0403040Y0)	0403040Y0	N06AX21
Flupentixol hydrochloride (0403040F0)	0403040F0	NO5AF01
Mirtazapine (0403040X0)	0403040X0	N06AX11
Nefazodone hydrochloride (0403040T0)	0403040T0	N06AX06
Oxitriptan (0403040R0)	0403040R0	N06AX01
Reboxetine (0403040U0)	0403040U0	N06AX18
Tryptophan (0403040S0)	0403040S0	N06AX02
Venlafaxine (0403040W0)	0403040W0	N06AX16
Vortioxetine (0403040AB)	0403040AB	N06AX26

Supplementary Table 10: Antidepressant prescriptions before parsing instructions. Number of antidepressant medications recorded by PIS in Generation Scotland participants following parsing prescription instructions and filtering out uninformative (e.g NA) and titrating (e.g 1 tab a day for a week then stop) prescription dosages.

Prescription item approved name	N prescriptions	N individuals	Formulation	BNF paragraph code	BNF paragraph name
CITALOPRAM	43,890	2,641	Tablets, Drops	403,030	Selective serotonin re-uptake inhibitors
AMITRIPTYLINE	39,860	3,769	Tablets, Solution	403,010	Tricyclic and related antidepressant drugs
FLUOXETINE	26,087	1,813	Capsules, Solution, Tablets	403,030	Selective serotonin re-uptake inhibitors
SERTRALINE	17,357	1,307	Tablets	403,030	Selective serotonin re-uptake inhibitors
MIRTAZAPINE	12,271	1,021	Tablets, Solution	403,040	Other antidepressant drugs
VENLAFAXINE	11,898	462	Tablets, Capsules	403,040	Other antidepressant drugs
DULOXETINE	4,181	302	Capsules	403,040	Other antidepressant drugs
PAROXETINE	4,083	155	Tablets, Liquid	403,030	Selective serotonin re-uptake inhibitors
TRAZODONE HYDROCHLORIDE	3,770	276	Capsules, Tablets, Liquid	403,010	Tricyclic and related antidepressant drugs
ESCITALOPRAM	2,991	190	Tablets	403,030	Selective serotonin re-uptake inhibitors
DOSULEPIN HYDROCHLORIDE	2,356	103	Tablets, Capsules	403,010	Tricyclic and related antidepressant drugs
NORTRIPTYLINE	1,268	229	Tablets	403,010	Tricyclic and related antidepressant drugs
CLOMIPRAMINE HYDROCHLORIDE	1,134	53	Capsules, Tablets	403,010	Tricyclic and related antidepressant drugs
LOFEPRAMINE	1,045	95	Tablets	403,010	Tricyclic and related antidepressant drugs
IMIPRAMINE HYDROCHLORIDE	792	64	Tablets	403,010	Tricyclic and related antidepressant drugs
MOCLOBEMIDE	287	14	Tablets	403,020	Monoamine-oxidase inhibitors (maois)
PHENELZINE	271	9	Tablets	403,020	Monoamine-oxidase inhibitors (maois)
TRANYLCYPROMINE	228	3	Tablets	403,020	Monoamine-oxidase inhibitors (maois)
DOXEPIN	226	21	Capsules	403,010	Tricyclic and related antidepressant drugs
REBOXETINE	221	11	Tablets	403,040	Other antidepressant drugs
TRYPTOPHAN	83	6	Tablets	403,040	Other antidepressant drugs
FLUVOXAMINE MALEATE	72	2	Tablets	403,030	Selective serotonin re-uptake inhibitors
TRIMIPRAMINE	51	3	Capsules, Tablets	403,010	Tricyclic and related antidepressant drugs
AGOMELATINE	30	4	Tablets	403,040	Other antidepressant drugs
MIANSERIN HYDROCHLORIDE	2	1	Tablets	403,010	Tricyclic and related antidepressant drugs
Total	174,454	12,554			

Supplementary Table 11: Antidepressant prescriptions after parsing instructions. Number of antidepressant medications recorded by PIS in Generation Scotland participants following parsing prescription instructions and filtering out uninformative (e.g NA) and titrating (e.g 1 tab a day for a week then stop) prescription dosages.

Prescription item approved name	N prescriptions	N individuals	Formulation	BNF paragraph code	BNF paragraph name
CITALOPRAM	43,434	2,624	Drops, Tablets	403,030	Selective serotonin re-uptake inhibitors
AMITRIPTYLINE	37,728	3,553	Solution, Tablets	403,010	Tricyclic and related antidepressant drugs
FLUOXETINE	25,918	1,812	Solution, Capsules, Tablets	403,030	Selective serotonin re-uptake inhibitors
SERTRALINE	17,033	1,281	Tablets	403,030	Selective serotonin re-uptake inhibitors
MIRTAZAPINE	11,953	989	Solution, Tablets	403,040	Other antidepressant drugs
VENLAFAXINE	11,258	448	Tablets, Capsules	403,040	Other antidepressant drugs
PAROXETINE	4,043	154	Liquid, Tablets	403,030	Selective serotonin re-uptake inhibitors
DULOXETINE	4,042	288	Capsules	403,040	Other antidepressant drugs
TRAZODONE HYDROCHLORIDE	3,561	264	Liquid, Capsules, Tablets	403,010	Tricyclic and related antidepressant drugs
ESCITALOPRAM	2,951	189	Tablets	403,030	Selective serotonin re-uptake inhibitors
DOSULEPIN HYDROCHLORIDE	2,320	99	Tablets, Capsules	403,010	Tricyclic and related antidepressant drugs
NORTRIPTYLINE	1,106	205	Tablets	403,010	Tricyclic and related antidepressant drugs
CLOMIPRAMINE HYDROCHLORIDE	1,030	49	Capsules, Tablets	403,010	Tricyclic and related antidepressant drugs
LOFEPRAMINE	877	84	Tablets	403,010	Tricyclic and related antidepressant drugs
IMIPRAMINE HYDROCHLORIDE	634	54	Tablets	403,010	Tricyclic and related antidepressant drugs
MOCLOBEMIDE	234	14	Tablets	403,020	Monoamine-oxidase inhibitors (maois)
REBOXETINE	208	11	Tablets	403,040	Other antidepressant drugs
DOXEPIN	184	21	Capsules	403,010	Tricyclic and related antidepressant drugs
PHENELZINE	180	9	Tablets	403,020	Monoamine-oxidase inhibitors (maois)
TRANYLCYPROMINE	86	3	Tablets	403,020	Monoamine-oxidase inhibitors (maois)
FLUVOXAMINE MALEATE	71	2	Tablets	403,030	Selective serotonin re-uptake inhibitors
TRIMIPRAMINE	49	3	Capsules, Tablets	403,010	Tricyclic and related antidepressant drugs
TRYPTOPHAN	39	3	Tablets	403,040	Other antidepressant drugs
AGOMELATINE	29	4	Tablets	403,040	Other antidepressant drugs
MIANSERIN HYDROCHLORIDE	2	1	Tablets	403,010	Tricyclic and related antidepressant drugs
Total	168,970	12,164			

Supplementary Table 12: Self-report antidepressant exposure. Antidepressant medications named in the version 2 of the self-report questionnaire, which allowed participants to insert free-text of medications they were taking.

Antidepressant	N
AMITRIPTYLINE	160
CITALOPRAM	226
CLOMIPRAMINE	3
DOSULEPIN	17
DOXEPIN	1
DULOXETINE	7
ESCITALOPRAM	14
FLUOXETINE	188
FLUVOAMINE MALEATE	1
IMIPRAMINE	4
LOFEPRAMINE	12
MIRTAZAPINE	27
NORTRIPTYLINE	3
PAROXETINE	47
PHENELZINE	2
SERTRALINE	29
TRANYLCYPROMINE	2
TRAZODONE	12
VENLAFAXINE	42
Total	797

Supplementary Methods

Study Population: Generation Scotland

Protocol Paper: https://doi.org/10.1093/ije/dys084[9]

In depth information regarding the recruitment and assessment of participants in Generation Scotland (GS) can be found elsewhere[10]. In brief, participants over the age of 18 years were randomly recruited through general practice NHS surgeries within Scotland from 2006 to 2011. Participants filled out a pre-clinical questionnaire, and subsequently attended an extensive in-person clinic where physical measurements, biological sampling, and a psychiatric assessment was performed. Participants were informed that the nature of the study was to investigate the health of the Scottish population and gave consent before any sampling took place.

Methylation data: DNAm quality control

DNAm profiling of the GS samples was carried out by the Genetics Core Laboratory at the Edinburgh Clinical Research Facility, Edinburgh, Scotland. Quality control and normalisation steps were applied to each wave separately. Quality control in the first wave was performed using *shinyMethyl* R package[11]. Outliers were removed based on visual inspection of the log median intensity of methylated vs unmethylated signals. Samples were removed for which DNAm-predicted sex did not match recorded sex. The following were removed using the *wateRmelon* R package[12]: i) samples where >1% of CpGs have detection p-value > 0.05, ii) probes with a beadcount < 3 in > 5% of samples; and iii) probes for which >0.5% of samples had a detection p value > 0.05. Proportion of white blood cells were estimated using the houseman method implemented by the *minfi* R package[13], using the reference data from Reinius et al, (2012)[14]. Quality control steps on wave 2-4 were performed by the R package *meffil*[15] and *shinyMethyl*. Dye bias and background correction was performed using *meffil* and the *'noob'* method[16] to exclude: samples with strong dye bias and/or issues with bisulphite conversion, samples with outlying median methylated signal intensity and samples where DNAm predicted sex did not match recorded sex. *Meffil* was used to remove samples with > 0.5% of CpG sites with detection p value > 0.01, alongside probes which had a beadcount < 3 in > 5% of samples and/or had a detection p-value >0.01 in > 1% of samples. Proportion of white blood cells were estimated using the houseman method implemented by *minfi*, using the reference data from Reinius et al, (2012)[14]. Multi-dimensional scaling (MDS) was performed, and visual inspection identified any outlying samples which were removed. Following quality control, probes on the Y and X chromosome were removed, alongside those which are predicted to bind poorly according to either Zhou et al, (2017)[17] or McCartney et al, (2016)[18]. Following quality control, there were 5,087, 459, 4,450 and 8,873 individuals within set 1, 2, 3, and 4 respectively. The sets were combined and dasen normalisation was performed across all individuals, using *wateRmelon*. The dasen-normalised beta-values were converted to M-values using the *'beta2M()'* function in the *lumi* R package[19].

Antidepressant exposure phenotypes

Prescription-derived antidepressant exposure

Consent was obtained from 23,603 participants for their data in GS to be linked to the National Health Service (NHS) records, through their Community Health Index (CHI) number. An individual's CHI number is routinely used by NHS Scotland for various procedures, including prescription events[20]. Prescription data for these individuals was obtained from the Scottish National Prescribing Information System (PIS)[21], which gives access to individual prescribing and dispensing events in a community setting since 2009. Further information about the PIS database can be found elsewhere[21]. There was a total of 2,841,726 prescription events for 21,318 individuals, with dispensing dates spanning April 2009 to January 2017. A prescription data-entry had extensive information including the medication name, formulation, quantity, strength, BNF codes, dispense data, paid date, and free-text prescriber instructions.

Antidepressant prescriptions selected included serotonin reuptake inhibitors (SSRIs) (Fluoxetine, Sertraline, Paroxetine, Citalopram, Escitalopram and Fluvoxamine Maleate), monoamine oxidase inhibitors (MAOs) (Moclobemide, Phenelzine, Tranylcypromine), tricyclic antidepressants (TCAs) (Amitriptyline, Clomipramine, Dosulepin, Doxepin, Imipramine, Lofepramine, Mianserin, Nortriptyline, Trazodone, Trimipramine) and other antidepressants (Agomelatine, Mirtazapine, Reboxetine, Tryptophan, Venlafaxine). Exact numbers of each antidepressant are shown in Figure **36**. The vast majority (n = 173,818) of the prescriptions were for tablets or capsules, and the rest were in liquid forms (n = 636).

Ambiguous prescription instructions

Prescriptions with ambiguous or dosage-irrelevant instructions, e.g 'as directed', 'as instructed', 'taken in the morning' (n = 9216) were identified by the regex expressions: *^$_, _^(to be taken\s*|(for\s*)?use\s*|take\s*)?as (dir|directed|advised|discussed).*(to be taken\s*|(for\s*)?use\s*|take\s*)?$* and *^non formulary please choose citalopram instead$*. For the prescriptions in capsule and tablet form ($N_{prescriptions}$ =9187), the instruction was assumed to be a default of 1 tablet a day. For the solutions (n = 636), ambiguous prescriptions (n = 48) were imputed to be the same as the most common prescription instruction given to the individual in the previous year, if there were no previous prescriptions (n = 6), then the prescription was discarded from the dataset.

Additionally, prescriptions which had instructions for titrating or reducing dosages e.g 'take 2 a day for a week, then 1, then stop' ($n_{tablets}$ = 5430, 3.12% $n_{liquids}$ = 48, 7.55%) were discarded from the analysis.

Multiple prescriptions on the same day

2, 604 people received multiple (1+) prescriptions on the same dispense date ($N_{prescriptions}$ = 37, 817). We theorised that this could be due to two possibilities:

- **Scenario 1) Same continuous treatment**: each prescription is for the continuation of the same treatment and are being collected ahead of time.

- **Scenario 2) Different simultaneous treatment**: each prescription aligns to a separate treatment and are to be taken simultaneously.

We distinguish between each scenario using a decision tree, illustrated in Supplementary Figure **44**. In brief, prescriptions assumed to be for the **scenario 1** (same continuous treatment) if a) prescriptions are for the same medication and the same dosage, or b) medications for the same medication and different dosages but the combined dosage is over the maximum recommended dosage as we theorise that this would not be prescribed to be taken simultaneously. If **scenario 1** is met, then the multiple prescriptions are merged together to one prescription event (i.e 2 prescriptions, each with 28 tablets, would be merged to be one prescription with 56 tablets) ($N_{prescriptions}$= 10, 016). Prescriptions are assumed to be for **scenario 2** (separate simultaneous treatments) if a) the prescriptions are for different medications, or b) the prescriptions are for the same medication and different dosages, but the combined dosage is under the maximum recommended dosage. If it is determined to be **scenario 2)**, then we treat each prescription as independent and do not merge the prescription events ($N_{prescriptions}$= 27, 801).

A summary of processing of the prescription data is shown in Supplementary Figure **46**

Treatment periods

For each prescription, the prescription duration (PD) would be calculated as the fraction of the total dosage by the daily dosage (imputed from the prescription instructions). This is then extended by 10% to account for non-perfect adherance to medication.

$$PD = 1.1\left(\frac{Dose_{Total}}{Dose_{Daily}}\right) \qquad (1)$$

The expected end date ($E(ED)$) of a prescription is then calculated by adding the estimated prescription duration (PD) onto the Dispense Date (DD) of the prescription, assuming individuals take the first dosage on the day of dispensation.

$$E(ED) = DD + PD \qquad (2)$$

Each individual had all their antidepressant prescriptions assigned to a treatment 'period'. The first prescription on record ($Pres_1$), was assigned to Period 1 (Per_1). For the remaining n prescriptions, $Pres_2$ to $Pres_n$, the period assigned to each prescription was determined by the following criteria:

If the prescription's dispense date was earlier than the expected end date of the previous prescription ($DD_{Pres_n} \leq E(ED_{n-1})$), then it would be assigned to the same period as the previous prescription.

$$Period(Pres_n) = Period(Pres_{n-1}) \qquad (3)$$

The $E(ED)_n$ would then be calculated again according to Equation (2).

Otherwise, in instances where the dispense date is after the expected end date of the previous prescription ($DD_{P_n} > E(ED_{n-1})$), then it would be assigned to a new period of treatment.

$$Period(Pres_n) = Period(Pres_{n-1} + 1) \qquad (4)$$

Each treatment period is defined as starting at the dispense date of the first prescription within the treatment period and ending at the expected end date of the last prescription within the period.

Self-report phenotypes

Self-reported antidepressant exposure was assessed using two different questionnaires. The first version (n = 9, 924) enabled participants to free text any medications they were taking. The second version (n = 13, 675) had the question "Are you regularly taking any of the following medications" with a yes/no checkbox next to a "Antidepressants" option. Classification of antidepressant exposure was done using both; those who named an antidepressant medication from the British National Formulary Chapter "040303" (which roughly equates to Anatomical Therapeutic Chemical (ATC) subclass 'NO6A') and those who checked 'yes' for "Antidepressants" ($N_{exposed}$ = 1, 508) were classed as antidepressant-exposed. Those who did not name any antidepressants or checked 'no' were classed as antidepressant-unexposed ($N_{unexposed}$ = 15, 023). The medications which were selected as antidepressants in the 1st version of the self-report medications

questionnaire included tricyclic antidepressants (Amitriptyline, Amoxapine, Clomipramine, Dosulepin, Doxepin, Imipramine, Lofepramine, Maprotiline, Mianserin, Nortriptyline, Trazodone, Trimipramine), monoamine-oxidase inhibitors (Isocarboxazid, Moclobemide, Phenelzine, Tranylcypromine), selective serotonin reuptake inhibitors (Citalopram, Duloxetine, Escitalopram, Fluoxetine, Fluvoxamine Maleate, Paroxetine, Sertraline), and other antidepressants (Agomelatine, Duloxetine, Flupentixol, Mirtazapine, Nefazodone, Oxitriptan, Reboxetine, Tryptophan, Venlafaxine, Vortioxetine).

Major Depressive Disorder

Major Depressive Disorder (MDD) was confirmed using the Structured Clinical Interview for DSM-IV Non-Patient Version (SCID)[22]. Those who answered 'yes' to an initial two screening questions, completed a more extensive interview about the nature of the MDD (i.e a current or historical episode of MDD, the age of onset and the number of episodes). However, for this analysis, the phenotype was binary-coded: participants who answered no for the initial screen, or those who did not meet the MDD criteria following the extensive interview were assigned as controls, and those who answered yes and met the criteria for either single episode or recurrent MDD were assigned as cases. Participants who fulfilled criteria for bipolar disorder in the SCID assessment were discarded (n = 75). All interviewers received multiple group training sessions in the administration of the SCID throughout the study and were blinded to the diagnostic status of any relatives in the study. Furthermore, the SCID phenotype within GS was found to have a high inter rater-reliability for the identification of MDD cases[23].

Permutation-based enrichment analysis; Netherlands Study of Anxiety and Depression

Protocol paper: https://doi.org/10.1002/mpr.256[24]

Cohort Description

The Netherlands Study of Depression and Anxiety is a naturalistic ongoing longitudinal cohort study which aims to investigate the long-term course and consequences of depression and anxiety in the Netherlands. Recruitment took place in the general population across various health care settings (community, primary care and specialised mental health care) for individuals between 18 and 65 years of age. The recruited sample consists of 1,701 individuals with current diagnosis of depression and/or anxiety, 907 individuals with lifetime diagnoses or at risk due to family history of subthreshold symptoms and 373 healthy controls. A four-hour baseline assessment was conducted and included written questionnaires, interviews, a medical examination, cognitive tasks, blood and saliva sampling and intensive screening regarding mental health outcomes. Detailed assessments were then repeated after one, two, four and eight years of follow-up.

Ethics Approval: The study protocol was approved centrally by the Ethical Review Board of the VU University Medical Centre and subsequently by local review boards of each participating centre. After full verbal and written information about the study, written informed consent was obtained from all participants at the start of baseline assessment.

Depression status and antidepressant exposure phenotypes

Major Depressive Disorder (MDD) DSM-IV diagnoses were obtained using the Composite International Diagnostic Interview (CIDI). The present study only included persons who fulfilled the 6-month recency MDD diagnosis. During the assessment, participants were asked about current medication. The assignment of drugs to the antidepressant treatment group was based on drug container inspection of all medications used in the past month, classified according to the World Health Organization Anatomical Therapeutic Chemical (ATC) classification. We included three classes of antidepressants: selective serotonin reuptake inhibitors (SSR: ATC: N06AD), tricyclic antidepressants (TCA: ATC: N06AA) and other antidepressants (ATC: N05AX). Those who named any single medication were classed as antidepressant-exposed (n = 398), and those who did not use a single antidepressant medication were defined as antidepressant unexposed (n = 414).

DNA methylation: Methyl-CG binding domain sequencing (MBD-Seq) assay

DNA methylation was assayed at baseline from whole-blood samples from 812 MDD patients using an optimized protocol for methyl-CG binding domain sequencing (MBD-Seq). Elsewhere we summarized key features of optimized MBD-seq using empirical data[25]. Critically, comparisons with whole genome bisulfite sequencing (WGB-Seq) showed that our optimized MBD-Seq protocol achieves near complete coverage of all methylation sites in blood[27]. First, genomic DNA samples were sonicated to 150bp using a Covaris S2 ultra-sonicator. Next, DNA fragments with methylated CpGs (mCpG) were enriched using components of the MethylMinerTM Kit (Invitrogen) via affinity purification with the methyl-CG domain protein (MBD2). For each capture reaction, 15uL of prepared MBD-beads was incubated with 1.5ug of fragmented DNA for 1 hour at

room temperature on an orbital shaker at 650rpm, and then washed three times with 1x Bind-Wash Buffer. The bound methylated fragments were recovered in three pooled elutions of 500mM NaCI buffer and purified by ethanol precipitation. The MBD enriched fractions were used to generate indexed libraries with the TruSeq Nano DNA HT Library Prep Kit (Illumina). Libraries were size-selected using SPRI beads to obtain a mean insert size of 150bp. The libraries were then pooled and sequenced on the NextSeq 500 using a 75-bp single-end configuration and High-Output v2 chemistry (Illumina). Reads were aligned (build hg19/GRCh37) with Bowtie2[28] using a seed-and-extend approach combined with local alignment while allowing for gaps. Specifically, we used a 20bp seed with zero mismatches. Rather than considering the entire read, local alignment was used to improve sensitivity by finding the maximum similarity score between the reference sequence and a substring of the extension that may be "trimmed" at both ends. Gaps were allowed to account for small indels.

Quality control of MBD-Seq data

Data was further processed using *RaMWAS*, an R/Bioconductor pipeline for large-scale analyses of methylation data from enrichment platforms[29]. The code and software are available from Bioconductor (https://bioconductor.org/packages/release/bioc/html/ramwas.html) as well as GitHub (https://github.com/andreyshabalin/). Reads aligning to loci without CpGs (non-CpGs) represent "noise" caused by, for example, imperfect enrichment leading to non-methylated fragments being sequenced. We use a threshold of 0.05 for non-CpG/CpG coverage ratio to remove samples with high-noise levels, leaving an average of 0.010 (SD = 0.005) in the remaining samples. Samples were also excluded if the sequence variants called from the methylation data did not match the genotype information, as this indicates that perhaps a sample swap or sample contamination occurred. Multidimensional outliers were identified, using principal components of the methylation dataset using the function *"pcout"* from the *mvoutliers* R package[30] and excluded. The mean number of reads per subject was 59,758,680 and the average alignment rate was 99.1%. Quality control steps were performed for both multi- and duplicate-reads. For multi-reads, i.e., when a read aligns to multiple locations of the genome, Bowtie2 selected the alignment with a highest alignment score. In the case where Bowtie2 encounters a set of equally good alignment it uses a pseudo-random number to select one primary alignment. Duplicate-reads, i.e., multiple identical reads with the same start and end location, are more likely to occur when sequencing enriched genomic fractions. Therefore, we allow up to three duplicate-reads at each genomic position. In the instance where there are > 3 duplicate reads, read count is reset to 1, implicitly assuming that excess reads are tagging a single clonal fragment. This results in an average of 48,287,403 reads per sample (81.6% of all reads).

Identifying CpGs and quantifying DNAm

We combined the reference genome sequence (hg19/GRCh37) with common SNPs calculated on the European super population from the 1000 Genomes (Phase 3). CpGs which were 1) created by SNPs with minor allele frequency < 1%, 2) in loci prone to alignment errors (identified using RaMWAS in-silico alignment experiment)[31] and 3) with an average coverage of < 0.1 or having 0 coverage in > 90% of samples were removed. This left a total of 26,269,235 CpGs for analysis. CpGs methylation were quantified using RaMWAS in

a three-step process. First, a non-parametric approach is used to estimate the fragment size distribution from the sequencing data using isolated CpGs[32]. This distribution is then used to calculate the probability that a sequenced fragment will cover the CpG under consideration. The CpG score is then calculated by taking the sum of probabilities for all fragments aligning within proximity of the CpG.

Enrichment analysis

We tested whether top findings from the case-control methylome-wide association studies (MWAS) in Generation Scotland (GS) were also more likely to be among the top findings of the MWAS of antidepressant exposure in NESDA MDD cases. For this purpose, we created for both MWASs a yes/no variable indicating whether sites were in the top X percentage, using three different thresholds (0.1, 0.5 and 1%). As CpG methylation tends to be highly correlated over short distances[25], we allowed for a flank of 150bp on either site of the CpG in the more sparse GS methylation array data when mapping results from the two MWASs. Next, we cross-classified the two yes/no variables resulting in a 2 by 2 table. The tests for enrichment in overlap were performed using Cramér's V (sometimes referred to as Cramér's phi). We chose Cramér's V over a variety of other association statistics for 2 by 2 table based on a simulations study showing it resulted in the most powerful test. For the analyses we used circular permutations[33] to account for having correlated methylation levels between CpG sites. Previous studies have shown that circular permutations properly control the type I error even if correlations between methylation sites were > 0.99[34]. A total of 100,000 permutations were performed were performed with the help of our R package shiftR (https://cran.r-project.org/web/packages/shiftR/index.html) that performs permutations through fast bitwise operations. P values were calculated as the proportion of permutations that yielded a value equal of greater that Cramér's V observed in the analysis of the observed data. Three thresholds (0.1, 0.5 and 1%) were used to define top findings in the MWASs. We corrected for this "multiple testing" by using the same thresholds in the permutations and then selected the most significant result for each permutation.

Acknowledgements

The NESDA study is supported by the Geestkracht program of the Netherlands Organization for Health Research and Development (Zon-Mw, grant number 10–000-1002) and the participating institutions (VU University Medical Center, Leiden University Medical Center, University Medical Center Groningen. The current methylation project was supported by grant R01MH099110 from the National Institute of Mental Health. The sponsors had no role in the design and conduct of the study; collection, management, analysis, and interpretation of the data; preparation, review, or approval of the manuscript; or decision to submit the manuscript for publication.

External Cohorts: Methylation Profile Score

The association between antidepressant exposure and antidepressant exposure MPS was assessed using generalised linear mixed models, generalised linear models and generalised estimation equations, depending on the cohort's population structure (i.e., twin studies vs. unrelated participants) and DNAm pre-processing. In depth information about each cohort, the DNAm preprocessing, the calculation of the MPS and the association model (antidepressant-exposure ~ MPS) is detailed below, and summarised in Supplementary Tables 7-9.

Finnish Twin Cohort (FTC)

Protocol papers:

Older Finnish Cohort: https://doi.org/10.1017/thg.2019.54[35]

FinnTwin12: https://doi.org/10.1017/thg.2019.83[36]

FinnTwin16: https://doi.org/10.1017/thg.2019.106[37]

Cohort Description

The Finnish Twin Cohort comprises of three longitudinal sub cohorts: The Older Finnish Twin Cohort[35] (Old), established in the 1970s, and the FinnTwin12 (FT12)[36] and FinnTwin16 (FT16)[37] cohorts established in the 1990s to identify genetic and environmental precursors of various health-related behaviours and diseases. The Old cohort consists of Finnish same-sex monozygotic and dizygotic twin pairs born before 1958 (n = 13, 888 pairs) with extensive data collection including behavioural and lifestyle traits over multiple waves in 1975, 1981, 1990 and 2011. The younger cohorts, FT12 and FT16 are longitudinal studies of five consecutive birth cohorts (born in 1983-1987, n = 2, 700 pairs and 1975-1979, n = 2, 800 pairs, respectively) of Finnish monozygotic and dizygotic twin pairs. Participants have completed multiple surveys on behavioural and lifestyle traits at five rounds of follow up beginning in adolescence through to adulthood (FT16 at age 16, 17, 18.5, 24, 34; FT12 at age 12, 14, 17.5, 22, 35). In addition to the 4-5 questionnaire waves, the twins have participated in several intensive on-site studies including interviews, clinical measures and tests, and blood sampling. FT16 intensive study in 2001-2006 included pairs discordant and concordant for alcohol use. These pairs were interviewed and sampled at the mean age of 26, with 599 twins providing DNA[38]. Wave 4 of FT12 included intensive study for randomly selected pairs during 2006-2009, with 1260 twins completing the interviews and clinical protocol, and providing DNA at the mean age of 222. Old cohort twin pairs discordant and concordant for blood pressure were included in a study on epigenetics of essential hypertension (EH-Epi) in 2012-2015[39]. Altogether 428 twins at the mean age of 62 participated in the clinical protocol and provided DNA samples.

Ethical Approval: Participants were given information on the study procedures and of freedom to participate or to decline at any point in both oral and written form. Informed consent was obtained upon the contact with the study subjects before new questionnaire information was collected, and when clinical investigations were

undertaken with sampling of biological material. Ethics approvals have been granted for multiple studies concerning the FTC twins by the ethics committees of Helsinki University Central Hospital (113/E3/2001, 249/E5/2001, 346/E0/05, 270/13/03/01/2008, and 154/13/03/00/2011) with the last one on the transfer of biological samples to the THL Biobank in 2018 (HU51179912017).

Antidepressant exposure phenotype

In the younger cohorts, FT12 and FT16, antidepressant exposure was recorded using the psychiatric Semi-Structured Assessment for Genetics of Alcohol (SSAGA) psychiatric interviews, which included question on current medication use ("Have you used antidepressants within the last 30 days?") in a selected subsample of participants, as described above. Antidepressant exposure in the Old cohort was ascertained in a subsample of 447 twins participating in the EH-Epi study using on-site interview, which asked, "What medications are you currently using?" For all the twin cohorts (FT12, FT16 and Old) the medications were coded by Anatomical Therapeutic Chemical (ATC codes), and subclasses of N06A were considered antidepressants. Those who named any antidepressant medication were defined as antidepressant-exposed and were defined as those who did not name a single antidepressant medication. In total, there were 84 exposed (41 FT12, 18 FT16 and 25 Old cohort) and 1594 unexposed (805 FT12, 478 FT16 and 311 Old cohort) with good quality DNAm data generated from the blood samples collected at the same visit with the interview on AD use.

DNAm preprocessing

DNAm was profiled from whole blood samples using the Infinium HumanMethylation450K ('450K array') or EPIC ('EPIC array') BeadChip Kit (Illumina, San Diego, CA, USA). Altogether 336 Old cohort (Average Age = 62.1), 764 FT12 (Average Age = 22.4), and 215 FT16 (Average Age = 26.1) cohort individuals were measured on the 450K array, and 82 FT12 (Average age = 22.3) and 281 FT16 (Average age = 26.3) cohort individuals were measured on the EPIC array. The following quality control pipeline was used for both the 450K and EPIC DNAm data. The DNAm data were pre-processed in the R package *meffil*[15]. Bad quality samples were excluded based on the following criteria: i) sex mismatch, ii) median methylation vs. unmethylated signal > 3 standard deviations (SD), iii) failed control probe metrics and if > 20% of probes per sample had iv) detection p value > 0.05 and v) bead number < 3. To remove technical variation between the samples, functional normalisation including the control probe principal components was performed, followed by bad quality probe removal: i) probes with detection p value > 0.05 in more than 20% samples, ii) bead number < 3 in more than 20% samples, iii) sex chromosome probes and iv) cross-reactive and ambiguously mapped probes as well as probes on polymorphic CpGs[17]. Beta Mixture quantile (BMIQ) normalisation implemented in the R package *wateRmelon*[12] was then performed to adjust for type 2 probe bias. The data pre-processing resulted in 390,304 and 765,385 probes in 450K (n = 1315) and EPIC data (n = 363), respectively. Cell-type fractions were estimated using *EpiDISH* R package[41], running Robust Partial Correlations with a reference for 7 main immune cell subtypes: neutrophils, basophils, monocytes, B-cells, NK-cells, CD4+ and CD8+ T-cells[42]. Lymphocyte cell proportions were then calculated as the aggregate of the estimated CD4+ T, CD8+ T, NK and B cell proportions. Methylation beta values were transformed to M-values using *'logit2()'* function in the *minfi*[13] R package. The M-values were then standardised using Z-score normalisation using the *scale()* function in R.

Methylation Profile Score

Following the quality control protocol, there were 183 CpGs and 211 CpGs present on the 450K (all the 3 cohorts) and EPIC array (FT12 & FT16) respectively, out of the 212 CpGs which make up the methylation profile score (MPS). There was no missingness in the CpGs deriving the MPS from either array. The MPS for each individual was derived as a weighted sum of the 183 or 211 available CpGs, depending on which array DNAm was profiled.

Antidepressant exposure ~ MPS association model

The association between antidepressant exposure and the antidepressant exposure MPS was tested under a generalised estimation equation (GEE) model, using the *'glmgee()'* function from the *glmtoolbox* R package[43], with antidepressant exposure as the outcome. The model included the following predictors: antidepressant exposure MPS, age at blood sampling, sex, the estimated monocyte cell proportions, estimated lymphocyte (aggregate of CD4T, CD8T, B cells and NK cells) cell proportions and M values at the AHRR probe ('cg05575921') to proxy for smoking status. The following settings were used: family=binomial, 50 maximum number of iterations, and the 'exchangeable' option to account for correlation structure within families and within persons.

```
assoc_mod <- glmgee(as.factor(antidep)~ scale(AD_MRS) + scale(age) + scale(Mono) +
                    scale(lymphocytes) + scale(cg05575921) + as.factor(sex_coded),
                    data = MRS_covs_pheno,
                    id = familyID,
                    family = binomial,
                    corstr = "Exchangeable")
```

Funding and Acknowledgment

Phenotype and genotype data collection in FT12 and FT16 studies of the Finnish twin cohort has been supported by the Wellcome Trust Sanger Institute, the Broad Institute, ENGAGE – European Network for Genetic and Genomic Epidemiology, FP7-HEALTH-F4-2007, grant agreement number 201413, National Institute of Alcohol Abuse and Alcoholism (grants AA-12502, AA-00145, and AA-09203 to R J Rose; AA15416 and K02AA018755 to D M Dick; R01AA015416 to J Salvatore) and the Academy of Finland (grants 100499, 205585, 118555, 141054, 264146, 308248, 265240, 263278 to J Kaprio, 328685, 307339, 297908 and 251316 to M Ollikainen, and Centre of Excellence in Complex Disease Genetics grants 312073, 336823, and 352792 to J Kaprio), NIH/NHLBI (grant HL104125 to X Wang), and Sigrid Juselius Foundation to M Ollikainen and J Kaprio.

Study of Health in Pomerania (SHIP-Trend)

Protocol paper:https://doi.org/10.1093/ije/dyac034[44]

Cohort Description

The Study of Health in Pomerania (SHIP) is a population-based cohort (N ~ 12,728, Trend = 4,420 and Start = 4,308, Next ~ 4,000) with the aims of assessing the prevalence and risk factors underlying several clinical and subclinical disorders within Pomerania, Western Germany[44,45]. For this analysis, data from the SHIP-Trend cohort was used. Comprehensive data collection of this cohort, including in-depth interviews, imaging, and biological sampling for 4,420 subjects was conducted between September 2008 and September 2012.

Ethical Approval Participants provided written informed consent before any assessment and/or sampling took place. The Ethics Committee of the University Medicine Greifswald, Germany provided ethical approval for the study (BB 39/08).

Antidepressant exposure phenotype

All participants were asked to bring any medications they were currently taking (in the last 7 days) to their in-clinic interview. Medications were coded using Anatomical Therapeutic Chemical (ATC) codes, subclasses of N06A were considered antidepressants. Those who brought any antidepressant medication were defined as antidepressant-exposed, and those were defined to those who did not bring any antidepressant medication were defined as antidepressant-unexposed. In total, there were 21 exposed and 474 unexposed with quality DNAm data.

DNAm preprocessing

DNAm was profiled from blood samples at baseline for a subsample of SHIP-Trend participants, randomly selected based on the availability of multi-OMICS data, using the Illumina HumanMethylation EPIC BeadChip array (n=508). Samples excluded type II diabetes and were enriched for prevalent myocardial infarction. The samples were taken between 07:00 AM and 04:00 PM. Serum aliquots were prepared for immediate analysis and for storage at -80 °C in the Integrated Research Biobank (Liconic, Liechtenstein) and the DNA samples were processed at the Helmholtz Zentrum München. The CPACOR workflow was followed for the preparation and normalization of the array data using the software package R (www.r-project.org), with the array idat files being processed using the *minfi* R package[13]. Probes that had a detection p-value above background (sum of per-array methylated and unmethylated intensity values, based p-value ≥ 1x10-16) were set to missing. Methylation beta values were calculated as proportion of methylated intensity value on the sum of methylated+unmethylated+100 intensities. During steps such as bisulfite conversion, hybridization and extension, arrays were removed following observation of technical problems (±4 SD outside control probe intensity mean). Additionally arrays with a mismatch of recorded sex and methylation-predicted sex were excluded. Additionally, arrays with a call rate < 5% were removed from the analysis. A total of 495 samples with methylation data on 865,859 sites was available for subsequent analyses following quality control. Methylation β values were transformed into M-values by first setting values of zero to half the value of the

smallest non-zero β of the CpG site over all participants, and then using Mi=log2(βi/(1-βi)). The M-values were then standardised using Z-score normalisation using the *'scale()'* function in R.

Methylation Profile Score

After adhering to the quality control protocols, all 212 CpGs were available for the calculation of the MPS, which was derived as a weighted sum of the CpGs. There were 145 individuals with data available for all 212 CpGs, with others having varying degrees of missingness in the data. This was mainly confined to 3 CpGs (cg13569486, cg26192826 and cg15897613). The MPS for each individual was derived as a weighted sum of the CpGs. In the case that an individual had a missing value for a CpG(s), the probe(s) were excluded from the MPS (i.e., given a weight of 0).

Antidepressant exposure ~ MPS association model

The association between the antidepressant exposure and antidepressant exposure MPS was tested using a generalised linear mixed effects model with a logistic link function, using the *'glmer()'* function from the *lme4* R package[46], with antidepressant exposure as the outcome. The model included the following predictors with a fixed effect: antidepressant exposure MPS, age at blood sampling, sex, white blood cell count, percentage of monocytes and lymphocytes, M values at the AHRR probe ('cg05575921') to proxy for smoking status, and the top 3 principal components of the control probe intensities (obtained by the CPACOR workflow). Each of those variables (except sex) were standardised using Z-score normalisation. Additionally, array processing batch was included as a random effect.

```
Generalized linear mixed model fit by maximum likelihood (Laplace Approximation) ['glmerM
od']
Family: binomial  ( logit )
Formula: as.factor(antidep) ~ scale(AD_MRS) + scale(AGE_SHIP_T0) + scale(mo_pct_e) + scal
e(ly_pct_e) + scale(AHRR) + as.factor(SEX_SHIP_T0) + scale(PC1_cp) + scale(PC2_cp) + scal
e(PC3_cp)  + (1 | Batch) + scale(wbc)
```

Funding and Acknowledgement

SHIP is part of the Community Medicine Research net of the University of Greifswald which is funded by the Federal Ministry of Education and Research (01ZZ9603, 01ZZ0103, and 01ZZ0403), the Ministry of Cultural Affairs and the Social Ministry of the Federal State of Mecklenburg-West Pomerania. DNA methylation data have been supported by the DZHK (grant 81X3400104). The University of Greifswald is a member of the Caché Campus program of the InterSystems GmbH.

FOR2107

Study Population

Protocol paper: https://doi.org/10.1007/s00406-018-0943-x[47]

FOR2107 is a research programme including both a human cohort (target n = 2500) and animal models, which broadly aims to establish the neurobiological mechanisms of genetic and environmental risk factors for affective disorders[47]. The human cohort consists of individuals from various groups, those diagnosed with MDD, those diagnosed with bipolar disorder, those diagnosed with schizophrenia, those diagnosed with schizoaffective disorder, and healthy controls (HC) with and without genetic/environmental risk factors. Participants undergo an in-person extensive deep-phenotyping (MR imaging, clinical course, neuropsychology, personality, risk/protective factors, biomaterial: blood, stool, urine, hair, saliva) at baseline and again at a follow-up appointment two years later. Baseline data collection started in November 2014 and is ongoing.

Ethical Approval Participants provided written consent before any assessment and/or sampling took place. The ethics committees of the Medical Faculties, University of Marburg (AZ: 07/14) and University of Münster (2014-422-b-S) provided ethical approval for the study.

Antidepressant exposure phenotype

During the in-person assessment participants were asked about their current medication. The assignment of drugs to the antidepressant treatment group largely aligns with the ATC coding and BNF coding (Supplementary Table 1). Individuals diagnosed with bipolar or schizophrenia, or schizoaffective disorder were excluded from the analysis. Those who named any single antidepressant medication were defined as antidepressant-exposed and those who did not name a single antidepressant medication nor any single neuroleptic medication were defined as antidepressant-unexposed, based on the described classification. In total, there were 165 exposed (all having a lifetime diagnosis of MDD) and 493 unexposed (thereof, 167 with a lifetime diagnosis of MDD and 326 HC) with high-quality DNAm data.

DNAm preprocessing

DNAm profiling and preprocessing were conducted within the larger FOR2107 DNAm sample (n=881), of which the sample eligible for the current study constitutes a subset. DNAm was profiled from peripheral whole blood samples using the Illumina Infinium MethylationEPIC BeadChip v1.0. Quality control was performed using the *minfi*[13] and *ewastools*[48] R packages. Samples which had a call rate < 98 %, sex mismatch, or mismatch in SNP fingerprints and those with technical issues detected from the Illumina control probes were excluded from the analysis. After stratified quantile normalisation, methylation probes which had a call rate < 98%, SNPs inside the probe body, a non-CpG type or a non-autosomal genomic position were excluded. Cross-reactive or polymorphic probes with an allele frequency of >5 % in the European population (n = 52, 541) were excluded based on procedure outlined by McCartney et al (2016)[18]. The sample was further filtered for the availability of high-quality genome-wide genotyping data (Illumina Infinium PyschArray-24 BeadChip) to examine relatedness within the sample, and relatives with pi-hat > 0.125 were excluded. After filtering the sample for

the eligibility criteria of the antidepressant exposure phenotype defined above, 764, 556 CpGs and 658 individuals remained for analysis. M values were extracted for downstream analysis using the *'getM()'* function implemented in *minfi* and were then standardised using Z-score normalisation using the *'scale()'* function in R. Cell proportions (CD4T, CD8T, NK cells, B cells, Granulocytes and Monocytes) were estimated using the Houseman approach as implemented in *minfi*[13]. Lymphocyte cell proportions were then calculated as the sum of the estimated CD4T, CD8T, NK and B cell proportions.

Methylation Profile Score

After adhering to the quality control protocols, all 212 CpGs were available for the calculation of the MPS, which was derived as a weighted sum of the CpGs. There was no missingness in any of the CpGs included in the score.

Antidepressant exposure ~ MPS association model

The association between the antidepressant exposure and antidepressant exposure MPS was tested using a generalised linear model with a logistic link function, using the *'glm()'* function from the *stats* R package[49], with antidepressant exposure as the outcome. The model included the following predictors with a fixed effect: antidepressant exposure MPS, age at blood sampling, sex, estimated lymphocyte cell proportions (aggregate of CD4T, CD8T, B cells and NK cells), estimated monocyte cell proportions, M values at the AHRR probe ('cg05575921') to proxy for smoking status, and the top 10 genetic principal components.

```
glm(formula = as.factor(antidep) ~ scale(AD_MRS) + scale(age) +
    scale(Mono) + scale(lymphocytes) + scale(cg05575921) + as.factor(sex_coded) +
    scale(C1) + scale(C2) + scale(C3) + scale(C4) + scale(C5) +
    scale(C6) + scale(C7) + scale(C8) + scale(C9) + scale(C10),
    family = "binomial", data = MRS_covs_pheno)
```

Funding and Acknowledgement

The German multicenter consortium "Neurobiology of Affective Disorders. A translational perspective on brain structure and function" is funded by the German Research Foundation (Research Unit FOR2107). Principal investigators are Tilo Kircher (speaker FOR2107, DFG grant numbers KI588/14-1, KI588/14-2, KI588/20-1, KI588/22-1, KI 588/15-1, KI 588/17-1), Udo Dannlowski (co-speaker FOR2107; DA1151/5-1, DA1151/5-2, DA1151/6-1), Axel Krug (KR3822/5-1, KR3822/7-2), Igor Nenadic (NE2254/1-2, NE2254/2-1, NE2254/3-1, NE2254/4-1), Carsten Konrad (KO4291/3-1), Marcella Rietschel (RI 908/11-1, RI 908/11-2), Markus Nöthen (NO 246/10-1, NO 246/10-2), Stephanie Witt (WI 3439/3-1, WI 3439/3-2). Tilo Kircher received unrestricted educational grants from Servier, Janssen, Recordati, Aristo, Otsuka, neuraxpharm. We are deeply indebted to all study participants and staff. A list of acknowledgments can be found here: www.for2107.de/acknowledgements.

Netherlands Twin Register

Protocol paper: https://doi.org/10.1017/thg.2019.93[50]

Study population

The Netherlands Twin Register is a population-based cohort of over 200,000 people from across the Netherlands. It consists of twin-families, i.e. twins, their parents, spouses and siblings aged between 0 and 99 years at recruitment, and started around 1987 with new–born twins and adolescent and adult twins. Full details have been reported previously[50]. DNA was collected from buccal cells and whole blood as part of multiple projects. For the current paper, we analysed DNA methylation measured in whole blood collected in the NTR-Biobank study[53]. Good quality whole blood DNA methylation data were available for 3087 samples from 3055 individuals, including monozygotic and dizygotic twins, parents of twins, siblings of twins and spouses of twins. For 32 individuals, longitudinal methylation data were available (two time points, mean range=5.2 year). The current analysis included individuals with information on antidepressant medication use and covariates (N = 3, 004 samples).

Ethical Approval Informed consent was obtained from all participants. The study was approved by the Central Ethics Committee on Research Involving Human Subjects of the VU university Medical Centre, Amsterdam, an Institutional Review Board certified by the U.S Office of Human Research Protections (IRB number IRB00002991 under Federal-wide Assurance-FWA00017598; IRB/institute codes, NTR 03-180).

Antidepressant exposure phenotype

At blood draw, participants were asked about all current medication use and for all medicines the dose, brand and chemical names were recorded directly from the medication packaging. Following the Anatomical Therapeutic Chemical (ATC) classification system, subclasses of N06A were considered anti-depressants. Antidepressant medication use was coded as 1 (antidepressant-exposed) / 0 (antidepressant-unexposed). There were 87 recorded exposed and 2,917 unexposed with quality DNAm data.

DNAm preprocessing

DNA methylation was assessed with the Infinium HumanMethylation450 BeadChip Kit (Illumina, San Diego, CA, USA) by the Human Genotyping facility (HugeF) of ErasmusMC, the Netherlands (http://www.glimdna.org/) as part of the Biobank-based Integrative Omics Study (BIOS) consortium[54]. DNA methylation measurements have been described previously[54]. Genomic DNA (500ng) from whole blood was bisulfite treated using the Zymo EZ DNA Methylation kit (Zymo Research Corp, Irvine, CA, USA), and 4 μl of bisulfite-converted DNA was measured on the Illumina 450k array following the manufacturer's protocol. A number of sample- and probe-level quality checks and sample identity checks were performed, as described in detail previously[53]. In short, sample-level QC was performed using R pacakge, *'MethylAid'*[55]. Probes were set to missing in a sample if they had an intensity value of exactly zero, or a detection p > .01, or a bead count of<3. After these steps, probes that failed based on the above criteria in >5% of the samples were excluded from all samples (only probes with a success rate ≥ 0.95 were retained). The following probes were also

removed: sex chromosomes, probes with a single nucleotide polymorphism (SNP) within the CpG site (at the C or G position) irrespective of minor allele frequency in the Genome of the Netherlands (GoNL) population, irrespective of minor allele frequency[56], and ambiguous mapping probes reported by Chen et al with an overlap of at least 47 bases per probe[40]. The methylation data were normalized with functional normalization[57].

Methylation Profile Score

After adhering to quality control protocols, all 212 CpGs were available for the calculation of the MPS, which was derived as a weighted sum of the CpGs. The rate of missingness per CpG ranged from 0% to 3.2% (average 0.4%). 71 CpGs had no missing values. In the case that an individual had a missing value for a CpG(s), the probe(s) were excluded from the MPS (i.e., given a weight of 0).

Antidepressant exposure ~ MPS association model

The association between antidepressant exposure and the MPS was assessed using a generalised linear mixed model, using the *'glmer()'* function from the *lme4* R package[46], with antidepressant exposure as the outcome. The model included the following predictors, antidepressant exposure MPS, sex, age at blood sampling, percentages of monocytes, eosinophils and neutrophils, HM450K array row, 96-wells bisulfite sample plate (dummy-coding), M values at the AHRR probe ('cg05575921') to proxy for smoking status and family ID as a random effect. The model was specified with the logit link function and 20 quadrature points in the adaptive Gaussian quadrature approximation for integrating over the random effect. The optimisation algorithm used was the Bound Optimisation by Quadratic Approximation (BOBYQA) with a maximum of 100,000 function evaluations.

```
assoc_mod_glmr2 <- glmer(as.factor(antidep) ~ scale(AD_MRS) + scale(age) + as.factor(sex)
+ scale(Mono_Perc) + scale(Eos_Perc) + scale(Neut_Perc) + as.numeric(Array_rownum) + as.f
actor(Sample_Plate_collapsed_fix) + scale(cg05575921) + (1|familynumber), data=MRS_covs_p
heno_ahhr, family=binomial(link = "logit"), nAGQ = 20,
          control = glmerControl(optimizer = "bobyqa", optCtrl = list(maxfun = 100000)))
```

Funding and Acknowledgements

We warmly thank all twin families of the Netherlands Twin Register who make this research possible. This work was supported by the Royal Dutch Academy for Arts and Science (KNAW) Academy Professor Award (PAH/6635) to DIB; the Netherlands Organization for Scientific Research (NWO 480-15-001/674) and Biobanking and Biomolecular Resources Research Infrastructure (BBMRI-NL: 184.021.007; 184.033.111).

Munich Antidepressant Response Study / Unipolar Depression Study

MARS protocol paper: https://doi.org/10.1016/j.jpsychires.2008.05.002[58]

UniDep protocol paper: https://doi.org/10.1093/hmg/ddl166[59]

Study population(s)

The Munich Antidepressant Response Signature (MARS) project[58] is a naturalistic longitudinal clinical study providing a sample of adult Caucasian inpatients (aged between 18 and 75 years) admitted to psychiatric hospitals in an acute episode of major depression (according to ICD-10) in southern Germany. Data was collected between 1995 and 2005. Further characterisation of the cohort and information on study protocol has been described elsewhere[58].

Ethical Approval: The study was approved by the local Ethics Committee of the Ludwig Maximilians University, Munich, Germany (318/00; 244/01) and carried out in accordance with the latest version of the Declaration of Helsinki. All participants provided written consent after the study protocol and potential risks were explained.

The Unipolar Depression study (UniDep)[59] is a cross-sectional case-control study in Germany. It consists of German in and outpatients (n = 1000, 67.4% female) with recurrent MDD from the Max-Planck-Institute of Psychiatry in Munich and psychiatric hospitals in Augsburg and Ingolstadt (located near Munich), with each hospital contributing a third of the patients. Patients were diagnosed according to the DSM-IV using the schedule for Clinical Assessment in Neuropsychiatry. Only Caucasian patients over 18 years of age with at least two moderate/severe depressive episodes were included. Those with the presence of manic or hypomanic episodes, mood incongruent psychotic symptoms, the presence of a lifetime diagnosis of intravenous drug abuse, and depressive symptoms which are secondary to a substance abuse disorder or due to medical illness or medication were excluded from the study. The mean age was 49.35 (±14.09 years). A control sample (n = 1029) was randomly selected from a Munich-based community sample, screened for the presence of anxiety or affective disorders using the Composite International Diagnostic Screener (CIDI), and matched for ethnicity, age (to 5-year intervals) and sex to those in the cases sample. Baseline data was collected between 2002 and 2004. Further characterisation of the cohort is described by Lucae et al (2006)[59].

Ethical Approval: The UniDep study was approved by the Bavarian State Medical Association (BLAEK) (01217) and written informed consent was obtained from all subjects.

Antidepressant exposure phenotype

For the MARS Depression cohort, baseline antidepressant exposure was assessed by asking patients about the antidepressants they had taken before admission to the hospital. During their stay at the hospital, information on the use of antidepressants was taken weekly from the patients clinical record. Assignment of drugs to the antidepressant group largely aligns with the ATC coding and BNF coding (Supplementary Table 1). The antidepressant exposure phenotype was taken from the antidepressant measure closest to the blood draw

date per individual within 14 days of the blood draw. The mean/median time between the self-report rating and the blood draw was a day and 0 days respectively.

In the UniDep cohort, which was designed as a case-control study for the identification of genes contributing to unipolar depression, antidepressant exposure was not assessed among patients. Therefore, from the UniDep study only controls who were screened for mental disorders and any psychopharmacological medication use, could be included in the present analysis. Those who named any single antidepressant medication were defined as antidepressant-exposed (MARS only), and those who did either not name an antidepressant medication (MARS) or were recruited as controls (without MDD) were defined as antidepressant-unexposed (MARS + UniDep). In total, there were 135 antidepressant-exposed (MARS) and 177 antidepressant unexposed (35 MARS, 142 UniDep) with high quality DNAm data.

DNAm preprocessing

For both cohorts, genomic DNA was extracted from whole blood samples using the Gentra Puregene Genomic DNA and run together on the same arrays. Samples were randomised with respect to case-control status, sex and age. Genomic DNA was bisulfite converted using the Zymo EZ-96 DNA Methylation Kit (Zymo Research) and DNA methylation levels were assessed for > 480, 000 CpG sits using the Infinium HumanMethylation450 BeadChip Kit (Illumina, San Diego, CA, USA). Quality control of DNAm data, including intensity read outs, normalisation, cell type composition estimation, Beta-value and M value calculation was performed using the Bioconductor R package *minfi* (version 1.10.2)[13]. Samples with outlying mean intensities and those with mismatch between DNAm-predicted and reported sex were excluded from the analysis. Probes present on the X and Y chromosomes and those with non-specific binding[40] were removed. Additionally, probes with a detection P-value > 0.01 in > 50% of the samples and those with a SNP located either at the interval at which the probe was design to hybridise or within 10bp from a SNP with a minor allele frequency > 0.01 (as reported in the 1000 Genomes Project)[60] were removed. Following these steps there was ~ 425, 000 CpGs available for further analysis. The data were then normalized with functional normalisation, using the *'FunNorm()'* function, an extension of quantile normalisation including in *minfi*. Visual inspection of the methylation principal components analysis plots was conducted to identify batch effects using the Bioconductor R package *shinyMethyl* (version 0.99.3)[11]. Identified batch effects (i.e., bisulfite conversion plate and plate position) were removed using the Empirical Bayes' method *'ComBat'*[61]. Batch-corrected M-values after *ComBat* were used for all further statistical analyses.

Methylation Profile Score

After adhering to the quality control protocols, 210 CpGs were available for the calculation of the MPS, which was derived as a weighted sum of the CpGs. There was no missingness in any of the CpGs included in the score.

Antidepressant exposure ~ MPS association model

The association between the antidepressant exposure and antidepressant exposure MPS was tested using a generalised linear model with a logistic link function, using the *'glm()'* function from the *stats* R package[49],

with antidepressant exposure as the outcome. The model included the following predictors with a fixed effect: antidepressant exposure MPS, age at blood sampling, sex, estimated lymphocyte cell proportions (aggregate of CD4T, CD8T, B cells and NK cells), estimated monocyte cell proportions, M values at the AHRR probe ('cg05575921') to proxy for smoking status, and the top 10 genetic principal components.

```
glm(formula = as.factor(antidep) ~ scale(AD_MRS) + scale(age) +
    scale(Mono) + scale(lymphocytes) + scale(cg05575921) + as.factor(sex_coded) +
    scale(C1) + scale(C2) + scale(C3) + scale(C4) + scale(C5) +
    scale(C6) + scale(C7) + scale(C8) + scale(C9) + scale(C10),
    family = "binomial", data = MRS_covs_pheno)
```

Funding and Acknowledgement

The MARS cohort was sponsored by the Max Planck Society. The UniDep cohort was funded by the Bavarian Ministry of Commerce and by the Federal Ministry of Education and Research in the framework of the National Genome Research Network, Foerderkennzeichen 01GS0481 and the Bavarian Ministry of Commerce. DNA methylation analysis of a subset of both cohorts was financed by ERA-NET NEURON.

We would like to thank all contributors to the research project including physicians, psychologists, study nurses, researchers and research assistants, and of course patients of the hospital of the Max Planck Institute of Psychiatry in Munich and psychiatric hospitals in Augsburg and Ingolstadt.

Lothian Birth Cohort 1936

Protocol paper: https://doi.org/10.1186/s13073-018-0585-7[62]

Cohort Description

The Lothian Birth Cohort 1936 (N = 1, 091) is a follow-up study of the Scottish Mental Surveys distributed to 11 year olds residing in Scotland in 1947, and is designed to examine the distribution and causes of cognitive aging across the human life course[63]. Participants took the survey at 11 years old and were recruited to LBC1936 at the mean age of 70 years. LBC1936 has conducted various waves of assessment, including a series of cognitive, clinical, physical, and social data alongside blood donations which have been used for multi-omics measurement. Initial baseline assessment was conducted in a single visit to the Wellcome Trust Clinical Research Facility at the Western General Hospital in Edinburgh between November 2006 and May 2007. Participants provided written consent before any assessment and/or sampling took place

Ethical Approval: Ethical approval was obtained from the Multicentre Research Ethics Committee for Scotland (baseline, MREC/01/0/56), the Lothian Research Ethics Committee (age 70, LREC/2003/2/29), and the Scotland A Research Ethics Committee (ages 73, 76, 79, 07/MRE00/58). All participants provided written informed consent.

Antidepressant exposure phenotype

At each wave of assessment, medication currently being taken by participants was recorded in a structured interview. Drugs were assigned to medication classes following the British National Formulary (BNF) paragraph code, with antidepressant codes beginning with '04030' (eTable 1). Those who named any single antidepressant medication were defined as antidepressant-exposed and those who did not name a single antidepressant medication were defined as antidepressant-unexposed. At wave 1, there were 46 exposed and 843 unexposed individuals with high quality DNAm data.

DNAm preprocessing

DNAm was profiled from whole blood samples at baseline (N = 1, 004) using HumanMethylation450 BeadChip Kit, Illumina, San Diego, CA, USA. Details on sample preparation and quality check have been reported previously[64]. Firstly, raw intensity data were background corrected and normalised using internal controls. Manual inspection of the array control probe signals was used to identify and remove low quality samples (e.g., samples with inadequate hybridization, bisulfite conversion, nucleotide extension or staining signal). Probes with a low detection rate ($p > 0.01$ for more than 5% of samples), low call rate ($p < 0.01$ for < 95% of probes) were removed. Samples whose predicted sex, based on XY probes, did not match reported sex were removed, along with those for whom SNP array genotypes were inconsistent with predicted genotypes derived from the methylation array's 65 'rs' control probes. After these quality control steps, 457, 047 autosomal probes remained from 895 individuals. M-values were calculated using the "beta2m" function from the *lumi* R package[19]. All M values were normalised using beta-mixture quantile normalisation[65].

Methylation Profile Score

After adhering to the quality control protocols, 206 CpGs were available for the calculation of the MPS, which was derived as a weighted sum of the CpGs. There was no missingness in any of the CpGs included in the score.

Antidepressant exposure ~ MPS association model

The association between the antidepressant exposure and the antidepressant exposure MPS was tested using a generalised linear mixed effects model with a logistic link function, using the *'glmer()'* function from the *lme4* R package[46], with antidepressant exposure as the outcome. The model included the following predictors with a fixed effect: antidepressant exposure MPS, age at blood sampling, sex, neutrophil cell count, lymphocyte cell count, eosinophil cell count, basophil cell count, M values at the AHRR probe (cg05575921) to proxy for smoking status and the top 4 genetic principal components. Additionally, array processing batch was included as a random effect, to account for additional technical factors.

```
Generalized linear mixed model fit by maximum likelihood (Laplace
  Approximation) [glmerMod]
 Family: binomial  ( logit )
Formula: as.factor(antidep) ~ scale(AD_MRS) + scale(age) + scale(neut) +
    scale(lymph) + scale(cg05575921) + scale(mono) + scale(eosin) +
    scale(baso) + as.factor(sex_coded) + scale(C1) + scale(C2) +
    scale(C3) + scale(C4) + (1 | array)
   Data: MRS_covs_pheno
```


Funding and Acknowledgement

The LBC1936 is supported by the Biotechnology and Biological Sciences Research Council, and the Economic and Social Research Council [BB/W008793/1] (which supports SEH), Age UK (Disconnected Mind project), the Medical Research Council [G0701120, G1001245, MR/M013111/1, MR/R024065/1], the Milton Damerel Trust, and the University of Edinburgh. SRC is supported by a Sir Henry Dale Fellowship jointly funded by the Wellcome Trust and the Royal Society (221890/Z/20/Z). Methylation typing of was supported by Centre for Cognitive Ageing and Cognitive Epidemiology (Pilot Fund award), Age UK, The Wellcome Trust Institutional Strategic Support Fund, The University of Edinburgh, and The University of Queensland.

Avon Longitudinal Study of Parents and Children

ALSPAC cohort protocol paper(s): https://doi.org/10.1093/ije/dys064[66], https://doi.org/10.1093/ije/dys066[67]
DNAm protocol paper (ARIES): https://doi.org/10.1093/ije/dyv072[68]

Cohort Description

Pregnant women resident in Avon, UK with expected dates of delivery between 1st April 1991 and 31st December 1992 were invited to take part in the study. 20,248 pregnancies have been identified as being eligible and the initial number of pregnancies enrolled was 14,541. Of the initial pregnancies, there was a total of 14,676 foetuses, resulting in 14,062 live births and 13,988 children who were alive at 1 year of age[67]. When the oldest children were approximately 7 years of age, an attempt was made to bolster the initial sample with eligible cases who had failed to join the study originally. As a result, when considering variables collected from the age of seven onwards (and potentially abstracted from obstetric notes) there are data available for more than the 14,541 pregnancies mentioned above: The number of new pregnancies not in the initial sample (known as Phase I enrolment) that are currently represented in the released data and reflecting enrolment status at the age of 24 is 906, resulting in an additional 913 children being enrolled (456, 262 and 195 recruited during Phases II, III and IV respectively. The phases of enrolment are described in more detail in the cohort profile paper and its update[69]. The total sample size for analyses using any data collected after the age of seven is therefore 15,447 pregnancies, resulting in 15,658 foetuses. Of these 14,901 children were alive at 1 year of age. As part of Accessible Resource for Integrated Epigenomic Studies (ARIES), a sub-sample ALSPAC children, mothers and partners had DNAm assayed using the Illumina Infinium HumanMethylation450 or MethylationEPIC Beadchip array from blood samples collected from ALSPAC children and their parents at multiple time points from birth to middle age[68]. Study data were collected and managed using REDCap electronic data capture tools hosted at the University of Bristol. REDCap (Research Electronic Data Capture) is a secure, web-based software platform designed to support data capture for research studies[70]. Please note that the study website contains details of all the data that is available through a fully searchable data dictionary and variable search tool (http://www.bristol.ac.uk/alspac/researchers/our-data/).

Ethical Approval: Ethical approval for the study was obtained from the ALSPAC Ethics and Law Committee and the Local Research Ethics Committees under proposal B3818. Consent for biological samples has been collected in accordance with the Human Tissue Act (2004).

Antidepressant exposure phenotype

Medications, including antidepressants, were recorded for children of ALSPAC at age 24 at the clinic appointment for the 'Focus @ 24' timepoint ('F@24'), when blood sampling also took place. Additionally, the 'Life @ 28' questionnaire, collected between December 2020 and April 2021, had a Mental Health Treatments section which included in-depth questions regarding antidepressant use, including the duration of adherence[71].

Those who reported antidepressant use at the F@24 clinic (FieldID: FCKO1103) and/or those who reported antidepressant use for over 4 years at the F@28 questionnaire, that also overlapped with the F@24 clinic

(FieldIDs: YPH7000, YPH7017 and YPH7047) were defined as antidepressant exposed. Those who reported no antidepressant use at the F@24 clinic assessment and/or no antidepressant use at the F@28 questionnaire or were not taking antidepressants at the time of the blood sample were classed as antidepressant-unexposed. In total, there were 43 exposed and 758 unexposed individuals with quality DNAm data for analysis.

DNAm preprocessing

Illumina Infinium HumanMethylation450 and MethylationEPIC Beadchip arrays were used to assess genome-wide DNAm patterns in peripheral blood. Samples across different time-points were distributed in a semi-random manner across slides to mitigate batch effects. Data quality assessment, pre-processing and normalization was performed using the R package *meffil* as previously described[15]. Briefly, probes undetected (detection p-value > 0.01) or with low bead count (<3) in >10% of samples were removed. Samples were removed if there was a mismatch between their predicted sex and recorded sex or if >10% of probes were undetected. Raw probe intensities were adjusted for potential dye bias, background corrected using the *'noob'* method[16] and normalized using functional normalization[57]. Cell proportions (CD4T, CD8T, NK cells, B cells, Granulocytes and Monocytes) were estimated using the Houseman mode in the *minfi* R package[13]. Lymphocyte cell proportions were then calculated as the aggregate of the estimated CD4T, CD8T, NK and Bcell cell proportions. M-value transformation was conducted using the *'beta2m()'* function in the *lumi* R package[19]. The M-values were then standardised using Z-score normalisation using the *'scale()'* function in R. In total, 857,809 CpG probes remained for 801 individuals.

Methylation Profile Score

After adhering to the quality control protocols, all 212 CpGs were available for the calculation of the MPS, which was derived as a weighted sum of the CpGs. The rate of missingness per CpG ranged from 0-6%. In the case that an individual had a missing value for a CpG(s), the probe(s) were excluded from the MPS (i.e., given a weight of 0).

Antidepressant exposure ~ MPS association model

The association between the antidepressant exposure and MPS was tested using a generalised linear model, using the *'glm()'* function from the *stats* R package[49], with antidepressant exposure as the outcome. The model included the following predictors: antidepressant exposure MPS, age at sampling, sex, monocyte cell proportions, lymphocyte cell proportions and AHRR M values ('cg05575921') to proxy for smoking status.

```
glm(formula = as.factor(antidep) ~ scale(AD_MRS) + scale(age) +
    scale(Mono) + scale(lymphocytes) + scale(cg05575921) + as.factor(sex_coded),
    family = "binomial", data = MRS_covs_pheno)
```

Funding and Acknowledgements

The UK Medical Research Council and Wellcome (Grant Ref: 217065/Z/19/Z) and the University of Bristol provide core support for ALSPAC. ASFK is funded by a Wellcome Early Career Award (Grant ref: 227063/Z/23/Z). A comprehensive list of grants funding is available on the ALSPAC website

(http://www.bristol.ac.uk/alspac/external/documents/grant-acknowledgements.pdf). This publication is the work of the authors and ED, PY & ASFK will serve as guarantors for the ALSPAC contents of this paper. We thank all the families who took part in this study, the midwives for their help in recruiting them, and the whole ALSPAC team, which includes interviewers, computer and laboratory technicians, clerical workers, research scientists, volunteers, managers, receptionists and nurses.

Environmental risk (E-Risk) Longitudinal Twin Study

Protocol paper: https://doi.org/10.1111/1469-7610.00082[72]

Cohort Description

The Environmental Risk (E-Risk) Longitudinal Twin Study is a representative birth cohort study of 2,232 twins born in England and Wales between 1994-1995. Baseline data collection occurred between 1999-2000 when 1,116 families (93% of those eligible) with same-sex 5-year-old twins participated in home-visit assessments. Full details about the sample are reported elsewhere[72]. This sample comprised 56% monozygotic and 44% dizygotic twin pairs; sex was evenly distributed within zygosity (49% male). This is a nationally representative sample - E-Risk families' addresses are currently a near-perfect match to the deciles of the UK's 2015 Lower-layer Super Output Area Index of Multiple Deprivation (IMD). Follow-up home-visits were conducted when children were aged 7, 10, 12, and 18 (participation rates were 98%, 96%, 96%, and 93%, respectively). At age 18, 2066 participants were assessed. Average age at time of assessment was 18.4 years (SD=0.36); all interviews were conducted after the 18th birthday. There were no differences between those who did and did not take part at age 18 in terms of socio-economic status (SES) assessed when the cohort was initially defined ($\chi2=0.86$, $p=0.65$), age-5 IQ scores ($t=0.98$, $p=0.33$), and age-5 behavioural ($t=0.40$, $p=0.69$) or emotional ($t=0.41$, $p=0.68$) problems.

Ethical Approval The Joint South London and Maudsley and Institute of Psychiatry Research Ethics Committee approved each study phase (NRES 1997/122). Parents gave informed consent, and twins gave assent between 5 and 12 years and then informed consent at age 18.

Antidepressant exposure phenotype

Antidepressant exposure was assessed at the age- 18 home visit, where participants were asked: "Have you taken any medicines in the last two weeks?" and they were then shown a card listing common medications including antidepressants. If they reported antidepressant use then they were asked to provide the name of the specific drug(s) they had taken, what condition it was being used to treat, and the duration of use. Medicines were classed by a consultant psychiatrist as antidepressants following BNF criteria. Those who reported using one or more antidepressant medications in the previous 2 weeks were defined as antidepressant-exposed, and those who did not report any use of antidepressant medication in this period were defined as antidepressant-unexposed. In total, there were 36 exposed and 1,622 unexposed with high quality DNAm data.

DNAm preprocessing

DNAm was profiled using 1,700 whole blood samples collected at the age-18 home visit, using the Illumina Infinium HumanMethylation450 BeadChip. Of the available samples, 31 samples were excluded due to a low DNA concentration. Approximately 500ug of DNA from each sample was treated with sodium bisulfite using EZ-96 DNA Methylation Kit (Zymo Research, CA, USA). DNAm was then quantified using the Illumina Infinium HumanMethylation450 BeadChip (Illumina Inc, CA, USA) run on an Illumina iScan System (Illumina, CA, USA)

using the manufacturers' standard protocol. Twin pairs were randomly assigned to bisulfite-coversion plates and Illumina 450K arrays, with siblings positioned adjacently to minimise batch effects.

The following quality control (QC) pipeline was applied to all 1,669 unique individuals and was performed in the R statistical programming environment. Data was imported using the *'methylumIDAT()'* function from the *methylumiI* package[73]. Samples were excluded if median methylation ('M') and unmethylation ('U') signal intensities were < 2,500 (n = 10). The fully methylated control samples were first identified using their intensity profiles (signal characteristic of being fully methylated) and used to confirm the absence of any plate rotations or plate mislabelling had occurred before being removed from the dataset. The efficacy of the sodium bisulfite conversion reaction was assessed using ten control probes included on the 450K array, and probes with a "conversion score" < 80 were removed. Multidimensional scaling was performed for DNAm probes on each of the sex chromosomes and compared to reported sex, finding a discrepancy for 2 samples from 2 different monozygotic twin pairs. Further investigation into these twin pairs concluded that discordant samples had been mistakenly switched in the lab, and therefore paired with the twin from other twin pair. Correction of ID identifiers led to a 100% match. The genotype concordance between SNP probes on the 450K array and data generated using Illumina OmniExpress24v1.2 genotyping BeadChips was assessed to confirm genetic identity of DNA samples. Data were available for 35 of the 65 SNP probes from both platforms for 1,638 (98.7%) of the samples. A total of 1,658 samples passed the stringent QC pipeline. The *'pfilter()'* function from the *wateRmelon* package[12] in R was used to process the data and exclude samples which had > 1% of probes having a detection p value > 0.05 (n = 0). Probes which had a beadcount < 3 in 5% of the samples (n = 567) and those with a detection p value > 0.05 in >1% of the samples were removed from the dataset. The data was then normalised using the dasen method from the *wateRmelon* package[12]. Prior to any analyses, probes with common (> 5% MAF) SNPs within 10bp of the single base extension and probes with sequences previously identified as potentially hybridising to multiple genomic loci were excluded (n = 52,760)[40]. After QC and annotation of CpGs to the 450k array 430,802 sites remained for analysis. Methylation beta-values were transformed to M-values using logit transformation. These Illumina DNA methylation data are accessible from the Gene Expression Omnibus (accession code: GSE105018).

Methylation Profile Score

After adhering to the quality control protocols, 207 CpGs were available for the calculation of the MPS, which was derived as a weighted sum of the CpGs. There was no missingness in any of the CpGs included in the score.

Antidepressant exposure ~ MPS association model

The association between antidepressant exposure and the MPS was assessed using a generalised linear mixed model, using the 'glmer()' function from the 'lme4' R package1, with antidepressant exposure as the outcome. The model included the following predictors: antidepressant exposure MPS, sex, predicted monocyte cell proportions, predicted lymphocyte cell proportions, M values at the AHRR probe (cg05575921), and family ID as a random effect. The model was specified with a logit link function and 20 quadrature points in the adaptive Gaussian quadrature approximation for integrating over the random effect. The optimisation algorithm used

was the Bound Optimisation by Quadratic Approximation (BOBYQA) with a maximum of 100,000 function evaluations.

```
assoc_mod<- glmer(as.factor(antidep) ~ scale(AD_MRS) +
                           as.factor(sex_coded) + scale(Mono) + scale(lymphocytes)+ scale
(as.numeric(Chip))+ scale(cg05575921)+ (1|FID), data=MRS_covs_pheno, family=binomial(link
= "logit"), nAGQ = 20,
                        control = glmerControl(optimizer = "bobyqa", optCtrl = list(maxfu
n = 100000)))
```

Funding and Acknowledgements

The E-Risk Study is funded by grants from the UK Medical Research Council [G1002190; MR/X010791/1]. Additional support was provided by the US National Institute of Child Health and Human Development [HD077482] and the Jacobs Foundation. Chloe C. Y. Wong was supported by the National Institute for Health and Care Research (NIHR) Maudsley Biomedical Research Centre at South London and Maudsley NHS Foundation Trust and King's College London [NIHR203318]. Helen L. Fisher was supported by the Economic and Social Research Council (ESRC) Centre for Society and Mental Health at King's College London [ES/S012567/1]. The views expressed are those of the authors and not necessarily those of the NIHR, the Department of Health and Social Care, the ESRC, or King's College London.

We are grateful to the E-Risk study mothers and fathers, the twins, and the twins' teachers for their participation. Our thanks to Professors Terrie Moffitt and Avshalom Caspi, the founders of the E-Risk study, and to the E-Risk team for their dedication, hard work, and insights.

Prospective Generation Scotland Follow Up

Stratifying Resilience and Depression Longitudinally

Protocol paper: https://doi.org/10.1093/ije/dyx115[75]

Cohort Description

The Stratifying Resilience and Depression Longitudinally (STRADL) cohort[75], is a follow up study of a subset of GS participants (n = 1, 168) on average five years after baseline assessment. STRADL was primarily aimed for the assessment of mental health, specifically MDD. Data collection included socio-economic and demographic profiling, laboratory samples, extensive psychological assessment and magnetic resonance imaging (MRI)[76]. Further details of the STRADL cohort and GS protocol are published elsewhere[9,76].

Ethical Approval: All components of STRADL received formal, national ethical approval from the NHS Tayside committee on research ethics (reference 14/SS/0039).

Antidepressant exposure phenotype

Medication use in STRADL was assessed using a free-response questionnaire asking participants to list prescribed or bought medications, classifying antidepressants using the British National Formulary (BNF) framework. We took forward those who did not self-report antidepressant use (controls) at Generation Scotland baseline (n = 15, 028) and who were present in the STRADL subsample (n = 901). Those who did or did not list an antidepressant medication at the STRADL reassessment time point were defined as antidepressant-exposed and unexposed respectively. In total, there were 46 exposed and 617 unexposed individuals with high-quality DNAm data.

DNAm preprocessing

DNAm was profiled from whole-blood samples taken at the STRADL timepoint using the Illumina HumanMethylation EPIC array. The processing was completed in two sets and used the same methodology as those collected in the wider Generation Scotland cohort at baseline[9]. The R package *Meffil*[15] was used to remove samples which: had a mismatch between genotyped and DNAm-predicted sex, had > 0.5% of CpGs with a detection P > 0.01, were outliers for bisulphite conversion control probes, had median signal intensity >3 standard deviations lower than expected and those with evidence of dye bias. The R package *shinyMethyl*[11] was used to exclude outliers based on visual inspection of the log median intensity of methylated vs unmethylated signals per array. Following this, *Meffil* was again implemented to identify and exclude probes with a beadcount < 3 in > 5% of the samples and/or probes in which > 1% of the samples had a detection of P > 0.01. Multidimensional scaling (MDS) was performed to inspect for further outlier samples and identified 40 males outlying according to the X chromosome DNAm levels and were subsequently removed from the analysis. Data were renormalised and inspection of MDS confirmed no further outliers present across both sets. Cell proportions (CD4T, CD8T, NK cells, B cells, Granulocytes and Monocytes) were estimated using the Houseman mode in the *minfi* R package[13]. Lymphocyte cell proportions were then calculated as the aggregate

of the estimated CD4T, CD8T, NK and Bcell cell proportions. Data were then normalised using the dasen method in the *wateRmelon* R package[12] and converted to M values using the *'beta2m()'* function in the *lumi* R package[19]. After quality control, there were 793, 706 (n = 503) and 775, 284 (n = 372) CpGs available from set 1 and 2 respectively. The sets were filtered to CpGs common to both and joined together, resulting in 774, 073 CpGs for 875 individuals.

Methylation Profile Score

After adhering to the quality control protocols, all 212 CpGs were available for the calculation of the MPS, which was derived as a weighted sum of the CpGs. There was no missingness in any of the CpGs included in the score.

Antidepressant exposure ~ MPS association mode

The association between the antidepressant exposure and MPS was tested using a generalised linear mixed effect linear model, using the *'glmer()'* function from the *lme4* R package[46]. Directly measured fixed effect covariates included age, sex, AHRR probe M-values ('cg05575921') to proxy for smoking status, and estimated white blood cell proportions (monocytes and lymphocytes). The array processing batch and family ID was included as a random effect to account for additional technical factors and relatedness within the cohort.

```
Generalized linear mixed model fit by maximum likelihood (Laplace
  Approximation) [glmerMod]
 Family: binomial  ( logit )
Formula: as.factor(antidep) ~ scale(AD_MRS) + scale(age) + scale(Mono) +
    scale(lymphocytes) + scale(cg05575921) + as.factor(sex_coded) +
    (1 | batch) + (1 | famid)
```

Funding and Acknowledgement

STRADL is supported by the Wellcome Trust through a Strategic Award (reference 104036/Z/14/Z). The Chief Scientist Office of the Scottish Government Health Department (CZD/16/6) and the Scottish Funding Council (HR03006) provided core support for Generation Scotland. A.M.M. is supported by the Dr Mortimer and Theresa Sackler Foundation. Funding from the Medical Research Council and Biotechnology and Biological Sciences Research Council is gratefully acknowledged (MR/K026992/1). We would like to express gratitude to all individuals who have taken part in both GS:SFHS and STRADL, and the entire project team including academic researchers, administrative staff, research managers and statisticians.

References

1. Kassambara A. *Ggpubr: 'Ggplot2' Based Publication Ready Plots*.; 2023. https://rpkgs.datanovia.com/ggpubr/

2. Campagna MP, Xavier A, Lechner-Scott J, et al. Epigenome-wide association studies: Current knowledge, strategies and recommendations. *Clinical epigenetics*. 2021;13:1-24.

3. Suderman M, Staley JR, French R, Arathimos R, Simpkin A, Tilling K. Dmrff: Identifying differentially methylated regions efficiently with power and control. *BioRxiv*. Published online 2018:508556.

4. Martin TC, Yet I, Tsai PC, Bell JT. coMET: Visualisation of regional epigenome-wide association scan results and DNA co-methylation patterns. *BMC bioinformatics*. 2015;16(1):1-5.

5. Hansen K, Aryee M. IlluminaHumanMethylationEPICmanifest: Manifest for illumina's EPIC methylation arrays. *R package version*. 2016;3(0).

6. Watanabe K, Taskesen E, Van Bochoven A, Posthuma D. Functional mapping and annotation of genetic associations with FUMA. *Nature communications*. 2017;8(1):1826.

7. Koopmans F, Nierop P van, Andres-Alonso M, et al. SynGO: An evidence-based, expert-curated knowledge base for the synapse. *Neuron*. 2019;103(2):217-234.

8. Phipson B, Maksimovic J, Oshlack A. missMethyl: An r package for analyzing data from illumina's HumanMethylation450 platform. *Bioinformatics*. 2015;32(2):286-288.

9. Smith BH, Campbell A, Linksted P, et al. Cohort profile: Generation scotland: Scottish family health study (GS: SFHS). The study, its participants and their potential for genetic research on health and illness. *International journal of epidemiology*. 2013;42(3):689-700.

10. Smith BH, Campbell A, Linksted P, et al. Cohort profile: Generation scotland: Scottish family health study (GS:SFHS). The study, its participants and their potential for genetic research on health and illness. *International Journal of Epidemiology*. 2012;42(3):689-700. doi:10.1093/ije/dys084

11. Fortin JP, Fertig E, Hansen K. shinyMethyl: Interactive quality control of illumina 450k DNA methylation arrays in r. *F1000Research*. 2014;3.

12. Pidsley R, Y Wong CC, Volta M, Lunnon K, Mill J, Schalkwyk LC. A data-driven approach to preprocessing illumina 450K methylation array data. *BMC genomics*. 2013;14(1):1-10.

13. Aryee MJ, Jaffe AE, Corrada-Bravo H, et al. Minfi: A flexible and comprehensive bioconductor package for the analysis of infinium DNA methylation microarrays. *Bioinformatics*. 2014;30(10):1363-1369.

14. Reinius LE, Acevedo N, Joerink M, et al. Differential DNA methylation in purified human blood cells: Implications for cell lineage and studies on disease susceptibility. *PloS one*. 2012;7(7):e41361.

15. Min JL, Hemani G, Davey Smith G, Relton C, Suderman M. Meffil: Efficient normalization and analysis of very large DNA methylation datasets. *Bioinformatics*. 2018;34(23):3983-3989.

16. Triche Jr TJ, Weisenberger DJ, Van Den Berg D, Laird PW, Siegmund KD. Low-level processing of illumina infinium DNA methylation beadarrays. *Nucleic acids research*. 2013;41(7):e90-e90.

17. Zhou W, Laird PW, Shen H. Comprehensive characterization, annotation and innovative use of infinium DNA methylation BeadChip probes. *Nucleic acids research*. 2017;45(4):e22-e22.

18. McCartney DL, Walker RM, Morris SW, McIntosh AM, Porteous DJ, Evans KL. Identification of polymorphic and off-target probe binding sites on the illumina infinium MethylationEPIC BeadChip. *Genomics data*. 2016;9:22-24.

19. Du P, Kibbe WA, Lin SM. Lumi: A pipeline for processing illumina microarray. *Bioinformatics*. 2008;24(13):1547-1548.

20. Kerr SM, Campbell A, Marten J, et al. Electronic health record and genome-wide genetic data in generation scotland participants. *Wellcome Open Research*. 2017;2.

21. Alvarez-Madrazo S, McTaggart S, Nangle C, Nicholson E, Bennie M. Data resource profile: The scottish national prescribing information system (PIS). *International journal of epidemiology*. 2016;45(3):714-715f.

22. First M, Spitzer R, Gibbon M, et al. J., kane, j., pope, HG, rounsaville, b.(1995). The structured clinical interview for DSM-III-r personality disorders (SCID-II). Part II: Multi-site test-retest reliability study. *Journal of Personality Disorders*. 9:92-104.

23. Fernandez-Pujals AM, Adams MJ, Thomson P, et al. Epidemiology and heritability of major depressive disorder, stratified by age of onset, sex, and illness course in generation scotland: Scottish family health study (GS: SFHS). *PloS one*. 2015;10(11):e0142197.

24. Penninx BW, Beekman AT, Smit JH, et al. The netherlands study of depression and anxiety (NESDA): Rationale, objectives and methods. *International journal of methods in psychiatric research*. 2008;17(3):121-140.

25. Aberg KA, Chan RF, Oord EJ van den. MBD-seq-realities of a misunderstood method for high-quality methylome-wide association studies. *Epigenetics*. 2020;15(4):431-438.

26. Aberg KA, Chan RF, Shabalin AA, et al. A MBD-seq protocol for large-scale methylome-wide studies with (very) low amounts of DNA. *Epigenetics*. 2017;12(9):743-750.

27. Chan RF, Shabalin AA, Xie LY, et al. Enrichment methods provide a feasible approach to comprehensive and adequately powered investigations of the brain methylome. *Nucleic acids research*. 2017;45(11):e97-e97.

28. Langmead B, Salzberg SL. Fast gapped-read alignment with bowtie 2. *Nature methods*. 2012;9(4):357-359.

29. Shabalin AA, Hattab MW, Clark SL, et al. RaMWAS: Fast methylome-wide association study pipeline for enrichment platforms. *Bioinformatics*. 2018;34(13):2283-2285.

30. Filzmoser P, Gschwandtner M. *Mvoutlier: Multivariate Outlier Detection Based on Robust Methods*.; 2021. https://CRAN.R-project.org/package=mvoutlier

31. Aberg KA, McClay JL, Nerella S, et al. MBD-seq as a cost-effective approach for methylome-wide association studies: Demonstration in 1500 case–control samples. *Epigenomics*. 2012;4(6):605-621.

32. Oord EJ van den, Bukszar J, Rudolf G, et al. Estimation of CpG coverage in whole methylome next-generation sequencing studies. *BMC bioinformatics*. 2013;14:1-10.

33. Cabrera CP, Navarro P, Huffman JE, et al. Uncovering networks from genome-wide association studies via circular genomic permutation. *G3: Genes| Genomes| Genetics*. 2012;2(9):1067-1075.

34. Chan RF, Turecki G, Shabalin AA, et al. Cell type–specific methylome-wide association studies implicate neurotrophin and innate immune signaling in major depressive disorder. *Biological psychiatry*. 2020;87(5):431-442.

35. Kaprio J, Bollepalli S, Buchwald J, et al. The older finnish twin cohort—45 years of follow-up. *Twin Research and Human Genetics*. 2019;22(4):240-254.

36. Rose RJ, Salvatore JE, Aaltonen S, et al. FinnTwin12 cohort: An updated review. *Twin Research and Human Genetics*. 2019;22(5):302-311.

37. Kaidesoja M, Aaltonen S, Bogl LH, et al. FinnTwin16: A longitudinal study from age 16 of a population-based finnish twin cohort. *Twin Research and Human Genetics*. 2019;22(6):530-539.

38. Koskinen SM, Ahveninen J, Kujala T, et al. A longitudinal twin study of effects of adolescent alcohol abuse on the neurophysiology of attention and orienting. *Alcoholism: Clinical and Experimental Research*. 2011;35(7):1339-1350.

39. Huang Y, Ollikainen M, Sipilä P, et al. Genetic and environmental effects on gene expression signatures of blood pressure: A transcriptome-wide twin study. *Hypertension*. 2018;71(3):457-464.

40. Chen Y, Lemire M, Choufani S, et al. Discovery of cross-reactive probes and polymorphic CpGs in the illumina infinium HumanMethylation450 microarray. *Epigenetics*. 2013;8(2):203-209.

41. Zheng SC, Breeze CE, Beck S, Teschendorff AE. Identification of differentially methylated cell types in epigenome-wide association studies. *Nature methods*. 2018;15(12):1059-1066.

42. Teschendorff AE, Breeze CE, Zheng SC, Beck S. A comparison of reference-based algorithms for correcting cell-type heterogeneity in epigenome-wide association studies. *BMC bioinformatics*. 2017;18:1-14.

43. Vanegas LH, Rondón LM, Paula GA. *Glmtoolbox: Set of Tools to Data Analysis Using Generalized Linear Models*.; 2024. https://CRAN.R-project.org/package=glmtoolbox

44. Völzke H, Schössow J, Schmidt CO, et al. Cohort profile update: The study of health in pomerania (SHIP). *International Journal of Epidemiology*. 2022;51(6):e372-e383.

45. Jürgens C, Völzke H, Tost F. Study of health in pomerania (SHIP-trend): Important aspects for healthcare research in ophthalmology. *Der Ophthalmologe*. 2014;111:443-447.

46. Bates D, Mächler M, Bolker B, Walker S. Fitting linear mixed-effects models using lme4. *arXiv preprint arXiv:14065823*. Published online 2014.

47. Kircher T, Wöhr M, Nenadic I, et al. Neurobiology of the major psychoses: A translational perspective on brain structure and function—the FOR2107 consortium. *European archives of psychiatry and clinical neuroscience*. 2019;269:949-962.

48. Heiss JA, Just AC. Identifying mislabeled and contaminated DNA methylation microarray data: An extended quality control toolset with examples from GEO. *Clinical epigenetics*. 2018;10(1):1-9.

49. R Core Team. *R: A Language and Environment for Statistical Computing*. R Foundation for Statistical Computing; 2023. https://www.R-project.org/

50. Ligthart L, Beijsterveldt CE van, Kevenaar ST, et al. The netherlands twin register: Longitudinal research based on twin and twin-family designs. *Twin Research and Human Genetics*. 2019;22(6):623-636.

51. Willemsen G, De Geus EJ, Bartels M, et al. The netherlands twin register biobank: A resource for genetic epidemiological studies. *Twin Research and Human Genetics*. 2010;13(3):231-245.

52. Boomsma DI, Willemsen G, Sullivan PF, et al. Genome-wide association of major depression: Description of samples for the GAIN major depressive disorder study: NTR and NESDA biobank projects. *European Journal of Human Genetics*. 2008;16(3):335-342.

53. Van Dongen J, Nivard MG, Willemsen G, et al. Genetic and environmental influences interact with age and sex in shaping the human methylome. *Nature communications*. 2016;7(1):11115.

54. Bonder MJ, Luijk R, Zhernakova DV, et al. Disease variants alter transcription factor levels and methylation of their binding sites. *Nature genetics*. 2017;49(1):131-138.

55. Van Iterson M, Tobi EW, Slieker RC, et al. MethylAid: Visual and interactive quality control of large illumina 450k datasets. *Bioinformatics*. 2014;30(23):3435-3437.

56. Whole-genome sequence variation, population structure and demographic history of the dutch population. *Nature genetics*. 2014;46(8):818-825.

57. Fortin JP, Labbe A, Lemire M, et al. Functional normalization of 450k methylation array data improves replication in large cancer studies. *Genome biology*. 2014;15(11):1-17.

58. Hennings JM, Owashi T, Binder EB, et al. Clinical characteristics and treatment outcome in a representative sample of depressed inpatients–findings from the munich antidepressant response signature (MARS) project. *Journal of psychiatric research*. 2009;43(3):215-229.

59. Lucae S, Salyakina D, Barden N, et al. P2RX7, a gene coding for a purinergic ligand-gated ion channel, is associated with major depressive disorder. *Human molecular genetics*. 2006;15(16):2438-2445.

60. Auton Adam adam. auton@ gmail. com 1 b Abecasis Gonçalo R. goncalo@ umich. edu 2 c 1000GPCC authors, Baylor College of Medicine Gibbs Richard A.(Principal Investigator) 14 Boerwinkle Eric 14 Doddapaneni Harsha 14 Han Yi 14 Korchina Viktoriya 14 Kovar Christie 14 Lee Sandra 14 Muzny Donna 14 Reid Jeffrey G. 14 Zhu Yiming 14 P group, MIT BI of, et al. A global reference for human genetic variation. *Nature*. 2015;526(7571):68-74.

61. Johnson WE, Li C, Rabinovic A. Adjusting batch effects in microarray expression data using empirical bayes methods. *Biostatistics*. 2007;8(1):118-127.

62. Zhang Q, Marioni RE, Robinson MR, et al. Genotype effects contribute to variation in longitudinal methylome patterns in older people. *Genome medicine*. 2018;10(1):1-11.

63. Deary IJ, Gow AJ, Taylor MD, et al. The lothian birth cohort 1936: A study to examine influences on cognitive ageing from age 11 to age 70 and beyond. *BMC geriatrics*. 2007;7:1-12.

64. Shah S, McRae AF, Marioni RE, et al. Genetic and environmental exposures constrain epigenetic drift over the human life course. *Genome research*. 2014;24(11):1725-1733.

65. Teschendorff AE, Marabita F, Lechner M, et al. A beta-mixture quantile normalization method for correcting probe design bias in illumina infinium 450 k DNA methylation data. *Bioinformatics*. 2013;29(2):189-196.

66. Boyd A, Golding J, Macleod J, et al. Cohort profile: The "children of the 90s"—the index offspring of the avon longitudinal study of parents and children. *International journal of epidemiology*. 2013;42(1):111-127.

67. Fraser A, Macdonald-Wallis C, Tilling K, et al. Cohort profile: The avon longitudinal study of parents and children: ALSPAC mothers cohort. *International journal of epidemiology*. 2013;42(1):97-110.

68. Relton CL, Gaunt T, McArdle W, et al. Data resource profile: Accessible resource for integrated epigenomic studies (ARIES). *International journal of epidemiology*. 2015;44(4):1181-1190.

69. Northstone K, Lewcock M, Groom A, et al. The avon longitudinal study of parents and children (ALSPAC): An update on the enrolled sample of index children in 2019. *Wellcome open research*. 2019;4.

70. Harris PA, Taylor R, Thielke R, Payne J, Gonzalez N, Conde JG. Research electronic data capture (REDCap)—a metadata-driven methodology and workflow process for providing translational research informatics support. *Journal of biomedical informatics*. 2009;42(2):377-381.

71. Fraser H, White G, Matthews S, et al. Medication use for depression and anxiety: Data collected from 28/29 year-old offspring in the avon longitudinal study of parents and children. *Wellcome Open Research*. 2023;8:237.

72. Moffitt TE, Team ERS. Teen-aged mothers in contemporary britain. *Journal of Child Psychology and Psychiatry*. 2002;43(6):727-742.

73. Davis S, Du P, Bilke S, Triche T, Bootwalla M. Methylumi: Handle illumina methylation data. *R package version*. 2015;2(0).

74. Price EM, Cotton AM, Lam LL, et al. Additional annotation enhances potential for biologically-relevant analysis of the illumina infinium HumanMethylation450 BeadChip array. *Epigenetics & chromatin*. 2013;6:1-15.

75. Navrady L, Wolters M, MacIntyre D, et al. Cohort profile: Stratifying resilience and depression longitudinally (STRADL): A questionnaire follow-up of generation scotland: Scottish family health study (GS: SFHS). *International journal of epidemiology*. 2018;47(1):13-14g.

76. Habota T, Sandu AL, Waiter GD, et al. Cohort profile for the STratifying resilience and depression longitudinally (STRADL) study: A depression-focused investigation of generation scotland, using detailed clinical, cognitive, and neuroimaging assessments. *Wellcome open research*. 2019;4.
