# Supplementary material for: Insights from a methylome-wide association study of antidepressant exposure
Source: Nat Commun. 2025 Feb 24;16:1908. doi: 10.1038/s41467-024-55356-x (PMC11850842; doi:10.1038/s41467-024-55356-x)
Supplement: Supplementary file 3 — Description of Additional Supplementary Files [file 41467_2024_55356_MOESM3_ESM.pdf]

Description of Additional Supplementary Files

File Name: Supplementary Data 1

Description: CpGs with a nominal significant difference in effect estimates between male only MWAS and female only MWAS of self-report antidepressant exposure caluclated using a Mixed-linear-model Omics-based Analysis (MOA) model (Nfemales = 9,710, Nmales = 6,821). The b_sexdiff refers to b_male-b_female, it's corresponding Z-score (calculated as the b_sexdiff divided by the square root of the sum of the standard errors in the male and female MWAS estimates) and p-value for the difference under the standard normal distribution. P-values were adjusted based on the false discovery rate (FDR).

File Name: Supplementary Data 2

Description: Differentially methylated regions (DMRs) identified using dmrff() R package of our self-report MWAS results. Dmrff performs an inverse-variance-weighted meta-analysis of MWAS beta and standard-error estimates per region, adjusting for estimate uncertainty and the correlational structure between probes. Candidate DMRs are identified as sets (>2) of CpGs <= 500bp apart with nominal significance (P < 0.05) and consistent effect direction. DMRs achieving Bonferroni-corrected p-value < 0.05 (p.adjust) were considered statistically significant. Regions (>=2 CpGs) are reported in order of their p-value.

File Name: Supplementary Data 3

Description: Differentially methylated regions (DMRs) identified using dmrff() R package of our prescription-derived MWAS results. Dmrff performs an inverse-variance-weighted meta-analysis of MWAS beta and standard-error estimates per region, adjusting for estimate uncertainty and the correlational structure between probes. Candidate DMRs are identified as sets (>2) of CpGs <= 500bp apart with nominal significance (P < 0.05) and consistent effect direction. DMRs achieving Bonferroni-corrected p-value < 0.05 (p.adjust) were considered statistically significant. Regions (>=2 CpGs) are reported in order of their p-value.

File Name: Supplementary Data 4

Description: The top 100 CpGs (most significant/lowest P value) found in the self-report MWAS using a Mixed-linear-model Omics-based Analysis (MOA) model (N = 16,531) and

their annotated genes. Significance was assessed using the p-value threshold 9.42x10-8, as recommended for case-control MWAS analyses.

File Name: Supplementary Data 5

Description: The top 100 CpGs (most significant/lowest P value) found in the prescription-derived MWAS using a Mixed-linear-model Omics-based Analysis (MOA) model (N = 7,951) and their annotated genes. Significance was assessed using the p-value threshold 9.42x10-8, as recommended for case-control MWAS analyses.

File Name: Supplementary Data 6

Description: The genes annotated to the top 100 CpGs in the self-report MWAS and the number of CpGs annotated to each gene. Ordered by the number of annotated CpGs.

File Name: Supplementary Data 7

Description: The genes annotated to the top 100 CpGs in the prescription-derived MWAS and the number of CpGs annotated to each gene. Ordered by the number of annotated CpGs.

File Name: Supplementary Data 8

Description: FUMA GENE2FUNC Tissue Specificity results for 54 tissues in GTEx (v8) for the self-report gene-set, which uses hypergeometric tests to assess whether there if genes are overrepresented in a differentially expressed gene (DEG) set for each tissue. The background gene set used was a list of genes which could be annotated by the EPIC array. The results are ordered by FDR-adjusted P value (adjP), calculated using the Benjamini-Hochberg procedure.

File Name: Supplementary Data 9

Description: FUMA GENE2FUNC Tissue Specificity results for 54 tissues in GTEx (v8) for the prescription-derived gene-set which uses hypergeometric tests to assess whether there if genes are overrepresented in a differentially expressed gene (DEG) set for each tissue. The background gene set used was a list of genes which could be annotated by the EPIC array. The results are ordered by FDR-adjusted P value (adjP), calculated using the Benjamini-Hochberg procedure.

File Name: Supplementary Data 10

Description: Self-report SynGO enrichment. Enrichment results of SynGO ontologies for those with at least one gene present in the self-report gene list, from the SynGo Web Portal which uses hypergeometric tests to assess enrichment.

File Name: Supplementary Data 11

Description: Prescription-derived SynGO enrichment.\n\nEnrichment results of SynGO ontologies for those with at least one gene present in the prescription-derived gene list, from the SynGo Web Portal which uses hypergeometric tests to assess enrichment.

File Name: Supplementary Data 12

Description: Enrichment of the self-report gene-set in MSigDB BP Gene set ontologies, using gsameth() from missMethyl which uses a hypergeometric test to assess enrichment. Results are ordered by nominal P value.

File Name: Supplementary Data 13

Description: Enrichment of the prescription-derived gene-set in MSigDB BP Gene set ontologies, using gsameth() from missMethyl which uses a hypergeometric test to assess enrichment.. Results are ordered by nominal P value.

File Name: Supplementary Data 14

Description: The overlapping methylation sites between the top 1% of Generation Scotland probes (Illumina EPIC array) and top 1% NESDA (MBD-Sequencing) primers in the self-reported antidepressant MWAS, which were significantly enriched (OR = 1.39, P = 0.042). The tests for enrichment in the overlap were conducted using Cramer's V. A total of 100,000 permutations were performed using the shiftR R package, and p values were calculated as the proportion of permutations that yielded a value equal or greated than Cramers V observed in the observed data. Columns ending in NESDA relate to the MWAS statistics for the primer in MBD-Sequencing and columns ending in GS relate to MWAS statistics for the probe in the Illumina array. Note the same illumina probe may occur multiple times as we allow for a 150bp flank on either side of the probe when mapping to improve power by ensuring more MBD-seq sites can be linked to Illumina probes. CpGs located within such short distances are typically highly correlated and can therefore serve as a "proxy". The circular permutations in the enrichment analysis account for the dependancy that this produces.

File Name: Supplementary Data 15

Description: All CpGs with non-zero weights in the LASSO model, and therefore taken forward to calculate the methylation profile score in external cohorts (as a weighted sum of methylation values). The EWAS catalog was searched using the 'ewascatalog' R package for other studies with n>1000 which reported significant CpG-trait associations.

File Name: Supplementary Data 16

Description: The background gene list provided to FUMA analysis, consisting of all the genes which are annotated to the Illumina EPIC array, extracted using the Infinium MethylationEPIC BeadChip database.

File Name: Supplementary Data 17

Description: The MSigDB GO Biological Processes Gene Sets (with more than 20 and less than 500 genes) which were tested for enrichment in the study.

File Name: Supplementary Data 18

Description: Demographic information for the antidepressant exposure phenotype cases (exposed) and controls (non-exposed) in external cohorts tested in this study. Age, BMI, pack years and AD MRS are presented as the mean (SD). Sex, smoking variables, and MDD status is given by the number of individuals (% of study). Note, in the absence of current/former/never smoking categories, pack years was used to assess smoking status. * MARS-UniDep smoking variables are available for the MARS cohort only. **The Never Smoking for MARS is NOT smoking at baseline (does not rule out previous smoking).

File Name: Supplementary Data 19

Description: A summary table of the DNA methylation data for each external cohort tested in the study. Cohorts used either 450K or EPIC arrays, and had a range of quality control criteria for the exclusion of samples and probes in the sample. The number of probes available for the calculation of the MPS and the missingness (if any) is also detailed.

File Name: Supplementary Data 20

Description: The association models for each cohort for testing the association between antidepressant exposure and the methylation profile score (calculated using weighted sum of CpGs identified from GS). The antidepressant exposure phenotype, model type and covariates included in the model are included, alongside Misc which details any additional details or model parameters which were specified.
