# Supplementary material for: Insights from a methylome-wide association study of antidepressant exposure
Source: Nat Commun. 2025 Feb 24;16:1908. doi: 10.1038/s41467-024-55356-x (PMC11850842; doi:10.1038/s41467-024-55356-x)
Supplement: Supplementary file 24 — Reporting Summary [file 41467_2024_55356_MOESM24_ESM.pdf]

Corresponding author(s): Andrew McIntosh
Ella Davyson

Last updated by author(s): Nov 25, 2025

Reporting Summary

Nature Portfolio wishes to improve the reproducibility of the work that we publish. This form provides structure for consistency and transparency in reporting. For further information on Nature Portfolio policies, see our Editorial Policies and the Editorial Policy Checklist.

Statistics

For all statistical analyses, confirm that the following items are present in the figure legend, table legend, main text, or Methods section.

n/a	Confirmed	
☐	☒	The exact sample size (*n*) for each experimental group/condition, given as a discrete number and unit of measurement
☐	☒	A statement on whether measurements were taken from distinct samples or whether the same sample was measured repeatedly
☐	☒	The statistical test(s) used AND whether they are one- or two-sided<br>*Only common tests should be described solely by name; describe more complex techniques in the Methods section.*
☐	☒	A description of all covariates tested
☐	☒	A description of any assumptions or corrections, such as tests of normality and adjustment for multiple comparisons
☐	☒	A full description of the statistical parameters including central tendency (e.g. means) or other basic estimates (e.g. regression coefficient) AND variation (e.g. standard deviation) or associated estimates of uncertainty (e.g. confidence intervals)
☐	☒	For null hypothesis testing, the test statistic (e.g. *F*, *t*, *r*) with confidence intervals, effect sizes, degrees of freedom and *P* value noted<br>*Give P values as exact values whenever suitable.*
☒	☐	For Bayesian analysis, information on the choice of priors and Markov chain Monte Carlo settings
☐	☒	For hierarchical and complex designs, identification of the appropriate level for tests and full reporting of outcomes
☐	☒	Estimates of effect sizes (e.g. Cohen's *d*, Pearson's *r*), indicating how they were calculated

Our web collection on statistics for biologists contains articles on many of the points above.

Software and code

Policy information about availability of computer code

Data collection	No formal software was used to collect the blood samples for DNA methylation datasets.
Data analysis	All code used in the analyses for this work as written by the authors is housed at : https://github.com/Elladavyson/Antidepressant_MWAS (https://doi.org/10.5281/zenodo.14185885). This is open access.<br><br>Software and R packages used in the analyses were as follows:<br><br>R (Version 4.1.0)<br>OSCA software (Version 0.46)<br>GCTA software (Version 1.94.0)<br>ewascatalog R package (Version 1.0)<br>BiocManager R package (Version 1.30.22)<br>IlluminaHumanMethylationEPICanno.ilm10b4.hg19 R package (Version 0.6.0)<br>missMethyl R package (Version 1.36.0)<br>biomaRt R package (Version 2.58.2)<br>ensembldb R package (Version 2.26.0)<br>msigdbr R package (Version 7.5.1)<br>lubridate R package (Version 1.9.3)<br>stringr R package (Version 1.5.1)

stringi R package (Version 1.8.3)
dmrff R package (Version 1.1.1)
stats R package (Version 4.1.0)
biglasso R package (Version 1.5.2)

For manuscripts utilizing custom algorithms or software that are central to the research but not yet described in published literature, software must be made available to editors and reviewers. We strongly encourage code deposition in a community repository (e.g. GitHub). See the Nature Portfolio guidelines for submitting code & software for further information.

Data

Policy information about availability of data

All manuscripts must include a data availability statement. This statement should provide the following information, where applicable:

- Accession codes, unique identifiers, or web links for publicly available datasets
- A description of any restrictions on data availability
- For clinical datasets or third party data, please ensure that the statement adheres to our policy

The data generated in this study (MWAS summary statistics, downstream functional results and weights in the MPS) have been deposited in a Zenodo repository (14203229 [DOI: 10.5281/zenodo.14203229])78. Additionally, the source data for main figures and all supplementary figures which are not sharing individual-level data are provided in this repository. The raw data from all cohorts (Generation Scotland, Stratifying Anxiety and Depression Longitudinally, Netherlands Study of Anxiety and Depression, Finnish Twin Cohort, Study of Health in Pomerania, FOR2107, Netherlands Twin Register,, Munich Antidepressant Response Study, UniPolar Depression Study, Avon Longitudinal Study of Parents and Children, E-risk Longitudinal Twin Study and Lothian Birth Cohorts) are not publicly available due to them containing information that could compromise participant consent and confidentiality. Access information for each cohort can be found below.

Generation Scotland is run as a resource for the research community. Requests to use the Resource are made from: Academic collaborators: employees who are party to the Generation Scotland Collaboration Agreement, or researchers or employees of an academic institution or the NHS. Commercial organisations: specific arrangements have been defined to allow commercial organisations to access Generation Scotland resources. Data can be obtained from the data owners. Instructions for accessing Generation Scotland data can be found here: https://www.ed.ac.uk/generation-scotland/for-researchers/access; the GS Access Request Form can be downloaded from this site. Completed request forms must be sent to access@generationscotland.org to be approved by the Generation Scotland Access Committee. Upon submission, applications are reviewed within 6-8 weeks. For any further correspondence and material requests please contact access@generationscotland.org.

The Netherlands Study of Anxiety and Depression (NESDA) is run as a resource for the research community and is open to data-use requests from bona fide international researchers. Instructions for gaining access to NESDA can be found here: https://www.nesda.nl/nesda/wp-content/uploads/2024/09/NESDA_policy_data_access.pdf. Applications involve submitting a research proposal, including specific research questions, methodology and proposed statistical analysis, to the NESDA management committee (nesda@amsterdamumc.nl). Data access forms can be downloaded here: www.nesda.nl. The review process by the management committee for data access may take up to 6 weeks.

E-Risk is run as a resource for the research community and is free to access by researchers from all over the world who are based at universities or research institutions. E-Risk operates a managed access process to protect the privacy of the participants. Instructions for accessing The Environmental-Risk Longitudinal Twin Study (E-Risk) data can be found here: https://eriskstudy.com/data-access. In brief following the reading of a data-sharing protocol, a concept paper will need to be submitted outlining the proposed analyses and required variables via this link: https://redcap.link/ERiskConceptPaperForm for consideration by the E-Risk Steering Committee. A decision will usually be made within 1 month. Once approved, the concept paper will be made public and the data sent securely to those requiring access within 1-2 months after a formal data use agreement has been signed. For any questions regarding the data available and the access process, please email eriskstudy@kcl.ac.uk.

The data of the Study of Health in Pomerania study cannot be made publicly available due to the informed consent of the study participants, but it can be accessed through a data application form available at https://transfer.ship-med.uni-greifswald.de/ for researchers who meet the criteria for access to confidential data. In detail, access to the data requires a proposal submitted to the University Medicine Greifswald represented by the steering committee of the Research Network Community Medicine (FVCM), which meets once a month. A successful data application provides data usage permission for three years at maximum and needs to be extended afterwards if needed.

In accordance with the Finnish Biobank Act, the data used in the analysis from the Finnish Twin Cohort (FTC) is deposited in the Biobank of the Finnish Institute for Health and Welfare (https://thl.fi/en/research-and-development/thl-biobank/for-researchers). It is available to researchers from academia and companies after written application and following the relevant Finnish legislation. To ensure the protection of privacy and compliance with national data protection legislation, a data use/transfer agreement is needed, the content and specific clauses of which will depend on the nature of the requested data. Allow 2-4 weeks for initial response from the Biobank.

Avon Longitudinal Study of Parents and Children (ALSPAC) is run as a resource for the research community. Instructions for accessing ALSPAC data can be found here: https://www.bristol.ac.uk/alspac/researchers/access/. A research proposal must be submitted via the research proposal system for consideration by the ALSPAC Executive Committee. For any questions regarding accessing data or samples please email alspac-data@bristol.ac.uk (data) or bbl-info@bristol.ac.uk (samples). Approval may take up to two weeks.

Lothian Birth Cohort 1936 (LBC1936) is run as a resource for the research community which actively collaborates with research experts in the UK and internationally. Instructions for how to access LBC1936 data can be found here: https://lothian-birth-cohorts.ed.ac.uk/data-access-collaboration. In brief, identify the variables you will require for the analysis from the LBC1936 data dictionary (which can be downloaded from the link). Following this, prepare a data request form including the provisional title of the study, the principle researcher and institution and the brief rationale for the study, research method and main variables. Email the LBC request form to Professor Simon Cox (simon.cox@ed.ac.uk), the director of Lothian Birth Cohorts for approval from the study investigator team. Approval may take up to 4 weeks. Data will be shared on the basis of a Data or Material Transfer Agreement between provider and recipient institutions.

The Netherlands Twin Register (NTR) is run as a resource for the research community and is open to data-use requests from bona fide international researchers. Information on accessing NTR data can be found here: https://ntr-data-request.psy.vu.nl/. To submit a data sharing request, complete a data sharing request form (https://ntr-data-request.psy.vu.nl/DSR-forms.html) and send it to NTR Data management team (ntr.datamanagement.fgb@vu.nl), which will check the request for feasibility and completeness and pass on the request to the data access committee (DAC) for approval. The review process by the DAC may take up to 4 weeks.

The raw data collected in the FOR2107 study is not openly accessible to protect participant consent and confidentiality. Nevertheless, the FOR2107 study serves as a valuable resource for the global research community. It is accessible, in principle, to all scientific
researchers affiliated with non-commercial research organizations worldwide. Researchers seeking access to the study's data must submit a formal research proposal. This proposal should outline the specific research questions, methodology, and planned statistical analyses. Applications are reviewed by the principal investigators of FOR2107, Professors Tilo Kircher (tilo.kircher@staff.uni-marburg.de) and Udo Dannlowski (udo.dannlowski@uni-muenster.de), within 6–8 weeks of

submission.
The raw data collected in the Munich Antidepressant Response Study-Unipolar Depression Study (MARS-UniDep) is not publicly available but can be shared with researchers upon request. Access to the data can be requested in a data transfer agreement, please contact Darina Czamara (darina@psych.mpg.de). Only research questions related to psychiatric disorders can be addressed directly. The expected time frame for response to access requests is 2 weeks. Data access will be granted until the end of the requested project, that is upon publication of the related manuscript.

Research involving human participants, their data, or biological material

Policy information about studies with human participants or human data. See also policy information about sex, gender (identity/presentation), and sexual orientation and race, ethnicity and racism.

Reporting on sex and gender

Information on the self-reported biological sex of participants was collected as part of Generation Scotland baseline recruitment and used as a covariate in this main MWAS analysis. The numbers of female/ male-sex participants in each phenotype group used in these analyses are shown in Table 1. Additionally, sex-stratified analysis of self-reported antidepressant exposure was performed using the self-report variable of sex.

Reporting on race, ethnicity, or other socially relevant groupings

The participants in Generation Scotland cohort predominantly report being 'White British' (> 97%). Multi-dimensional scaling (MDS) of Generation Scotland alongside HapMap3 (HM3) shows the cluster of Generation Scotland samples completely overlaying the cluster of HM3 European samples in the first two MDS components. Therefore, in these analyses we did not covary for race/ethnicity. Confounding factors which were included in the analysis as covariates were:
Age (years)
Biological sex (self-reported)
M values at the AHRR probe ('cg05575921'), which act as an established marker for smoking status (DNA methylation data)
Predicted monocyte and lymphocyte cell proportions (DNA methylation data)

The summary characteristics of Age, Sex, BMI, smoking behaviours and lifetime depression status are shown per phenotype group in Table 1.

Population characteristics

Main cohort: Generation Scotland (GS)

GS is a family-based cohort study with DNA, and socio-economic and clinical data from ~ 24,000 volunteers across Scotland aged 18-65 years, collected from February 2006 to March 2011.

Prospective GS cohort used in the antidepressant exposure methylation profile score (MPS) analysis: Stratifying Resilience and Depression Longitudinally (STRADL).

The STRADL cohort is a subset of 1,188 individuals from the GS cohort who undertook additional assessments approximately five years after the study baseline.

External cohorts used in the antidepressant exposure MPS analysis:

Finn Twin Cohort (FTC):
FTC comprises of three separate longitudinal subcohorts, the Older Finnish Twin Cohort FinnTwin 12 and FinnTwin16. The older cohort consists of twin pairs born before 1958 (n = 13,388). FinnTwin12 and FinnTwin16 are longitudinal twin studies of twin pairs born between 1983-1987 (n= 2,700) and 1975-79 (n = 2,800) respectively. All cohorts have extensive data collection including behavioral and lifestyle traits over multiple waves.

Study of Health in Pomerania (SHIP)- Trend:
SHIP-Trend is a population-based cohort with data including in-depth interviews, imaging and biological sampling conducted between September 2008 and September 2012.

FOR2107:
FOR2107 is a research programme including a human cohort (target n = 2,500) and animal models. The human cohort consists of those with bipolar, schizophrenia schizoaffective disorder and healthy controls with/withour genetic and environmental risk factors for affective disorders. Participants undergo an extensive data collection including biological samples and in-depth questionnaires, which began in November 2014 and is ongoing.

Netherlands Twin Register (NTR):
NTR is a population-based cohort of over 200,000 people across the Netherlands, consisting of twin-families aged between 0-99 at recruitment, and began in 1987. This study used DNA methylation collected in the NTR-Biobank study, in which 9,520 participants from 3,477 families in NTR provided biological sample alongside further phenotypic information in a home-visit assessment between July 2004 and July 2008.

Munich Antidepressant Response (MARS) / Unipolar Depression study (UniDep):
The MARS study is a naturalistic longitduinal clinical study of adult Caucasian inpatients (aged between 18 and 75 years) admitted to psychiatric hospitals in an acute episode of major depression in southern Germany. Data was collected between 1995 and 2005. The UniDep study is a cross-sectional case-control study in Germany consisting of German in and outpatients (n=1000, 67.4% female) with recurrent MDD from the Max-Planck Institute of Psychiatry in Munich and psychiatric hospitals in Augsburg and Ingolstadt with each hospital contributing a third of the patients. A control sample was randomly selected

from a Munich-based community sample, screened for the presence of anxiety or affective disorders using the composite international diagnostic screener (CIDI) and matched by ethnicity, age and sex to those in the cases sample. Baseline data collection was collected between 2002 and 2004.

Lothian Birth Cohort (LBC1936):
LBC1936 is a population cohort of individuals who took the Scottish Mental Surveys in 1947 (N = 1,091). Initial baseline assessment was conducted in a single visit to Western General Hospital in Edinburgh between November 2006 and May 2007.

Avon Longitudinal Study of Parents and Children (ALSPAC):
ALSPAC is a transgenerational prospective observational study beginning with the recruitment of pregnant women resident in the Avon, UK (N = 14,541). Extensive data collection has occurred at multiple time points for the mothers and children in the cohort. Please refer to the searchable data dictionary and variable search tool (http://www.bristol.ac.uk/alspac/researchers/our-data/). A subsample of ALSPAC children, mothers and partners participated in the Accessible Resource for Integrated Epigenomic Studies (ARIES) had their DNAm assayed and are used in this study.

Environmental risk (E-risk) Longitudinal Twin Study:
E-risk is a representative birth cohort study of 2,232 twins born in England and Wales between 1994-1995. Baseline data collection occurred between 1990-2000 when 1,116 families with same-sex 5 year old twins participated in home-visit assessments. This is a nationally representative sample. Follow-up home visits were conducted at age 7,10,12 and 18. The DNA methylation data used in this study was collected at the age-18 home visit.

Enrichment : Netherlands Study of Anxiety and Depression
The Netherlands Study of Depression and Anxiety is a naturalistic ongoing longitudinal cohort study which aims to investigate the long-term course and consequences of depression and anxiety in the Netherlands. Recruitment took place in the general population across various health care settings (community, primary care and specialised mental health care) for individuals between 18 and 65 years of age.
Population demographics can be found in Supplementary Data 18 for each cohort used in the analyses.

Recruitment

Generation Scotland (GS):

Potential participants were identified at random from those aged 35-65 years from the lists of collaborating general practice medical practices and invited to participate. They were also invited to identify at least one first degree relative aged at least 18 years who would also participate. Glasgow and Tayside were the initial recruitment areas in Scotland, but this was extended to include Ayrshire, Arran and Northeast Scotland alongside a broadening of the age range of participants in the later stages of the study. Beyond invitation, volunteers were also welcomed to the study if they were over 18 years of age and had one first degree relative who could also participate. Participants filled out a pre-clinical questionnaire, and subsequently attended an extensive in-person clinic where physical measurements, biological sampling, and a psychiatric assessment was performed. Participants were informed that the nature of the study was to investigate the health of the Scottish population and gave consent before any sampling took place.

FinnTwinCohorts (FTC):

Older Twin Cohort: Questionnaires were mailed in 1975 and 1981 to all twins born before 1958 and living in Finland, identified from the population register of Finland.
FinnTwin12: Questionnaires were mailed to twins born between 1983 and 1987 identified through Finland's Central Population Registry (CPR). Return of the questionnaire had permission to contact the twin children for subsequent assessment.
FinnTwin16: Questionnaires were mailed to twins born between 1975 and 1979 identified through Finland's Central Population Registry (CPR). Return of the questionnaire had permission to contact the twin children for subsequent assessment.

Study of Health in Pomerania (SHIP)-Trend:

From the total population of West Pomerania, a stratified sample of 8,016 adults was aged 20-79 years was drawn for SHIP-TREND between 2008 and 2011, facilitated by the local population registries in the Federal State of Mecklenburg/West Pomerania. Stratification variables are age, sex, and city/county of residence.

FOR2107:
Those with affective disorders are recruited via in and out-patient services in Marburg and Münster, Germany. Healthy control participants are recruited via newspaper advertisements. Recruitment began in 2014 and is ongoing.

Netherlands Twin Register (NTR)
The recruitment into NTR is inclusive with no restrictions to enrollment. Recruitment began in 1986, with the systematic approach of parents to register newborn twins using a commercial 'birth felicitation' service. Additional recruitment of newborn twins and triplets and their parents is done with the support of the Dutch Society of Parents of Multiples.

Munich Antidepressant Response study (MARS)/Unipolar Depression Study (UniDep):
MARS study collected longitudinal data on depressed inpatients from three clinical sites in southern Bavaria (Max Planck

Institute of Psychiatry, Munich; Bezirkskrankenhaus Augsburg; Klinikum Ingolstadt). The project is ongoing and further sites have been invited to participate.
UniDep case sample was recruited from in and out patients in Max Planck Institute of Psychiatry and psychiatric hospitals in Augsburg and Ingolstadt (each hospital contributing a third of the patients). The control sample was recruited at the Max Palanck Institute of Pyschiatry, randomly selected from a Munich-based community sample and screen for the presence of anxiety and depression using the Cmposite International Diagnostic Screener.

Lothian Birth Cohort 1936:
The Scottish Mental Surveys were distributed to 11 year olds residing in Scotland in 1947. Between 2004 and 2007, individuals from Edinburgh and the Lothians who might have taken part in the 1947 survey were identified using the Community Health Index, and invited to participate in LBC1936 (N = 1,091). Participants were recruited to the study at a mean age of 70 years, and have completed various waves of assessment including a series of cognitive, clinical, physical and social data alongside biological samples.

Avon Longitudinal Study of Parents and Children:
Initial recruitment involved contacting pregnant women resident in Avon, UK with expected delivery dates between 1st April 1991 and 31st December 1992 were invited to take part in the study, resulting in 13,988 children alive after 1 year. An additional recruitment occurred when the children were 7 years of age, recruiting eligible cases which were not recruited originally. Together, there were 14,901 children alive at one year of age recruited into the study.

Environmental risk (E-Risk) Longitudinal Twin Study
The E-risk sampling frame was same-sex twins present in two consecutive birth cohorts (1994 and 1995) in the Twins' Early Development Study (TEDS), a birth register of twins born in England and Wales. The full register is administered by the governments Office of National Statistics (ONS), which invited parents of all twins born in 1994-95 to enroll in TEDS. E-Risk study targeted 1,210 families, which was drawn using a high-risk stratification sampling frame. High risk families were those in which the mother had her first birth when she was 20 years of age or younger.

Netherlands Study of Anxiety and Depression
Recruitment took place in the general population across various health care settings (community, primary care and specialised mental health care) for individuals between 18 and 65 years of age. The recruited sample consists of 1,701 individuals with current diagnosis of depression and/or anxiety, 907 individuals with lifetime diagnoses or at risk due to family history of subthreshold symptoms and 373 healthy controls. A four-hour baseline assessment was conducted and included written questionnaires, interviews, a medical examination, cognitive tasks, blood and saliva sampling and intensive screening regarding mental health outcomes. Detailed assessments were then repeated after one, two, four and eight years of follow-up.

Although many of these studies employ a recruitment strategy based through national health registries and community centers to facilitate random and representative sampling of the whole population, they are inherently self-selecting, as participants voluntarily consent to being part of the study. Therefore, this could lead to a self-selection bias in the results towards the demographic which is more represented in these cohorts (typically white european, female and of a higher than average socio-economic status).

Ethics oversight

Generation Scotland:
Ethical approval was provided by the Tayside Research Ethics Committee (REC reference 05/S1401/89). GS has also been granted Research Tissue Bank status by the East of Scotland Research Ethics Service (REC Reference Number: 20/ES/2001), providing generic ethical approval for a wide range of uses within medical research. All participants included in the current study provided informed consent for the use of their data for biomedical research.

STRADL:

All components of STRADL received formal, national ethical approval from the NHS Tayside committee on research ethics (reference 14/SS/0039). All participants included in the current study provided informed consent for the use of their data for biomedical research.

FinnTwin Cohort:
Participants were given information on the study procedures and of freedom to participate or to decline at any point in both oral and written form. Informed consent was obtained upon the contact with the study subjects before new questionnaire information was collected, and when clinical investigations were undertaken with sampling of biological material. Ethics approvals have been granted for multiple studies concerning the FTC twins by the ethics committees of Helsinki University Central Hospital (113/E3/2001, 249/E5/2001, 346/E0/05, 270/13/03/01/2008, and 154/13/03/00/2011) with the last one on the transfer of biological samples to the THL Biobank in 2018 (HU51179912017).

SHIP:
Participants provided written informed consent before any assessment and/or sampling took place. The Ethics Committee of the University Medicine Greifswald, Germany provided ethical approval for the study (BB 39/08).

FOR2107:
Participants provided written consent before any assessment and/or sampling took place. The ethics committees of the Medical Faculties, University of Marburg (AZ: 07/14) and University of Münster (2014-422-b-S) provided ethical approval for the study.

NTR:

Informed consent was obtained from all participants. The study was approved by the CentraLBCl Ethics Committee on Research Involving Human Subjects of the VU university Medical Centre, Amsterdam, an Institutional Review Board certified by the U.S Office of Human Research Protections (IRB number IRB00002991 under Federal-wide Assurance-FWA00017598; IRB/institute codes, NTR 03-180).

MARS:
The study was approved by the local Ethics Committee of the Ludwig Maximilians University, Munich, Germany, and carried out in accordance with the latest version of the Declaration of Helsinki. All participants provided written consent after the study protocol and potential risks were explained.

UniDep:
The study was approved by the Ethics committee of the Ludwig Maximillans University in Munich, Germany and written informed consent was obtained from all subjects.

LBC1936:
Ethical approval was obtained from the Multicentre Research Ethics Committee for Scotland (baseline, MREC/01/0/56), the Lothian Research Ethics Committee (age 70, LREC/2003/2/29), and the Scotland A Research Ethics Committee (ages 73, 76, 79, 07/MRE00/58). All participants provided written informed consent.

ALSPAC:
Ethical approval for the study was obtained from the ALSPAC Ethics and Law Committee and the Local Research Ethics Committees. Consent for biological samples has been collected in accordance with the Human Tissue Act (2004). Informed written consent was provided by all participants.

E-Risk:
The Joint South London and Maudsley and Institute of Psychiatry Research Ethics Committee approved each study phase. Parents gave informed consent, and twins gave assent between 5 and 12 years and then informed consent at age 18.

NESDA:
The study protocol was approved centrally by the Ethical Review Board of the VU University Medical Centre and subsequently by local review boards of each participating centre. After full verbal and written information about the study, written informed consent was obtained from all participants at the start of baseline assessment.

Note that full information on the approval of the study protocol must also be provided in the manuscript.

Field-specific reporting

Please select the one below that is the best fit for your research. If you are not sure, read the appropriate sections before making your selection.

☒ Life sciences ☐ Behavioural & social sciences ☐ Ecological, evolutionary & environmental sciences

For a reference copy of the document with all sections, see nature.com/documents/nr-reporting-summary-flat.pdf

Life sciences study design

All studies must disclose on these points even when the disclosure is negative.

Sample size

Main MWAS analysis (Generation Scotland):

Self-report antidepressant exposure analysis: N = 16,536, Nexposed = 1,508, Nunexposed = 5,028
Self-report analysis (MDD-only subset of above sample): N = 2,268, Nexposed = 766, Nunexposed = 1,502
Self-report analysis (female sex only): N = 9710, Nexposed = 8,556, Nexposed = 1154
Self-report analysis (male sex only): N = 6821, Nexposed = 6,467, Nunexposed = 354
Prescription derived antidepressant exposure analysis: N = 7,951, Nexposed = 861, Nunexposed = 7,090
Prescription derived analysis (MDD only subset of above sample): N = 792, Nexposed = 380, Nunexposed = 412

Enrichment analysis (MBDSeq in NESDA)
NESDA: Nexposed = 398, Nunexposed = 414

Antidepressant exposure methylation profile score analysis (External datasets):

FinnTwin: N = 1,678, Nexposed = 84, Nunexposed = 1,594
SHIP: N = 495, Nexposed = 21, Nunexposed = 474
FOR2107: N = 658, Nexposed = 165, Nunexposed = 493
NTR: N = 3,004, Nexposed = 87, Nunexposed = 2,917
MARS/UniDep: N = 312, Nexposed = 135, Nunexposed = 177
LBC1936: N = 889, Nexposed = 46, Nunexposed = 843
ALSPAC: N = 801, Nexposed = 43, Nunexposed = 758

E-Risk: N = 1658, Nexposed = 36, Nunexposed = 1622
STRADL: N = 663, Nexposed = 46, Nunexposed = 617

Data exclusions

All datasets excluded participants which did not have matching DNAm sample which passed the quality control filters or did not have an antidepressant exposure phenotype.

Generation Scotland:
In the MDD-only groups, participants were excluded if they fulfilled the criteria for bipolar disorder using the Structured Clinical Interview for DSM-IV Non-Patient Version (SCID). As prescription linkage in GS began in 2009, those enrolled and assessed before linkage were excluded from the prescription-derived phenotype (due to their being a lack of information regarding the antidepressant exposure at the time of DNAm measurement. All other data were included.

Other cohorts:

FinnTwin ,SHIP-Trend, NTR, LBC1936, ALSPAC, NESDA and E-Risk have no further exclusion criteria.

For the cohorts which use in and out patient cohort samples there were further exclusion criteria:

FOR2107: Individuals diagnosed with bipolar, schizophrenia or schizoaffective disorder were excluded from the analysis.
MARS: Exclusion criteria for this cohort was having depressive syndromes secondary to any medical or neurological condition, the presence of manic, hypomanic of mixed affective symptoms, lifetime diagnosis of alcohol dependence, illicit drug abuse or the presence of severe medical conditions.
UniDep: In the case samples, those with the presence of manic of hypomanic episodes, mood incongruent psychotic symptoms, the presence of a lifetime diagnosis of intravenous drug abuse and depressive symptoms which are secondary to a substance abuse disorder or due to medical illness or medication were excluded from the study.

Replication

Direct replication in an external cohort of the MWAS findings are not available in this study. There are no cohorts (to our knowledge) which are freely available and have both DNA methylation data and antidepressant exposure variables in a sample size comparable to Generation Scotland, which could facilitate this analysis. Therefore, in this study we tested the generalisability of the findings in GS through calculating methylation profile scores (using weights derived from GS) and testing the association with antidepressant exposure in external cohorts.

Randomization

N/A. No randomisation was required. All participants were included and there were no experimental groups.

Blinding

N/A. This study did not include the allocation of experimental groups and profiled the associations between antidepressant exposure and DNA methylation.

Reporting for specific materials, systems and methods

We require information from authors about some types of materials, experimental systems and methods used in many studies. Here, indicate whether each material, system or method listed is relevant to your study. If you are not sure if a list item applies to your research, read the appropriate section before selecting a response.

Materials & experimental systems

n/a	Involved in the study	
☒	☐	Antibodies
☒	☐	Eukaryotic cell lines
☒	☐	Palaeontology and archaeology
☒	☐	Animals and other organisms
☒	☐	Clinical data
☒	☐	Dual use research of concern
☒	☐	Plants

Methods

n/a	Involved in the study	
☒	☐	ChIP-seq
☒	☐	Flow cytometry
☒	☐	MRI-based neuroimaging

Plants

Seed stocks

Report on the source of all seed stocks or other plant material used. If applicable, state the seed stock centre and catalogue number. If plant specimens were collected from the field, describe the collection location, date and sampling procedures.

Novel plant genotypes

Describe the methods by which all novel plant genotypes were produced. This includes those generated by transgenic approaches, gene editing, chemical/radiation-based mutagenesis and hybridization. For transgenic lines, describe the transformation method, the number of independent lines analyzed and the generation upon which experiments were performed. For gene-edited lines, describe the editor used, the endogenous sequence targeted for editing, the targeting guide RNA sequence (if applicable) and how the editor was applied.

Authentication

Describe any authentication procedures for each seed stock used or novel genotype generated. Describe any experiments used to assess the effect of a mutation and, where applicable, how potential secondary effects (e.g. second site T-DNA insertions, mosiacism, off-target gene editing) were examined.
